# Antibacterial and Antifungal Compounds from Marine Fungi

**DOI:** 10.3390/md13063479

**Published:** 2015-06-02

**Authors:** Lijian Xu, Wei Meng, Cong Cao, Jian Wang, Wenjun Shan, Qinggui Wang

**Affiliations:** 1 College of Agricultural Resource and Environment, Heilongjiang University, Harbin 150080, China; E-Mails: caoconghlju@sina.cn (C.C.); wangjianhjlu@sina.com (J.W.); shanwenjunhlju@sina.com (W.S.); 2 College of Life Science, Northeast Forestry University, Harbin 150040, China; E-Mail: mwneau@gmail.com

**Keywords:** marine fungus, antimicrobial, antibacterial, antifungal, antibiotic, *Aspergillus*, polyketide, metabolites

## Abstract

This paper reviews 116 new compounds with antifungal or antibacterial activities as well as 169 other known antimicrobial compounds, with a specific focus on January 2010 through March 2015. Furthermore, the phylogeny of the fungi producing these antibacterial or antifungal compounds was analyzed. The new methods used to isolate marine fungi that possess antibacterial or antifungal activities as well as the relationship between structure and activity are shown in this review.

## 1. Introduction

Antibacterials and antifungals are among the most commonly used drugs. Recently, as the resistance of bacterial and fungal pathogens has become increasingly serious, there is a growing demand for new antibacterial and antifungal compounds. Natural products from fungi are considered an important source for novel antibacterial and antifungal compounds because of their abundant fungal species diversity, their rich secondary metabolites and the improvements in their genetic breeding and fermentation processes. The antimicrobial activities of an increasing number of fungi living in distinctive environments are being investigated for the discovery of new antibacterial and antifungal compounds, such as endophytic fungi from wild plants and marine fungi. In the last decade, many novel bioactive natural products from marine fungi have been discovered that possess cytotoxic, anticancer, antiviral, antibacterial or antifungal activities [1,2,3,4,5,6]. The antibacterial and antifungal compounds from marine fungi have quickly increased since 2010, and marine fungi have been an important source of antibacterial and antifungal compounds. This paper reviews the antibacterial and antifungal compounds from marine fungi with specific focus on the period from January 2010 to March 2015.

## 2. Sampling Location

**Figure 1:**
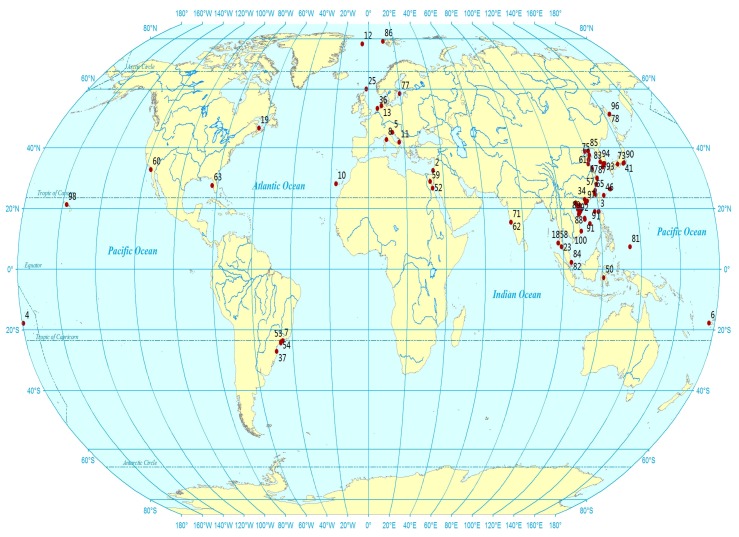
The approximate location of material collections for fungal isolation.

Marine fungi are an ecologically rather than physiologically or taxonomically defined group of organisms [1]. Marine fungi are parasitic or saprophytic in other marine organisms or materials. We collected 117 peer-reviewed research articles regarding antibacterial or antifungal compounds from marine fungi from January 2010 to March 2015. Most of the sites shown in Figure 1 are approximate locations based on the information about the marine material samplings in the literature and the other sites plotted by their exact latitude and longitude (details shown in Supplementary Table S1). There are 17 literature reports without collection locale information, and they are not included in Figure 1. According to Figure 1, most of the materials for fungal isolation were obtained near the coastal area of Eurasia, and more than a half of the marine materials are from the coastal area near China. Many natural products are used as medicines in China, and the Chinese people are traditionally keen on the discovery and development of natural medicines.

## 3. Fungal Isolation and Identification

**Figure 2:**
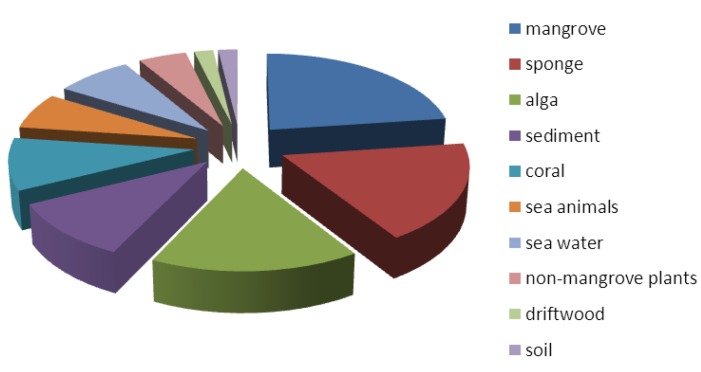
Numbers of fungal strains from different isolation materials.

Many different types of marine materials were collected for the fungal isolations. According to the literature, there are 105 marine fungal strains used for the isolation of antibacterial or antifungal compounds. These 105 marine fungal strains were isolated from marine materials, which are divided into 12 classes as shown in Figure 2.

Algae, sponges and mangroves are the most common materials for the isolation of fungal strains that can produce antibacterial or antifungal compounds. The fungi associated with these algae, sponges and mangroves are expected to produce compounds with novel or special skeletons because of the special interactions between the fungi and the algae, sponges or mangroves. Additionally, 20 of the 116 new compounds with antifungal or antibacterial activities are from the 11 fungal strains from marine sediments, indicating that these sediments are also good materials for the isolation of fungi. Therefore, based on the data from the past five years for the number of new compounds with antifungal or antibacterial activities, fungi isolated from sediments seem to be underestimated and fungi from sponges may be overestimated (21 new compounds with antibacterial and antifungal activities from 19 fungal strains from sponges). The information about the sampling sites, fungal sources and taxonomic names are shown in the Supplemental Material.

Over 700 compounds in total were purified from 105 fungal strains that can produce antimicrobial compounds and were investigated for these activities. There are 285 compounds (approximately 40% of the total) that showed antibacterial or antifungal activities and 116 (15% of the total) are new antibacterial and antifungal compounds. On average, more than one new compound with antibacterial and antifungal activities could be isolated from one fungal strain. According to the antimicrobial screening of marine fungal extracts from four literature reports [7,8,9,10], 38%–59% of the test extracts from marine fungi exhibited antibacterial or antifungal activities. Taken together, these data indicate that marine fungi are a good source of natural antibacterial and antifungal compounds.

Most of the 105 marine fungi with antibacterial or antifungal compounds were identified, and approximately 50% of them were identified based on their DNA sequence. The dominant genera in the marine fungi producing antibacterial and antimicrobial compounds were the *Aspergillus* genus (31 strains) and the *Penicillium* genus (16 strains).

## 4. Phylogenetic Analysis

**Figure 3:**
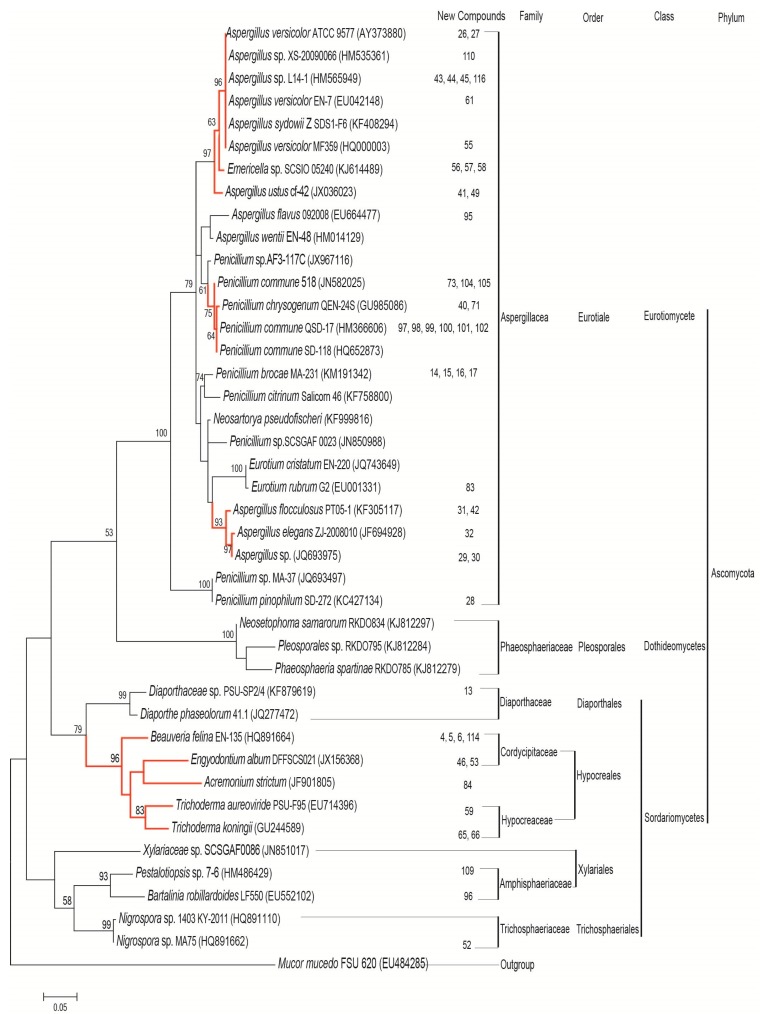
Phylogenetic analysis of marine fungi produced antibacterial or antifungal compounds.

To obtain an overview of the phylogenetic relationship among the marine fungi that produce antibacterial or antifungal compounds, we analyzed the internal transcribed spacer (ITS) data sets of 41 sequences from marine fungi with antibacterial or antifungal activities. Originally, 49 fungal DNA sequences were collected from the 117 literature reports. Eight of these sequences were calmodulin, tubulin or 18S ribosomal RNA genes, and the rest were the 41 ITS sequences used for phylogeny analysis. The ITS sequences were aligned by ClustalW implemented in BIOEDIT ver. 7.0.5 [11], and optimized manually using BIOEDIT. Maximum likelihood (ML) analysis was constructed using the Kimura 2-parameter (K2) nucleotide substitutions model as selected by MEGA6 [12]. A ML tree was generated using MEGA6 with bootstrap values calculated from 100 replicates (Figure 3). The phylogenetic tree was rooted with the *Mucor mucedo* ITS sequence (shown in Figure 3).

In the data from the last five years (January 2010 to March 2015), the marine fungi producing antibacterial or antifungal compounds did not display as high of a diversity as expected. All sampled marine fungi came from Ascomycota and were limited to Eurotiomycetes, Dothideomycetes, and Sordariomycetes. Most fungi belonged to the Eurotiomycetes, forming a well-supported clade (Figure 3: BS = 100%). Compared with the Sordariomycetes, the Dothideomycetes and Eurotiomycetes were similar to each other with weak support (Figure 3: BS = 53%). Although the Diaporthales, Hypocreales, Xylariales, and Trichosphaeriales are from the same class, they diverged into two distinct clades. Diaporthales and Hypocreales appeared to form a moderately supported clade (Figure 3: BS = 79%).

Of the new compounds with antibacterial or antifungal activities purified from these 41 fungal strains, 51 were also listed in Figure 3. According to Figure 3, the fungal strains from Aspergillacea produced 31 of the polyketides (including the N-containing polyketides) and 6 of the steroids and terpenoids. Therefore, it is possible that the main antibacterial and antifungal compounds of the marine fungal strains are from Aspergillacea are polyketides, but beyond that, it is difficult to find a correlation between the types of new compounds and the phylogeny of the marine fungi. However, it is interesting to note that the quantity of new antibacterial and antifungal compounds may be related to the phylogeny of the marine fungi. Moreover, none of the new antibacterial or antifungal compounds purified were from Pleosporales. However, the 20 fungal strains from the four phylogenic groups highlighted in red in Figure 3 produced over 80% of the new antibacterial and antifungal compounds. New antibacterial and antifungal compounds were purified from all five strains from Hypocreales. Two *Aspergillus* spp. (of the three total *Aspergillus* spp.) and one *Penicillium* sp. (of five) were also good sources for new antibacterial and antifungal compounds. Therefore, the marine fungal strains from Hypocreales and the *Aspergillus* and *Penicillium* genera should be utilized more for the discovery of new antibacterial or antifungal compounds.

## 5. New Antibacterial and Antifungal Compounds from Marine Fungi

### 5.1. Nitrogen-Containing Compounds

#### 5.1.1. Peptides

**Figure 4:**
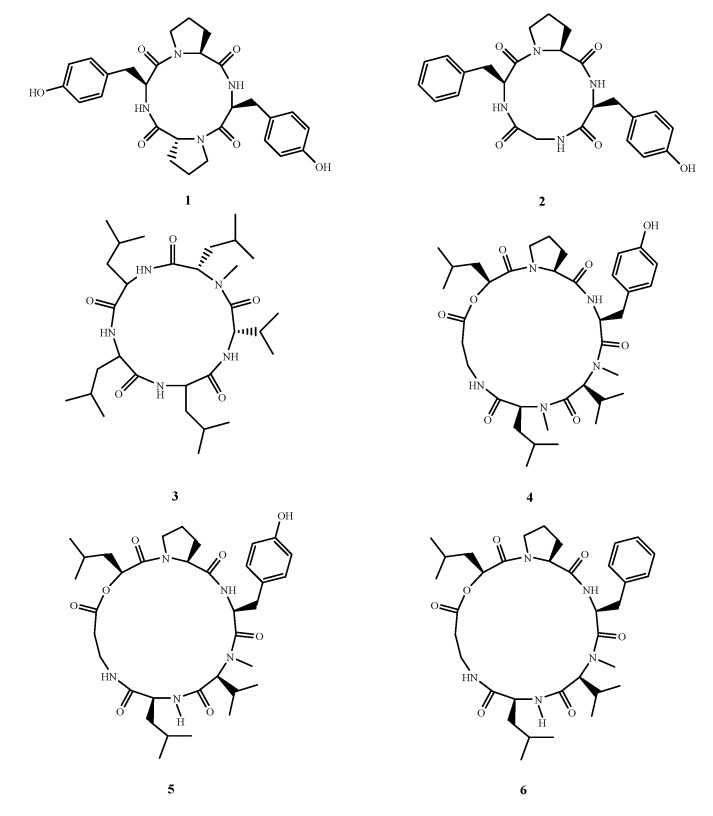
Structures of compound **1**–**6**.

Cyclopeptides from terrestrial microorganisms are considered a good antimicrobial source. However, only six new antimicrobial cyclopeptides, **1**–**6** (Figure 4), were isolated from three marine fungi. Two cyclotetrapeptides (d-Pro-l-Tyr-l-Pro-l-Tyr) (**1**) and (Gly-l-Phe-l-Pro-l-Tyr) (**2**), were isolated from the co-culture broth of mangrove fungi *Phomopsis* sp. K38 and *Alternaria* sp. E33 [13]. Compound **2** showed stronger activity (MIC, 25–250 μg/mL) than **1** (35–400 μg/mL) against five tested fungi. Cyclopentapeptide lajollamide A (**3**) was isolated from *Asteromyces cruciatus* 763. Natural lajollamide A (**3**), which is a mixture of several stereochemical configurations, has only weak antibacterial activity against *Bacillus subtilis* and *Staphylococcus epidermidis* [14]. However, the absolute configuration of lajollamide A (**3**) (leucine residues 1–3: d-Leu-l-Leu-l-Leu), which was solved by total synthesis, was more active than natural lajollamide A (**3**). This indicates that the diastereomers of the Leu-Leu-Leu moiety effectively impact the antibacterial activity. Isaridin G (**4**), desmethylisaridin G (**5**) and desmethylisaridin C1 (**6**) are three new cyclohexadepsipeptides of the isaridin class that were isloated from *Beauveria felina* EN-135. These compounds showed inhibitory activity against *Escherichia coli* (MIC, 64, 64 and 8 μg/mL, respectively) and this is the first report of antibacterial activity of the isaridins [15].

#### 5.1.2. Indole-Alkaloids

**Figure 5:**
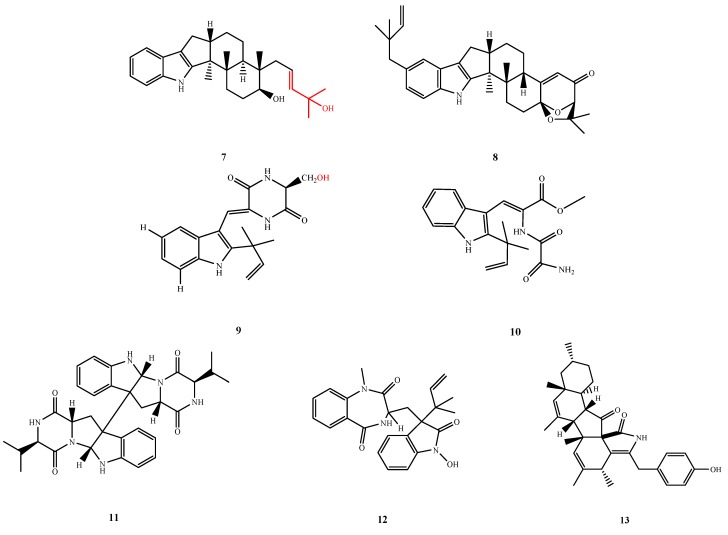
Structures of compound **7**–**13** (the red moiety enhances the antimicrobial activity).

**Figure 6:**
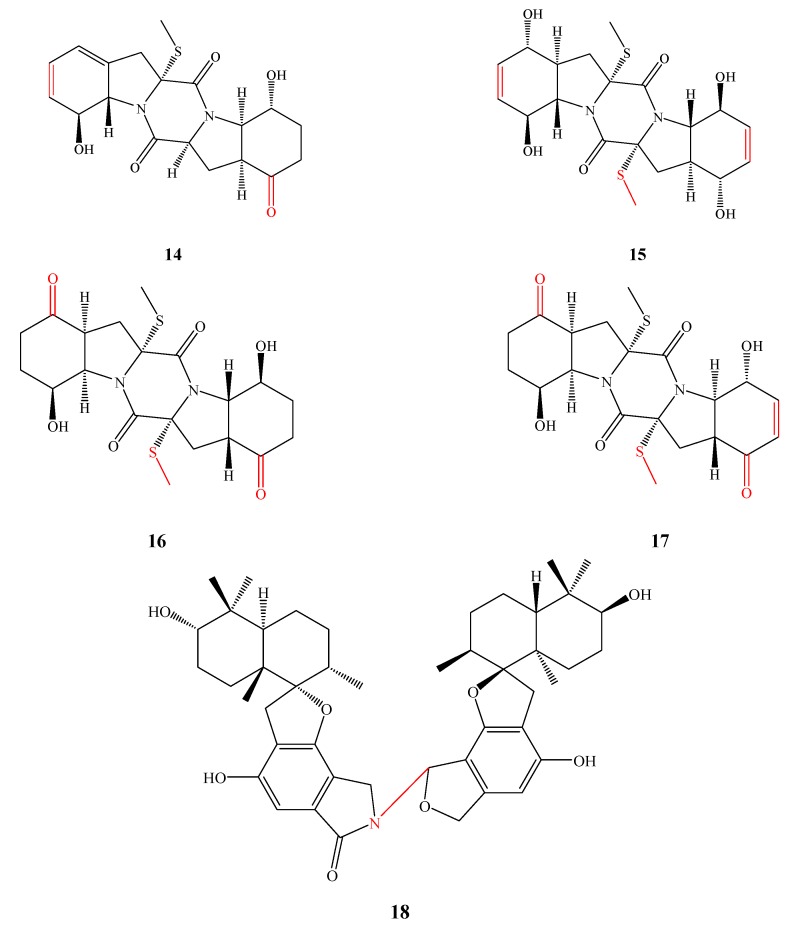
Structures of compound **14**–**18** (the red moiety enhances the antimicrobial activity).

Ten new indole-alkaloids, **7**–**12** and **14**–**17** (Figure 5 and Figure 6), showed antibacterial activities and most were isolated from marine fungi belonging to *Aspergillus* and *Penicillium* genera. The 4-hydroxy-4-methylpent-2-enyl moiety (red group shown in the chemical structure of **7**) in asporyzin C (**7**), which was isolated from *A. oryzae* was deduced to be necessary for antibacterial activity against *E. coli* [16]. Compound **8** was isolated from *A. flavus* OUCMDZ-2205, which exhibited stronger activity against *Staphylococcus aureus* (MIC, 20.5 μM) than did the known analog, β-aflatrem from the same fungal strain [17]. Cristatumins A (**9**), D (**10**) and E (**11**) were isolated from *Eurotium cristatum* EN-220 (**9** and **10**) and *E. herbariorum* HT-2 (*Eurotium* sp. is sexual state of *Aspergillus* sp.) [18,19]. These three compounds displayed bacterial inhibitory activity. Cristatumin A (**9**) exhibited activity against *E. coli* and *S. aureus* (MIC, 64 μg/mL) and cristatumin D (**10**) showed weak activity against *S. aureus* with an inhibition zone (IZ) of 8 mm at 100 μg/disk. Cristatumin E (**11**) exhibited antibacterial activity against *Enterobacter aerogenes* and *E. coli* (MIC, 44.0 and 44.0 μM, respectively). By comparison to known compound neoechinulin A, the antibacterial activity of **9** appears related to the hydroxyl moiety on C-20. Compound **12** was also isolated from an *Aspergillus* sp. and exhibited potent activity against *Vibrio* spp. (MIC, 0.1 and 1 μg/mL) [20]. Diaporthalasin (**13**), which was isolated from *Diaporthaceae* sp. PSU-SP2/4, displayed significant antibacterial activity against both *S. aureus* and methicillin-resistant *S. aureus* (MRSA) with equal MIC values of 2 μg/mL [21].

Penicibrocazines B–E (**14**–**17**) (Figure 6) were produced by *P. brocae* MA-231. Compounds **14**–**16** showed antibacterial activity against *S. aureus* (MIC, 32.0, 0.25 and 8.0 μg/mL, respectively) and compounds **14**, **16** and **17** showed antifungal activity against *Gaeumannomyces graminis* (MIC, 0.25, 8.0 and 0.25 μg/mL, respectively). By comparison with the known compound Penicibrocazine A lacking antimicrobial activity, the double bonds at C-6 and C-6′ increased activity against *S. aureus* and more *S*-methyl groups likely strengthened activity against *G. graminis*. In addition, the keto groups at C-5/5′ enhanced the activity against *G. graminis* [22]. Stachyin B (**18**) was isolated from *Stachybotrys* sp. MF347 and showed activity against three Gram-positive bacterial strains MRSA, *B. subtilis* and *S. epidermidis* (IC_50_, 1.75, 1.42 and 1.02 μM, respectively) but no activity against the Gram-negative test strain and the fungal strains. Stachyin B (**18**) is the first dimeric spirodihydrobenzofuranlactams with N–C linkage (red color shown in the below structure) and its analogs (the other dimeric spirodihydrobenzofuranlactams) are N–N linkage instead. This N–C linkage is important for its antibacterial activity, as determined by comparison of the activity of stachyin B (**18**) with other N–N connected analogs (other dimeric spirodihydrobenzofuranlactams) and stachyin A (a single spirodihydrobenzofuranlactam) [23].

#### 5.1.3. Pyridines and Pyridinones

**Figure 7:**
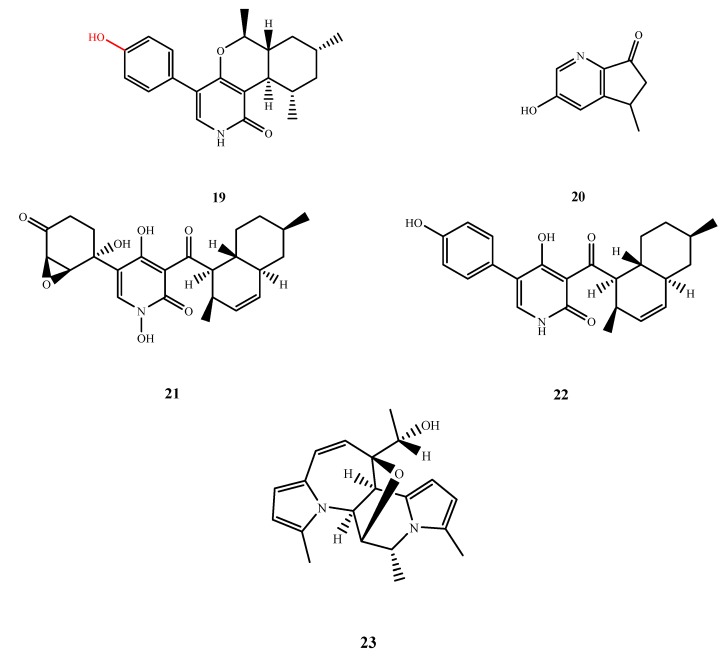
Structures of compound **19**–**23** (the red moiety enhances the antimicrobial activity).

The structures of compound **19**–**23** were shown in Figure 7. Trichodin A (**19**) was isolated from *Trichoderma* sp. MF106 and showed antibacterial activity against Gram-positive *B. subtilis* and *S. epidermidis* (IC_50_, 27.05 and 24.28 µM, respectively) and antifungal activity against *Candida albicans* (IC_50_, 25.38 μM) [24]. However, trichodin B, which substituted a ribofuranose group for the C-20 hydroxyl of **19**, showed no activity against any test microorganisms. Compound **20** was isolated from *Wallemia sebi* PXP-89 and was elucidated to be 5,6-dihydro-3-hydroxy-5-methylcyclopenta[b]pyridin-7-one with weak antibacterial activity against *E. aerogenes* (MIC, 76.7 µM) [25]. Didymellamides A (**21**) and B (**22**) were isolated from *Stagonosporopsis cucurbitacearum*. Didymellamide A (**21**) inhibited three strains of *Candida* spp. (MIC, 3.1 μg/mL) and *Cryptococcus neoformans* (MIC, 1.6 μg/mL). Didymellamide B (**22**) only inhibited *C. neoformans* (MIC, of 6.3 μg/mL) [26]. Curvulamine (**23**) was isolated from *Curvularia* sp. IFB-Z10 as a compound with a novel carbon skeleton and showed antibacterial activity against *Veillonella parvula*, *Streptococcus* sp. *Bacteroides vulgatus* and *Peptostreptococcus* sp. with an MIC value of 0.37 μM for all species. Curvulamine (**23**) has more selective antibacterial activity than tinidazole and is biosynthetically unique with a new extension formed through a decarboxylative condensation between an oligoketide motif and an alanine [27].

#### 5.1.4. Piperazine/Diketopiperazine and Pyrimidine/Pyrimidinone

**Figure 8:**
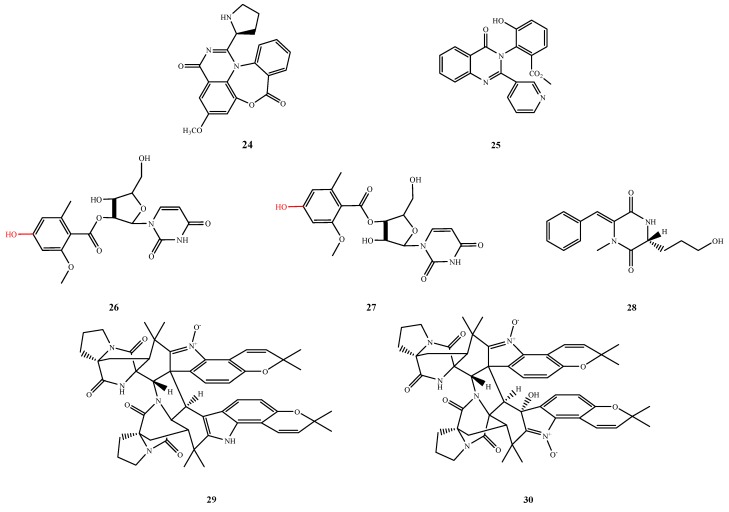
Structures of compound **24**–**30** (the red moiety enhances the antimicrobial activity).

The structures of compound **24**–**30** were shown in Figure 8. Aspergicin (**24**) was isolated from the co-culture of *Aspergillus* sp. FSY-01 and *Aspergillus* sp. FSW-02 and exhibited moderate antibacterial activity against *S. aureus* (MIC, 62.50 μg/mL), *S. epidermidis* (MIC, 31.25 μg/mL), *B. subtilis* (MIC, 15.62 μg/mL), *B. dysenteriae* (MIC, 15.62 μg/mL), *B. proteus* (MIC, 62.50 μg/mL) and *E.coli* (MIC, 31.25 MIC μg/mL) [28]. Terremides B (**25**), a pyrimidinone derivative, was isolated from *A. terreus* PT06-2 and showed weak activity against *E. aerogenes* (MIC, 33.5 μM) [29]. Two aroyl uridine derivatives kipukasins H (**26**) and I (**27**), were isolated from *A. versicolorstrain* ATCC 9577 and exhibited antibacterial activity against *S. epidermidis* (MIC, 12.5 μM). Their methylated derivatives (methoxyl substituted for C-4″ hydroxyl) were inactive against the test bacterial strain, which indicated that the hydroxyl at C-4″ may have a positive contribution to the antibacterial activity [30]. Pinodiketopiperazine A (**28**), a diketopiperazine derivative, was isolated from *P. pinophilum* SD-272 and displayed inhibitory activity against *E. coli* (IZ, 10 mm at 20 μg/disk) [31]. Two novel structural skeletons, compounds **29** and **30**, were isolated from an *Aspergillus* sp. after screening over two thousand fungal strains. Waikialoid A (**29**) and waikialoid B (**30**) demonstrated dose-dependent activity in the biofilm inhibition assay against *C. albicans* with IC_50_ values of 1.4 and 46.3 μM, respectively. Although waikialoid A (**29**) was unable to disrupt preformed biofilms, by microscopy studies, it inhibited cell adherence, hyphal development, and biofilm assemblies during the early stages of surface colonization, and it was not cytotoxic to human cells [32].

#### 5.1.5. Other N-Containing Compounds

**Figure 9:**
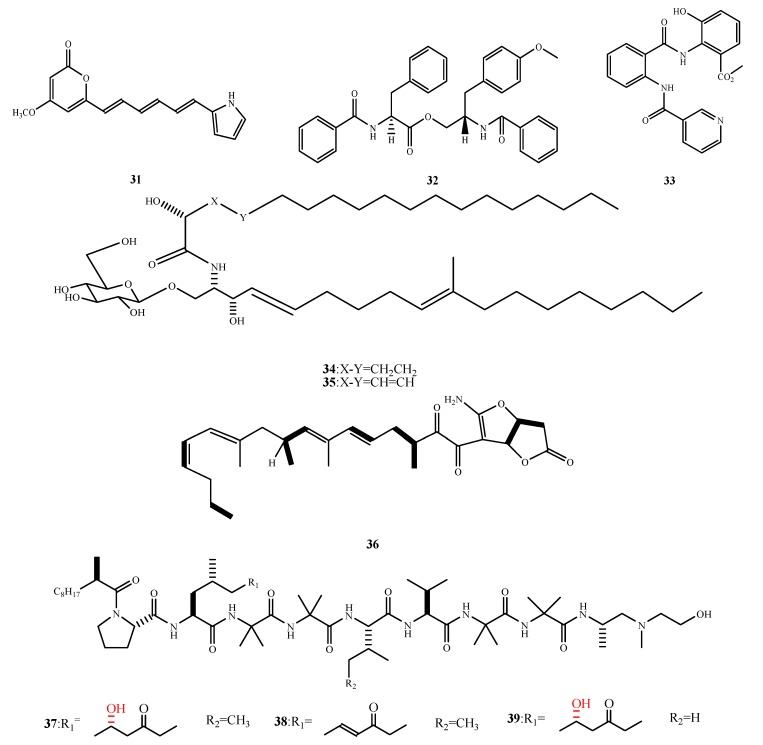
Structures of compound **31**–**39** (the red moiety enhances the antimicrobial activity).

The structures of compound **31**–**39** were shown in Figure 9. Compound **31** was only produced by *A. flocculosus* PT05-1 under high salt stress conditions and showed antibacterial activity against *E. aerogenes* (MIC, 3.7 μM) [33]. Compound **32** was elucidated to be 4′-methoxyl-asperphenamate and was isolated from *A. elegans* ZJ-2008010. It showed a similar activity as asperphenamate against *S. epidermis* (MIC, 10 μM) [34]. Terremide A (**33**) was isolated from *A. terreus* PT06-2 and showed weak activity against *S. aureus* (MIC, 63.9 μM) [29]. Two cerebrosides, flavuside A (**34**) and flavuside B (**35**), were isolated from *A. flavus*. They exhibited 15.6 and 31.2 μg/mL MICs for *S. aureus* and MRSA [35]. Compound **36** was isolated from *Paecilomyces* sp. and exhibited weak activity against MRSA [36]. Trichoderin A (**37**), trichoderin A1 (**38**) and trichoderin B (**39**) were isolated from fungal strain 05FI48 and they exhibited potent antibacterial activity against *M. smegmatis*, *M. bovis* BBG and *M. tuberculosis* H37Rv (MIC, 0.1, 0.02 and 0.12 μg/mL for **37**; 1.56, 0.16 and 2.0 μg/mL for **38**; 0.63, 0.02 and 0.13 μg/mL for **39**) [37,38]. Based on the comparison of the structures and activities of **37**–**39**, the R_1_ hydroxyls (the red moiety shown in the structure 37 and 39) of **37** and **39** are related to their antibacterial activity. The mechanism of **37** was investigated using a transformant of *M. smegmatis* with resistance to **37**. This *M. smegmatis* transformant over-expressed part of the genes that encoded the mycobacterial ATP synthase, indicating that the anti-mycobacterial activity of **37**–**39** is related to the inhibition of ATP synthesis.

### 5.2. Steroids and Terpenoid**s**

**Figure 10:**
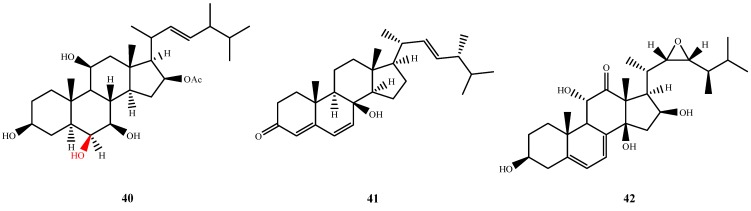
Structures of compound **40**–**42** (the red moiety enhances the antimicrobial activity).

Three new steroids with antimicrobial activity, **40**–**42** (Figure 10), were isolated from *P. chrysogenum* QEN-24S, *A. ustus* cf-42 and *A. flocculosus* PT05-1, respectively. Penicisteroid A (**40**) exhibited antifungal activity against *A. niger* and *Alternaria brassicae* (IZ, 24 mm and 16 at 20 μg/disk) [39]. The C-6 hydroxyl group may contribute to its activity, as determined by comparison with known compound anicequol (C-6 carbonyl group). Isocyathisterol (**41**) showed antibacterial activities against *E. coli* and *S. aureus* (IZ, 6.7 and 5.7 mm at 30 μg/disk, respectively) [40]. Compoud **42** was produced only under the high salinity conditions (10% salinity addition in fermentation) and exhibited antimicrobial activities against *S. aureus*, *E. coli*, and *A. niger* (MIC, 3.3, 3.3 and 1.6 μM, respectively) [33].

**Figure 11:**
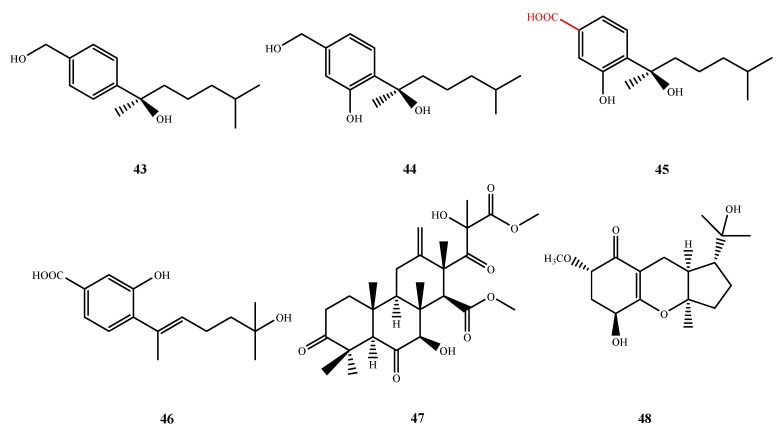
Structures of compound **43**–**48** (the red moiety enhances the antimicrobial activity).

Five new antimicrobial sesquiterpenoids, **43**–**47** (Figure 11), were isolated from *Aspergillus* sp. ZJ-2008004 (**43**–**45**), *Leucostoma persoonii* (**46**), *Aspergillus* sp. OPMF00272 (**47**) and *Scyphiphora hydrophyllacea* A1 (**44**). Aspergiterpenoid A (**43**), (−)-sydonol (**44**) and (−)-sydonic acid (**45**) showed activity against *E. coli* and *Micrococcus tetragenus* (MIC, 20 and 10 μM for **43**; 20 and 1.25 μM for **44**; and 5 and 20 μM for **45**) [41]. Furthermore, compound **45** showed activity against other four test bacteria *B. subtilis* (MIC, 2.5 μM), *Sarcina lutea* (MIC, 2.5 μM), *V. parahaemolyticus* (MIC, 10 μM) and *V. anguillarum* (MIC, 5 μM), that **39** and **40** were inactive against. The C-4 carboxyl of **45** was important for the activtiy. Engyodontiumone I (**46**) displayed antibacterial activity against *B. subtilis* (MIC, 256.0 μg/mL) [42]. Terretonin G (**47**), a new meroterpene, showed activity against Gram-positive bacteria *S. aureus*, *B. subtillis* and *M. luteus*(IZ, 4, 2 and 2 mm at 20 μg/disk, respectively), but not against the Gram-negative test bacteria and fungi [43]. Guignardones I (**48**) (Figure 11), another new meroterpene, exhibited antibacterial activity toward MRSA and *S. aureus* (IZ, 3 and 5 mm at 1 µg/disk, respectively) [44].

**Figure 12:**
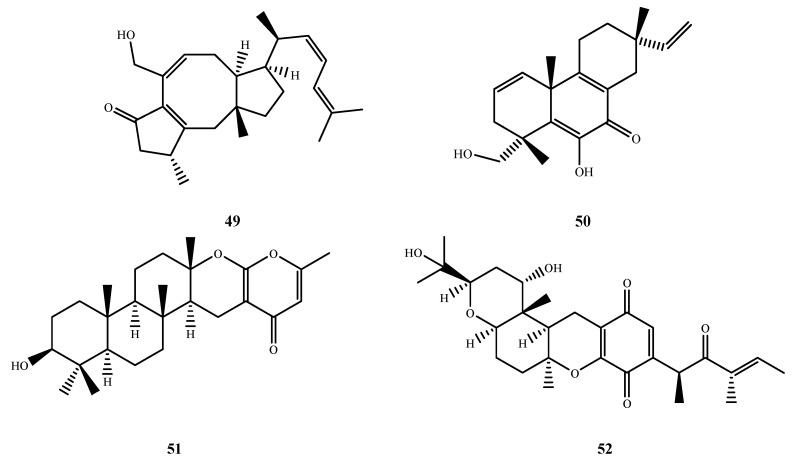
Structures of compound **49**–**52**.

The structures of compound **49**–**52** were shown in Figure 12. Sesterterpene ophiobolin U (**49**) was isolated from fresh *A. ustus* cf-42 and exhibited inhibitory activities against *E. coli* and *S. aureus* (IZ, 15 and 10 mm at 30 μg/disk, respectively) [45]. Libertellenone G (**50**) was isolated from *Eutypella* sp. D-1 and showed antibacterial activity against *E. coli*, *B. subtilis* and *S. aureus* (IZ, 8, 8 and 9 at 50 μg/disk) [46]. Chevalone E (**51**) was isolated from *A. similanensis* sp. nov. KUFA 0013, which was found to show synergism with the antibiotic oxacillin against methicillin-resistant *S. aureus* (MRSA) [47]. Compound **52** was only produced by *Nigrospora* sp. MA75 in medium with 3.5% NaI and exhibited activity against MRSA (MIC, 8 µg/mL), *E. coli* (MIC, 4 μg/mL), *P. aeruginosa* (MIC, 4 µg/mL), *P. fluorescens* (MIC, 0.5 µg/mL) and *S. epidermidis* (MIC, 0.5 µg/mL), which implies that the added iodide ion may be triggering the activation of a mixed polyketide terpenoid biosynthetic pathway in this fungal strain [48].

### 5.3. Polyketides

#### 5.3.1. Xanthones

**Figure 13:**
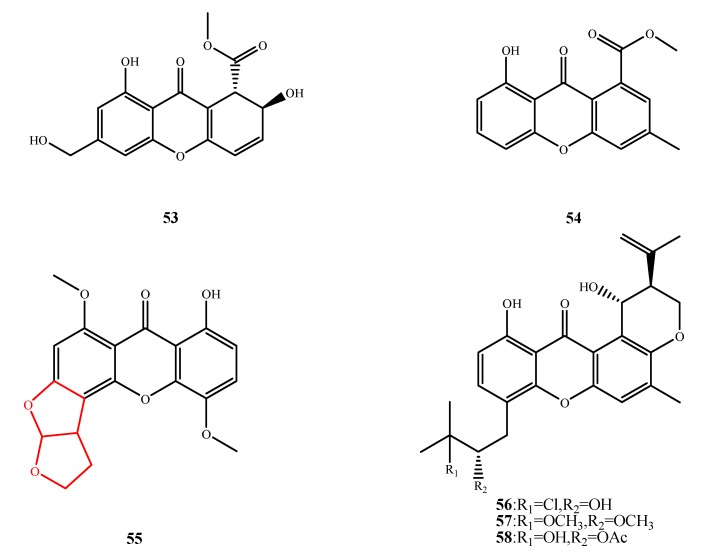
Structures of compound **53**–**58** (the red moiety enhances the antimicrobial activity).

The structures of compound **53**–**58** were shown in Figure 13. Six antimicrobial xanthones were isolated and their structures were elucidated. Engyodontiumone H (**53**) produced by *Engyodontium album* DFFSCS021 exhibited activity against *E. coli* (MIC, 64 µg/mL) and *B. subtilis* (MIC, 32 µg/mL) [42]. Compound **54** was isolated from the co-culture of unidentified strains E33 and K38 and exhibited activity against five flimantous fungal strains, *Gloeasporium musae*, *Blumeria graminearum*, *Fusarium oxysporum*, *Perononphthora cichoralearum* and *Colletotrichum glocosporioides* (respective inhibition rates of 53%, 4.6%, 9.5%, 48% and 28% at 100 µg/mL) [49,50]. Compound **55** was isolated from *A. versicolor* MF359 and showed significantly stronger activity against *S. aureus* (MIC, 12.5 μg/mL) and *B. subtilis* (MIC, 3.125 μg/mL) than **53** and **54** [51]. This difference implies that the two furan rings are most likely related to its antibacterial activity. Emerixanthones A (**56**), emerixanthone C (**57**) and emerixanthone D (**58**) isolated from *Emericella* sp. SCSIO 05240. Emerixanthones A (**56**) and emerixanthones C (**57**) exhibited antibacterial activity (IZ, 4–6 mm at 1.25 µg/disk) against all test bacteria *E. coli*, *Klebsiella pneumonia*, *S. aureus*, *Enterococcus faecalis*, *Acineto bacterbaumannii* and *Aeromonas hydrophila*. Emerixanthones D (**58**) showed an inhibitory zone of 3–4 mm against the fungal test phytopathogen *Fusarium* sp., *Penicillium* sp., *A. niger*, *Rhizoctonia solani*, *F. oxysporium* f. sp. *niveum* and *F. sporium* f. sp. *cucumeris* [52].

#### 5.3.2. Anthraquinones

**Figure 14:**
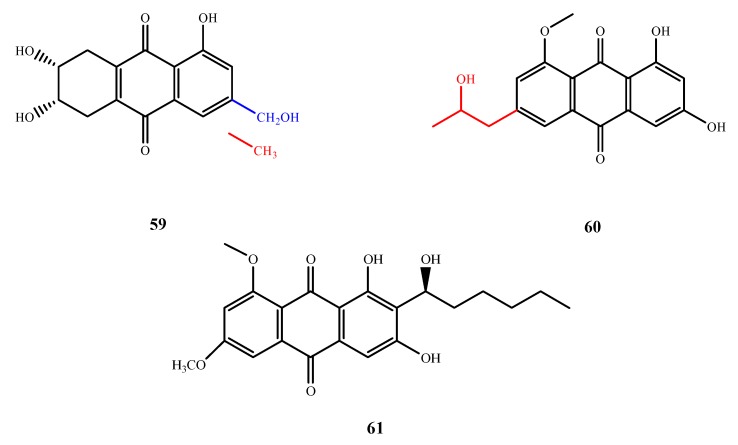
Structures of compound **59**–**61** (the red moiety enhances the antimicrobial activity).

The structures of compound **59**–**61** were shown in Figure 14. Trichodermaquinone (**59**) was isolated from *T. aureoviride* PSU-F95 and exhibited antibacterial activity against MRSA (MIC, 200 μg/mL). Compound **59** with a C-3 hydroxymethyl group (blue moiety in the structure of **59**) showed significantly weaker activity than coniothranthraquinone, a known compound produced by the same fungal strain with a C-3 methyl group (red moiety in the structure of **59**) (MIC, 8 μg/mL) [53]. Isorhodoptilometrin-1-methyl ether (**60**) was isolated from *A. versicolor* and exhibited antibacterial activity against three Gram-positive bacterial strains *B. cereus*, *B. subtilis* and *S. aureus* (IZ, 2, 3 and 5 mm at 50 µg/disk, respectively) [54]. The C-6 propanol group of **60** is important for its activity, as determined by comparison with inactive compound 1-methyl emodin. Compound **61** was isolated from *A. versicolor* EN-7 and exhibited antibacterial activity against *E. coli* (IZ, 7 mm at 20 μg/disk) [55].

#### 5.3.3. Quinones and Quinone Derivatives

**Figure 15:**
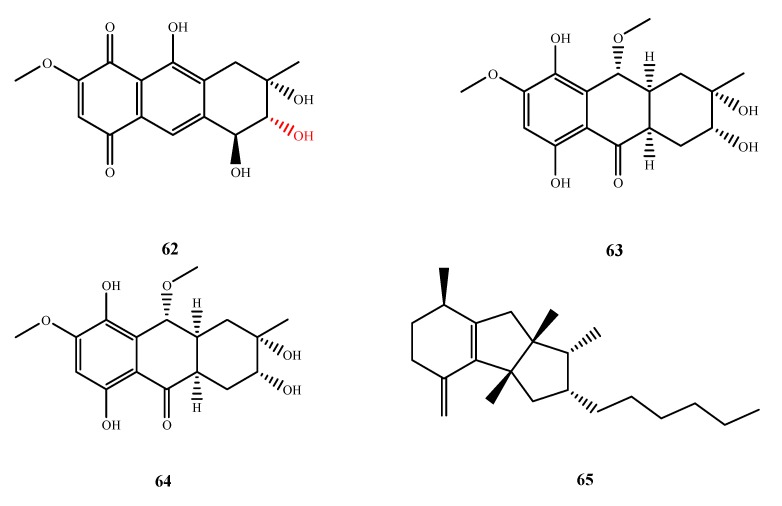
Structures of compound **62**–**65** (the red moiety enhances the antimicrobial activity).

The structures of compound **62**–**65** were shown in Figure 15. Anthraquinone derivatives, **62** and **63**, were isolated from *Nigrospora* sp. No. 1403. Compound **62** exhibited potent activity against *B. subtilis* (MIC, 0.625), *B. cereus* (MIC, 10.0), *M. luteus* (MIC, 20.0 µM), *S. albus* (MIC, 5.00 µM), *S. aureus* (MIC, 2.50 µM), *M. teragenus* (MIC, 1.25 µM), *E. coli* (MIC, 2.50 µM), *V. anguillarum* (MIC, 2.50 µM) and *V. parachemolyticus* (MIC, 1.25 µM). Compound **63** was from the same fungal strain, but its antibacterial activity was significantly weaker than **62** [56]. From comparison of several known anthraquinone derivatives, the hydroxyl groups of **62** at C-4 and C-9 have little effect on the antibacterial activity but the hydroxyl group of **62** at C-3 most likely contributes to its antibacterial activity [56,57]. Seimatorone (**64**) was isolated from *Seimatosporium* sp. No. 8883 and showed antibacterial activity against *E. coli* and *B. megaterium* (IZ, 3 and 7 mm at 50 μg/disk, respectively) [58]. Trichodermaketone A (**65**) was from *T. koningii* and exhibited synergistic antifungal activity against *C. albicans* at 125 µg/mL with 0.05 µg/mL ketoconazole [59].

**Figure 16:**
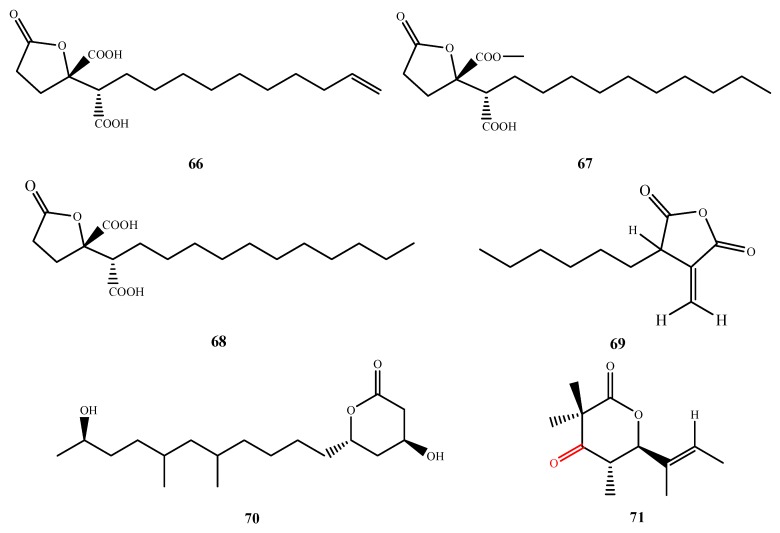
Structures of compound **66**–**71** (the red moiety enhances the antimicrobial activity).

The structures of compound **66**–**71** were shown in Figure 16. The structures of three new antimicrobial butenolides were elucidated from two *Aspergillus* spp. Spiculisporic acids B–D (**66**–**68**) were isolated from *Aspergillus* sp. HDf2 and exhibited a similar weak antibacterial activity against *S. aureus* [60]. Tubingenoic anhydride A (**69**), which was isolated from *A. tubingensis* OY907, inhibited *Neurospora crassa* growth (MIC, 330 μM) and affected hyphal morphology. Compound **69** may affect cell wall biosynthesis through a cytosolic protein that is the product of the new gene *mas-1*, originally characterized from the *N. crassa* mutant with tolerance to **69** [61]. Penicitide A (**70**) was purified from *P. chrysogenum* QEN-24S and displayed activity against *A. brassicae* (IZ, 6 mm at 20 μg/disk) [62]. Helicascolide C (**71**) was isolated from *Daldinia eschscholzii* KT32 and showed antifungal activity against phytopathogenic fungus *Cladosporium cucumerinu* (IZ, 5 mm at 200 μg/disk) [63]. By comparison to known compound helicascolide A, the C-3 keto group of **71** (the red moiety shown in the structure of 71) enhances the antifungal activity.

**Figure 17:**
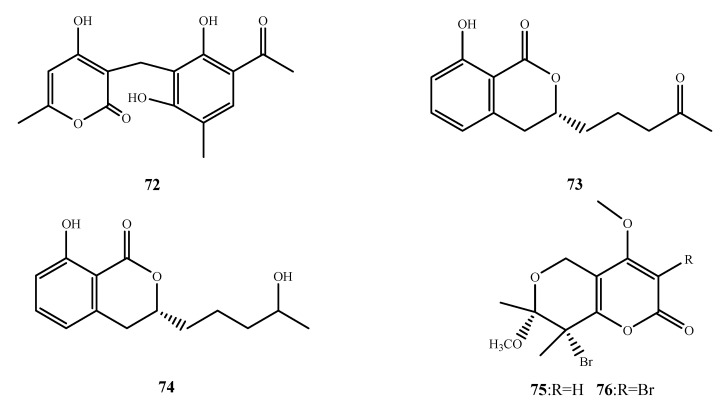
Structures of compound **72**–**76**.

The structures of compound **72**–**76** were shown in Figure 17. Communol A (**72**) was isolated from *P. commune* 518 and showed weak antibacterial activity against *E. coli* and *E. aerogenes* (MIC, 4.1 μM and 16.4 μM, respectively) [64]. Aspergillumarin A (**73**) and B (**74**) were isolated from *Aspergillus* sp. and exhibited weak activities against *S. aureus* and *B. subtilis* at 50 µg/mL [65]. Bromomethylchlamydosporols A (**75**) and B (**76**) were isolated from *Fusarium tricinctum.* The addition of CaBr_2_ to the fermentation media resulted in the production of two halogenated chlamydosporol analogs, **75** and **76**. Compounds **75** and **76** showed the same activity against three strains of *S. aureus* (MIC, 15.6 µg/mL) [66].

**Figure 18:**
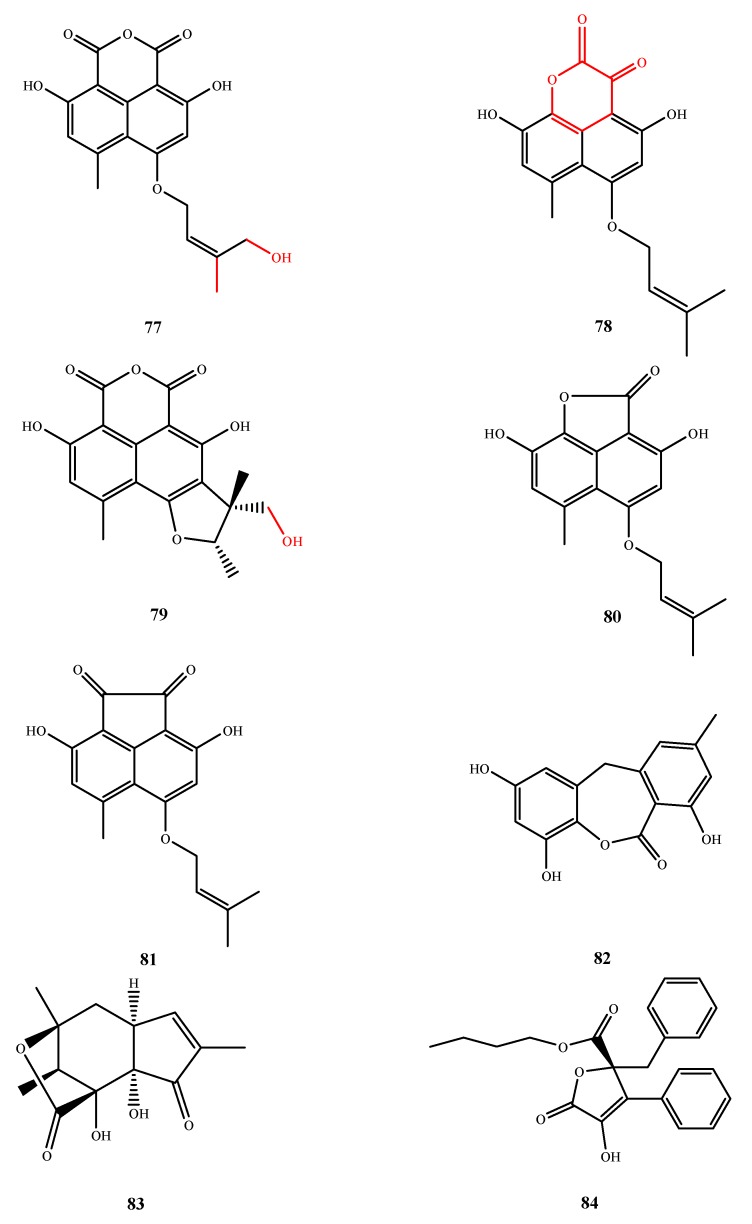
Structures of compound **77**–**84** (the red moiety enhances the antimicrobial activity).

The structures of compound **77**–**84** were shown in Figure 18. Three tricyclic lactones and one tetracyclic lactone with antimicrobial activities were isolated from *Coniothyrium cereale* (*Z*)-coniosclerodinol (**77**), conioscleroderolide (**78**), (15*S*,17*S*)-(−)-sclerodinol (**79**) and coniolactone(**80**), repectively. Moreover, coniosclerodione (**81**), with activity against *S. aureus* (MIC, 65.7 μM) as well as *Micrococcus luteus* and *Mycobacterium phlei* (IZ, 10 and 12 mm at 20 μg/disk, respectively),was isolated from the same fungal strain [67]. By comparison with its analogs, the activity against *M. phlei*. (IZ, 16 mm at 20 μg/disk) of **77** is related to its C-18 hydrogens and C-19 hydroxyl. Moreover, antibacterial activity seems to correlate with the presence of a diketo-lactone ring as found in compound **78**. The C-19 hydroxyl of **79** is also important for its activity against *M. phlei* (IZ, 20 mm at 20 μg/disk). Compound **82** was isolated from *E. rubrum* G2 and elucidated as 9-dehydroxyeurotinone with weak antibacterial activity against *E. coli* (IZ, 7 mm at 100 mg/disk) [68]. Acremostrictin (**83**) is another tricyclic lactone that was isolated from *A. strictum*,and it exhibited weak activity against *M. luteus*, *Salmonella typhimurium* and *Proteus vulgaris* (MIC, 50, 50 and 12.5 μg/mL, respectively) [69]. Flavipesin A (**84**) was isolated from *A. flavipes* AIL8 and demonstrated antibacterial activity against *S. aureus* (MIC, 8.0 μg/mL) and *B. subtillis* (0.25 μg/mL). Flavipesin A (**84**) also demonstrated activity against biofilm formation and could penetrate the mature biofilm matrix to kill the cell. Even penicillin cannot penetrate the polysaccharide barriers of a mature biofilm [70].

**Figure 19:**
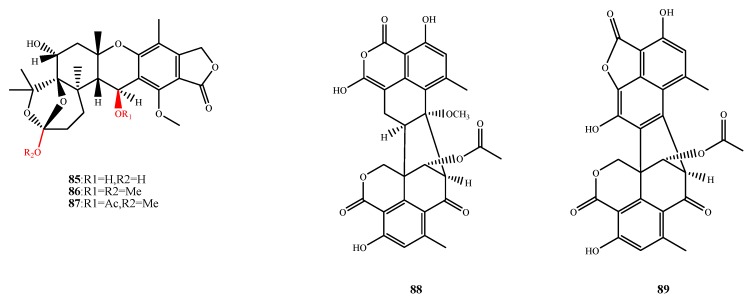
Structures of compound **85**–**89** (the red moiety enhances the antimicrobial activity).

The structures of compound **85**–**89** were shown in Figure 19. Austalide R (**85**), M (**86**) and N (**87**) were isolated from *Aspergillus* sp. and exhibited antibacterial activity against *Halomonas aquamarina*, *Polaribacter irgensii*, *Pseudoalteromonas elyakovii*, *Roseobacter litoralis*, *Shewanella putrefaciens*, *V. harveyi*, *V. natriegens*, *V. proteolyticus* and *V. carchariae* (MIC, 0.01–0.1 μg/mL for **85**; 0.001–0.01 μg/mL for **86** and 0.01 μg/mL for **87**). The R_1_ substituents at C-17 and R_2_ at C-22 (shown in the structures of **85** and **86**) significantly enhance their antibacterial activities [20,71]. Talaromycesone A (**88**) and B (**89**) were isolated from *Talaromyces* sp. LF458 and exhibited potent antibacterial activities against human pathogenic S. epidermidis (IC_50_, 3.70 and 17.36 μM, respectively) and *S. aureus* MRSA (IC_50_, 5.48 and 19.50 μM, respectively) [72].

**Figure 20:**
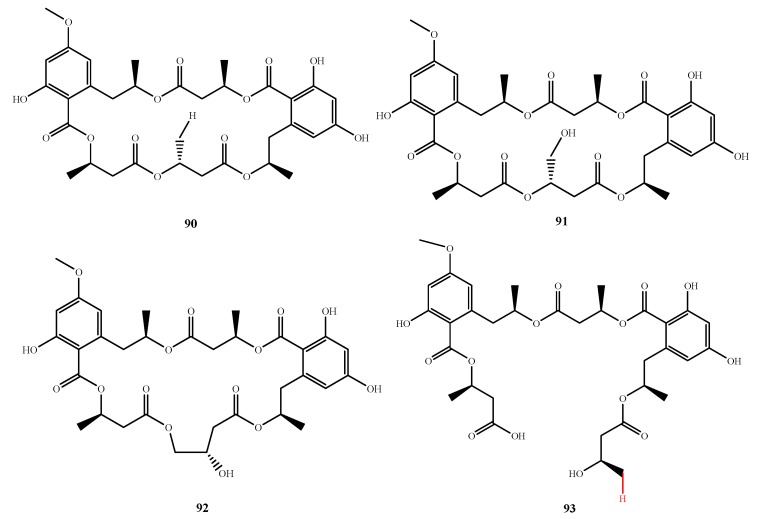
Structures of compound **90**–**93** (the red moiety enhances the antimicrobial activity).

Calcarides A–C (**90**–**92**) and E (**93**) (Figure 20) were isolated from *Calcarisporium* sp. KF525 and showed antibacterial activity against *S. epidermidis* and *X. campestris* (MIC, 68.8 and 5.5 μg/mL for **90**; 53.2 and 22.6 μg/mL for **91**; 29.6 and 61.4 μg/mL for **92**; and 104.3 and more than 150 μg/mL for **93**, respectively) [73]. In comparison to calcaride D that contains a hydroxyl, calcaride E (**93**) exhibited stronger activity with its hydrogen moiety. Three compounds, 6-hydroxymellein, β-hydroxybutyric acid and 6-methoxymellein, were proposed as the precursors of calcaride.

**Figure 21:**
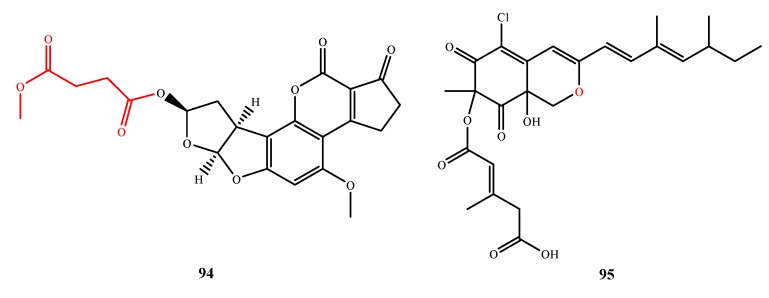
Structures of compound **94**–**95** (the red moiety enhances the antimicrobial activity).

Aflatoxin B_2b_ (**94**) (Figure 21) was isolated from *A. flavus* 092008 and exhibited moderate antimicrobial activity against *E. coli*, *B. subtilis* and *E. aerogenes* (MIC, 22.5, 1.7 and 1.1 µM, respectively) [74]. Compound **94** was elucidated to be the 4-methoxy-4-oxobutanoyl substitution for the acetyl group of 8-acetyloxyaflatoxin B_1_. By comparison to 8-acetyloxyaflatoxin B_1_, the 4-methoxy-4-oxobutanoyl moiety partly contributes to the antibacterial activity of **94**. Moreover, based on aflatoxins B_1_ and aflatoxins B_1_, the acetyloxy group of 8-acetyloxyaflatoxin B1 is not related to the antibacterial activity. Isochromophilone XI (**95**) (Figure 21) was isolated from *Bartalinia robillardoides* LF550 and showed antibacterial and antifungal activities against *B. subtilis*, *S. lentus* and *Trichophyton rubrum* (MIC, 55.6, 78.4 and 41.5 μM, respectively). The oxygen in the pyran ring of **95** is important for its antibacterial and antifungal activities, as determined by comparison to inactive analogs of **95** [75,76].

**Figure 22:**
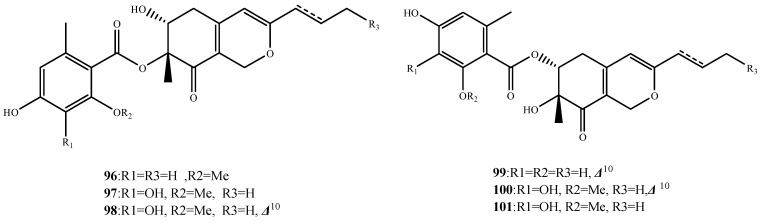
Structures of compound **96**–**101**.

Comazaphilones A–F (**96**–**101**) (Figure 22) were isolated from *P. commune* QSD-17. Comazaphilones C–E (**98**–**100**) displayed potent antibacterial activities against *S. aureus* MRSA, *P. fluorescens* and *B.subtilis* (MIC, 16, 64 and 32 μg/mL for **98**; 32, 16 and more than 256 μg/mL for **99**; and more than 256 μg/mL, 32 and 16 μg/mL for **100**, respectively). The SAR results indicated that the double bond at C-10 of **98**–**100**, as well as the location of the orsellinic acid unit at C-6 of **99** and **100** are important for their activity [77].

**Figure 23:**
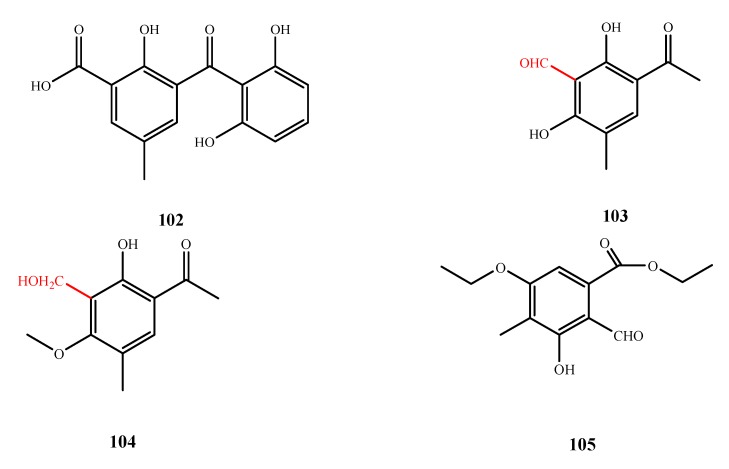
Structures of compound **102**–**105** (the red moiety enhances the antimicrobial activity).

The structures of compound **102**–**105** were shown in Figure 23. Isomonodictyphenone (**102**) was isolated *Penicillium* sp. MA-37 and showed potent antibacterial activity against *Aeromonas hydrophilia* (MIC, of 8 μg/mL) [78,79]. Communol F (**103**) and communol G (**104**) were isolated from *P. commune* 518 and showed weak antimicrobial activities against *E. coli* and *E. aerogenes* (MIC, 6.4 and 25.8 μM for **103**; and 23.8 and 23.8 μM for **104**, respectively) The CHO moiety of **105** and the CH_2_OH of **104** at C-3 are important for their activities, as determined by comparison to their inactive analogs [64]. Compound **105** was isolated from the co-culture of fungal strains E33 and K38 and exhibited antifungal activity against *F. graminearum*, *Gloeosporium musae*, *Rhizoctonia solani* and *Phytophthora sojae* (IZ, 12.1, 11.6, 10.2 and 8.5 mm at 0.25 mM, respectively) [49].

### 5.4. Others

**Figure 24:**
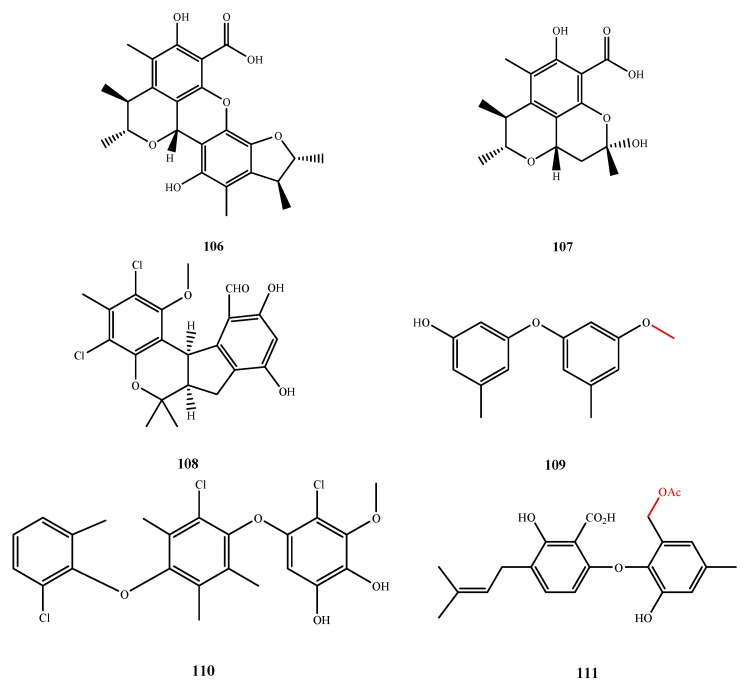
Structures of compound **106**–**111** (the red moiety enhances the antimicrobial activity).

The structures of compound **106**–**111** were shown in Figure 24. Penicitrinols J (**106**) and K (**107**) were isolated from *Penicillium* sp. ML226 and showed antimicrobial activity against *S. aureus* CMCC26003 (IZ, 4 and 3 mm at 20 μg/disk, respectively) [80]. Pestalachloride D (**108**) was isolated from *Pestalotiopsis* sp. (ZJ-2009-7-6) and showed antibacterial activity against *E. coli*, *V. anguillarum* and *V. parahaemolyticus* (MIC, 5.0, 10.0 and 20.0 μM, respectively) [81]. Cordyols E (**109**) was isolated from *Aspergillus* sp. XS-20090066 and showed antibacterial activity against all test bacteria *S. epidermidis*, *S. aureus*, *V. anguillarum*, *V. parahemolyticus* and *P. putida* (MIC, 25.6–51.2 μM). The methoxyl group of **109** at C-3 is related to its activity, as determined by comparison to the hydroxyl of diorcinol (a known antimicrobial compound) at C-3 [82]. Microsphaerol (**110**) was isolated from *Microsphaeropsis* sp. No. 7820 and showed antibacterial activity against *E. coli*, *B. megaterium* and *Microbotryum violaceum* (IZ, 8, 9 and 9 mm at 50 μg/disk, respectively) [58]. Compound **111** was isolated from *Penicillium* sp. MA-37 and elucidated as 7-*O*-acetylsecopeni-cillide C (**111**), which was active against *M. luteus* and *E. coli* (MIC, 64 and 16 μg/mL, respectively) [79]. The acetoxyl at C-7 of **111** is important to its antibacterial activity, which was determined by comparison of the hydroxyl at C-7 in the active secopenicillide C that is substituted by an acetoxyl group at C-7 in **111**.

**Figure 25:**
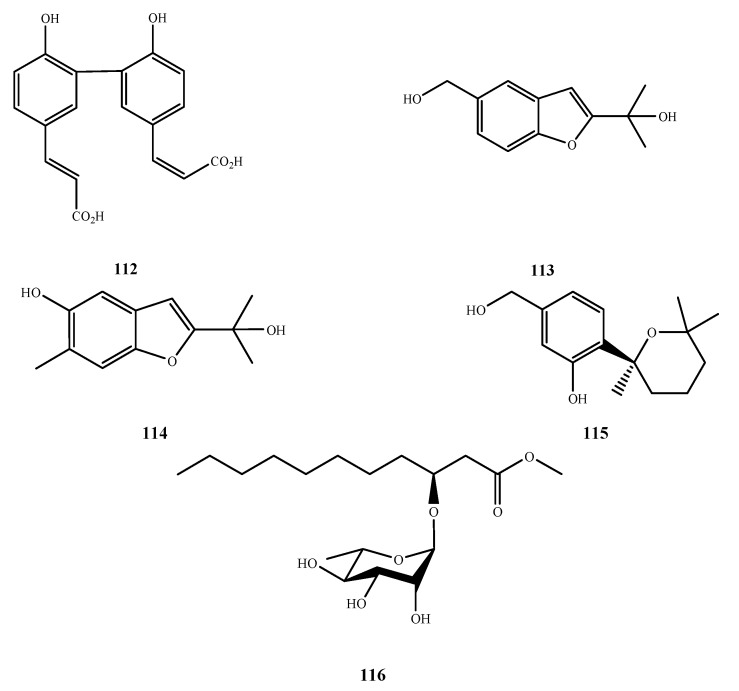
Structures of compound **112**–**116**.

The structures of compound **112**–**116** were shown in Figure 25. Compound **112** was isolated from *Spicaria elegans* KLA-03 and showed antibacterial and antifungal activities against *E. aerogenes*, *E. coli*., *P. aeruginosa*, *S. aureus* and *C. albicans* (MIC, 0.15, 0.04, 0.77, 1.53 and 0.38 μM, respectively) [83]. Felinone B (**113**) was isolated from *B. felina* EN-135 and it showed inhibitory activity against *P. aeruginosa* (MIC, 32 μg/mL) [84]. Isoacremine D (**114**), an isomer of **113**, was isolated from *Myceliophthora lutea* and exhibited antibacterial activity against *S. aureus* (MIC, 200 μg/mL) [85]. Compound **115** was isolated from *Aspergillus* sp. ZJ-2008004 and exhibited two Gram-positive bacteria, *S. albus* and *B. subtilis* (MIC, 5 and 2.5 μM, respectively) [41]. New fatty acid glycoside **116** was isolated from *Scyphiphora hydrophyllacea* A1 and showed inhibitory effects on *S. aureus* and MRSA (IZ, 3.8 and 4.7 at 500 μg/disk, respectively) [86].

## 6. Known Antibacterial and Antifungal Compounds from Marine Fungi

As shown in Table 1, there are 169 known compounds **117**–**285** from marine fungi showing antibacterial or antifungal activities (structures are shown in supplement materials Figure S2). Compounds **142**, **187**, **230**, **255**, **263**, **271** and **272** were isolated for the first time from natural sources [31,87,88,89]. Compounds **128**, **168**, **169**, **187**, **203**, **222**, **234**, **235**, **241** and **267** were evaluated for their antimicrobial activities for the first time [15,19,90,91,92,93]. The data shown in Table 1 would be useful for the utilization of these antibacterial and antifungal compounds from marine fungi as lead compounds for future medicine.

**Table 1:** Known compounds **117**–**285** with antibacterial or antifungal activities isolated from marine fungi.

Compound name	Activity	Reference	Compound name	Activity	Reference
(−)-scleroderolide (**117**)	B+, Y	[67]	(−)-sclerodione (**118**)	B+, Y	[67]
(−)-sclerotiorin (**119**)	Y, F	[94]	(−)-stephacidin A (**120**)	B+	[82]
(±)-pestalachloride C (**121**)	B−	[81]	(5α,6α)-ophiobolin H (**122**)	B−	[45]
15G256β (**123**)	B	[73]	15G256α (**124**)	B	[73]
15G256π (**125**)	B+	[73]	1-methyl emodin (**126**)	B	[54]
2,5-furandimethanol (**127**)	B+	[36]	3-HPA (**128**)	B+	[90]
4-deoxybostrycin (**129**)	B	[57]	4-hydroxybenzaldehyde (**130**)	B−	[95]
6,8-di-*O*-methylaverufin (**131**)	B	[96]	6-*epi*-ophiobolin G (**132**)	B+	[97]
6-*epi*-ophiobolin K (**133**)	B+	[97]	6-*O*-methylaverufin (**134**)	B	[96]
7-nor-ergosterolide (**135**)	Y, B	[33]	8-acetyloxyaflatoxin B1 (**136**)	B−	[74]
acetylgliotoxin (**137**)	B+	[87]	adenosine (**138**)	B−	[98]
aflatoxins B_1_ (**149**)	B−	[74]	aflatoxins B_2_ (**140**)	B−	[74]
AGI-B4 (**141**)	B	[42]	alternariol 2,4-dimethyl ether (**142**)	B−	[31]
anicequol (**143**)	F	[39]	AS-186c (**144**)	B+	[72]
aspergillazine A (**145**)	B, Y	[83]	aspergillin PZ (**146**)	B+	[34]
aspergillusene A (**147**)	B−	[99]	aspergillusidone C (**148**)	B+	[100]
aspergillusone B (**159**)	B	[42]	asperphenamate (**150**)	B+	[34]
aspochalasin E (**151**)	B, Y	[83]	aspochalasin D (**152**)	B	[34]
aspochalasin I (**153**)	B+	[34]	aspulvinone E (**154**)	B, Y	[83]
averantin (**155**)	B+	[101]	averufin (**156**)	B+	[101]
bostrycin (**157**)	B	[56]	brefeldin A (**158**)	Y	[102]
brevianamide M (**159**)	B	[96]	butyrolactone I (**160**)	B+	[88]
chlamydosporol (**161**)	B+	[66]	cholesteryl linoleate (**162**)	B+	[36]
chrysazin (**163**)	Y	[103]	*cis*-cyclo(Leu-Tyr) (**164**)	B+	[104]
citrinin (**165**)	B	[105]	CJ-17665 (**166**)	Y	[32]
cladosporin (**167**)	B+	[106]	conidiogenol (**168**)	B	[91]
conidiogenones B (**169**)	B, Y	[91]	coniothranthraquinone 1 (**170**)	B+	[53]
cordyol C (**171**)	B	[82]	cpicpoformin (**172**)	B+	[106]
cyclo(d)-Pro-(d)-Val (**173**)	B−	[31]	cyclosporine A (**174**)	Y, F	[107]
cytochalasin Z17 (**175**)	B−	[20]	cytosporone B (**176**)	B+	[108]
cytosporone E (**177**)	B+	[108]	deacetylsclerotiorin (**178**)	B+, Y	[75,76]
dechlorogriseofulvin (**179**)	Y	[48]	dicerandrol C (**180**)	B+	[109]
dihydroisoflavipucine (**181**)	B	[20,71]	diorcinol (**182**)	B+	[99,110]
djalonensone (**183**)	B−	[111]	echinulin (**184**)	B+	[19]
epolones B (**185**)	Y	[102]	ergone (**186**)	B+,Y	[112]
eurorubrin (**187**)	B−	[92]	fonsecin (**188**)	F	[113]
fumitremorgin B (**189**)	B−	[114]	furandimethanol (**190**)	B+	[98]
fusaric acid (**191**)	B+	[115]	fusarielin A (**192**)	B+	[66]
gliotoxin (**193**)	B	[87]	globosuxanthone A (**194**)	Y	[103]
glyantrypine (**195**)	B−	[114]	griseofulvin (**196**)	Y	[48]
griseophenone C (**197**)	B	[48]	guignardones B (**198**)	B+	[44]
helicusin A (**199**)	Y	[76]	hydroxysydonic acid (**200**)	B	[42]
stachybocin A (**201**)	B+	[23]	ilicicolin B (**202**)	B+	[23]
isaridin E (**203**)	B−	[84]	isochaetochromin B2 (**204**)	B+	[116]
isorhodoptilometrin (**205**)	B+	[53]	lapatins B (**206**)	B−	[114]
malformins A1 (**207**)	B+	[117]	malformins C (**208**)	B+	[117]
meleagrin (**209**)	B+, Y	[95]	methylaverantin (**210**)	B+	[101]
*N*-acetyldopamine (**211**)	F	[95]	neoaspergillic acid (**212**)	B	[118]
nidulin (**213**)	B+	[100]	nidurufin (**214**)	B+	[101]
nigrosporin B (**215**)	B	[119]	nornidulin (**216**)	B+	[100]
notoamide B (**217**)	Y	[32]	notoamide R (**218**)	Y	[32]
ophiobolin K (**219**)	B+	[97]	oxasetin (**220**)	B−	[120]
patulin (**221**)	B+	[106]	penicillixanthone A (**222**)	B	[93]
pestalone (**223**)	B+, F	[121]	phomaligol A (**224**)	B+	[35]
phomazine B (**225**)	F	[22]	phyllostine (**226**)	B+	[106]
pycnidione (**227**)	Y	[102]	pyridoxatin (**228**)	B+, Y	[24]
pyrophen (**229**)	Y	[113]	reduced gliotoxin (**230**)	B	[87]
rubralide C (**231**)	B−	[31]	rubrofusarin B (**232**)	Y	[113]
sclerotiamide (**233**)	Y	[32]	secalonic acid B (**234**)	B	[93]
secalonic acid D (**235**)	B	[93]	siderin (**236**)	B	[54]
sporogen AO-1 (**237**)	Y	[122]	stachybocin B (**238**)	B+	[23]
stephacidin A (**239**)	Y	[32]	stigmasterol (**240**)	B+	[36]
tardioxopiperazine A (**241**)	B	[19]	tetrahydrobostrycin (**242**)	B	[48]
trichodermamide B (**243**)	B, Y	[83]	trichodermamides A (**244**)	B, Y	[83]
tyrosol (**245**)	B+	[98]	ustilaginoidin D (**246**)	B+	[116]
verruculogen (**247**)	B−	[114]	waikialides A (**248**)	Y	[32]
waikialides B (**249**)	Y	[32]	xanthocillin X (**250**)	B, F	[95]
**Compound name**				**Activity**	**Reference**
ω-hydroxyemodin (**251**)				B+	[53]
(3β,5α,8α,22*E*)-5,8-epidioxyergosta-6,9,22-trien-3-ol (**252**)				B+	[112]
(−)-7,8-dihydro-3,6-dihydroxy-1,7,7,8-tetramethyl-5*H*-furo-[2¢,3¢:5,6]naphtho[1,8-*bc*]furan-5-one (8) (**253**)				B+	[67]
(*Z*)-5-(hydroxymenthyl)-2-(60)-methylhept-2′-en-2′-yl)-phenol (**254**)				B−	[41,99]
1,2,3,4-tetrahydro-2-methyl-3-methylene-1,4-dioxopyrazino[1,2-α]indole (**255**)				B+	[87]
1,3,8-trihydroxy-6-methylanthracene-9,10-dione (**256**)				B	[53,54]
2-(hydroxymethyl)benzene-1,4-diol (**257**)				B+	[89]
2-carboxymethyl-3-hexylmaleic acid anhydride (**258**)				F	[61]
2-methylbenzene-1,4-diol (**259**)				B+	[89]
3-(3-hydroxy-5-methylphenoxy)-5-methylphenol (**260**)				B	[82]
3,1′-didehydro-3[2″(3‴,3‴-dimethyl-prop-2-enyl)-3″-indolylmethylene]-6-methyl pipera-zine-2,5-dione (**261**)				B−	[123]
3,6,8-trihydroxy-1-methylxanthone (**262**)				B	[48]
3,9-dimethyldibenzo[*b*,*d*]furan-1,7-diol (**263**)				B+	[88]
3b-hydroxyergosta-8,24(28)-dien-7-one (**264**)				B+	[33]
3-hydroxy-4-((*S*)-2-hydroxy-6-methylheptan-2-yl)benzoic acid (**265**)				B−	[99]
3-hydroxy-5-methyl-5,6-dihydro-7*H*-cyclopenta[*b*]pyridin-7-one (**266**)				B+	[124]
3-*O*-(a-d-ribofuranosyl)questin (**267**)				B−	[92]
3β,5α-dihydroxy-(22*E*,24*R*)-ergosta-7,22-dien-6β-yl oleate (**268**)				B+	[112]
4-deoxytetrahydrobostrycin (**269**)				B−	[48]
4-methoxycarbonyldiorcinol (**270**)				B	[82]
4-*O*-methyltoluhydroquinone toluhydroquinone (**271**)				B+	[89]
5-bromotoluhydroquinone toluhydroquinone (**272**)				B+	[89]
6,8-di-*O*-methylnidurufin (**273**)				B	[55]
6,8-di-*O*-methylversiconol (**274**)				B−	[55]
6-[2-hydroxy-6-(hydroxymethyl)-4-methylphenoxy]-2-methoxy-3-(1-methoxy-3-methylbutyl)benzoic acid (**275**)				B	[78,79]
9α-hydroxydihydrodesoxybostrycin (**276**)				B	[56]
8-*O*-4-dehydrodiferulic acid (**277**)				B−	[20,71]
9α-hydroxyhalorosellinia A (**278**)				B	[56]
cyclo-*trans*-4-OH-(d)-Pro-(d)-Phe (**279**)				B−	[31]
methyl 3,4,5-trimethoxy-2-(2-(nicotinamido) benzamido) benzoate (**280**)				B+	[29]
*N*-methylphenyldehydroalanyl-l-prolin-anhydrid (**281**)				B−	[31]
*O*-methyldihydrobotrydial (**282**)				B+	[21]
stigmasta-7,22-diene-3β,5α,6α-triol (**283**)				B+	[112]
tetranorditerpenoid derivative (**284**)				Y	[125]
tricycloalternarene 3α (**285**)				B−	[111]

Activities: B+ means against Gram-positive bacteria; B− means against Gram-negative bacteria; B means against both Gram-positive and Gram-negative bacteria; Y means against yeast; and F means against filamentous fungi.

## 7. Conclusions

The resource of marine fungal species is abundant and the cultivable marine fungal strains are easily isolated and cultured. However, it is difficult to discover the marine fungi with special metabolites. To facilitate the discovery process, there are several methods to screen the marine fungi before further purification of their antimicrobial compounds. The taxonomic information, the screening of antimicrobial activity for the extracts from marine fungi, the analysis of the genes related to secondary metabolism and the comparison of the chemical profiles with the literature can contribute to the screening of special fungal strains. ITS sequence analysis cannot only afford the taxonomic information, but also facilitates investigation and acquisition of the closest fungal strain by their accession numbers. Therefore, obtaining the ITS sequence is recommended for every study. It can become the bridge between biology and chemistry. Another molecular biological method, analysis of the genes related to secondary metabolism, could be used for screening the marine fungi producing new antibacterial and antifungal compounds. Furthermore, there is previous research investigating the diversity of type I polyketide synthase (PKS I) genes from many marine fungi to find potential fungi producing new antibacterial and antifungal compounds [112].

The fermentation conditions of the potential fungal strain significantly affect the secondary metabolism of marine fungi. Some of the new antimicrobial compounds from marine fungi are only produced under high salinity [33,48,66]. The fungal strains that new antimicrobial compounds have been isolated from are recommended for investigation of their metabolic changes under different fermentation processes (especially under high salinity). The potential of these fungi have been proven, thus it is possible that more new antimicrobial compounds will be discovered under different fermentation conditions.

The *Aspergillus* genus is one of the dominant marine fungal genera and the marine fungal strains from *Aspergillus* produced more new antibacterial and antifungal compounds than any other genus. Furthermore, EtOAc is the most common solvent for the extraction of marine fungal cultures, which can also extract abundant compounds from mycelia or liquid culture, especially compounds with low or medium polarity. It is one of the reasons that water-soluble compounds (polar compounds) with antibacterial or antifungal activities from marine fungi are fewer than those from actinomyces and bacteria. For the antibacterial or antifungal tests of the compounds from marine fungi, *S. aureus*, *B. subtilis*, *E. coli* and *C. albicans* were recommended as the test microorganisms, and commercial antibiotics were used as positive controls, which is a convenient comparison for the compounds from different marine fungi. Importantly, the stereochemical configurations of the marine fungal compounds affect their antibacterial or antifungal activities. Thus, the stereochemical configurations of the pure compounds should be elucidated and evaluated for their activity mechanisms. In addition, typically too few compounds with similar structures for a structure-activity relationship can be purified from marine fungi; therefore, the total synthesis or a group derivation of compounds from marine fungi may help solve this problem. The bacterial or fungal mutant with a resistant gene to the antibacterial or antifungal compounds, morphological microscopic observation of the test microorganisms and RNA sequencing are recommended to contribute to understanding the mechanism of antibacterial or antifungal activities. This information will be beneficial for further utilization and development of antibacterial and antifungal compounds from marine fungi.

Taken together, these data indicate that marine fungi are a good new antibacterial and antifungal compound source. Many novel antibacterial and antifungal compounds that are only produced by these marine fungi have been discovered. There will certainly be more antibacterial and antifungal compounds from marine fungi as lead compounds for medicines and pesticides in the future.

## Supplementary Files

Supplementary File 1
