# Supplementary material for: Antibacterial and Antifungal Compounds from Marine Fungi
Source: Mar Drugs. 2015 Jun 2;13(6):3479–513. doi: 10.3390/md13063479 (PMC4483641; doi:10.3390/md13063479)
Supplement: Supplementary File 1 [file marinedrugs-13-03479-s001.pdf]

Supplementary Information

Table S1. Collecting locations, sources and names of marine fungi.

Site	Location	Fungal name	Source	Ref.
1	South China Sea	*Engyodontium album* DFFSCS021	sediment	[42]
2	Sedot-Yam, Israel	*Aspergillus tubingensis* OY907	sponge	[61]
3	the South China Sea	*Emericella* sp. SCSIO 05240	sediment	[52]
4	Cicia, Lau group, Fiji Islands	*Penicillium* sp. FF001	water	[105]
5	Adriatic Sea nearby Rovinj (Istrian Peninsula, Croatia)	*Bartalinia robillardoides* LF550	Water	[75,76]
6	a reef wall, Fijiislands	ascomycete Super 1F1-09	sponge	[87]
7	Praia Dura, Ubatuba vicinity, Sao Paulo state, Brazil	*Phomopsis longicolla*	alga	[109]
8	Punta di Fetovaia, Isle of Elba	*Talaromyces* sp. LF458	sponge	[72]
9	South China Sea	193 culturable fungal	animals	[10]
10	Playa del Ingles (Gomera, Spain)	*Microsphaeropsis* sp.<br>*Seimatosporium* sp.	other plant	[58]
11	Adriatic Sea	*Aspergillus* sp.	sponge	[20,71]
12	Greenland Sea	*Trichoderma* sp. MF106	water	[24]
13	Helgoland	*Stachybotrys* sp. MF347	driftwood	[23]
14	Dafeng harbor in the city of Yancheng, Jiangsu Province, China.	*Penicillium citrinum Salicorn* 46	other plant	[112]
15	the Xisha Islands of China	*Aspergillus sydowii* ZSDS1-F6	sponge	[99]
16	the Lianyungang sea area, Jiangsu Province of China	*Aspergillus flavus* OUCMDZ-2205	animals	[17]
17	the Bohai Sea, China	*Aspergillus versicolor* MF359	sponge	[51]
18	the Similan Islands, Phang Nga Province, Thailand	*Aspergillus similanensis* sp. nov. KUFA 0013	sponge	[47]
19	Prince Edward Island	*Neosetophoma samarorum* RKDO834<br>fungus RKDO795<br>*Phaeospheria spartinae* RKDO785	water	[102]
20	Hainan Island, P. R. China	*Penicillium* sp. MA-37	mangrove	[78,79]
21	Wenchang, Hainan, China	*Penicillium adametzioides* AS-53	sponge	[114]
22	Hainan Sanya National Coral Reef Reserve, China	*Neosartorya pseudofischeri*	animals	[86]
23	Trang province, Thailand	*Diaporthaceae* sp. PSU-SP2/4	sponge	[21]
24	Leizhou Peninsula, Guangdong Province, China	*Phomopsis* sp. K38<br>*Alternaria* sp. E33	mangrove	[13]
25	the shore of Bridge End, Shetlan Islands, UK	*Penicillium* sp. AF3-117C	alga	[106]
26	Okinawa, Japan	*Aspergillus* sp. OPMF00272	animals	[43]
27	the coast of Qingdao, P. R. China	*Eurotium cristatum* EN-220	alga	[91]
28	Daya Bay, Shenzhen City, Guangdong Province	*Aspergillus flavipes* AIL8	mangrove	[70]
29	Putian saltern, Fujian Province of China.	*Aspergillus flocculosus* PT05-1	sediment	[33]
30	Weizhou coral reef in the South China Sea	*Aspergillus elegans* ZJ-2008010	coral	[34]
31	the South China Sea	*Xylariaceae* sp. SCSGAF0086	coral	[123]
32	Yongxing Island in the South China Sea	*Pestalotiopsis* sp. ZJ-2009-7-6	coral	[81]
33	Taiwan Strait, China	*Penicillium* sp. ML226	mangrove	[80]
34	Pearl River in South China Sea	*Penicillium pinophilum* SD-272	sediment	[31]
35	the South China Sea	*Nigrospora* sp. No. 1403	mangrove	[56,57,119]
36	the German Wadden Sea	*Calcarisporium* sp. KF525	water	[73]
37	Arvoredo Island in the Arvoredo Biological Marine Reserve	*Penicillium* sp. F37	sponge	[104]
38	Zhoushan Island	*Aspergillus ustus* cf-42	alga	[45]
39	Qingdao's first beach, Shandong province of China	*Eurotium herbariorum* HT-2	alga	[18]

Table S1. *Cont.*

40	Naozhou Isoland, Guangxi Province, China	*Metarhizium anisopliae* mxh-99	sponge	[116]
41	Gokasyo Gulf, Mie Prefecture, Japan	*Emericella variecolor* GF10	sediment	[97]
42	Qingdao coastline of Shandong Province	*Aspergillus versicolor* EN-7	alga	[55]
43	Hainan Island, China	121 fungal isolates	coral	[7]
44	Wenchang county in Hainan Province	endophytic fungus A1	mangrove	[85]
45	Hainan Island, China	*Eurotium rubrum* G2	mangrove	[68]
46	Iriomote Island in Okinawa, Japan	*Beauveria bassiana* TPU942	sponge	[103]
47	Qionghai, Hainan, China	*Aspergillus* sp. HDf2	animals	[60]
48	Danzhou, Hainan Province of China	*Penicillium commune* 518	coral	[64]
49	Wenchang, Hainan province of China	*Aspergillus flavus* 092008	mangrove	[74]
50	South Sulawesi, Indonesia	*Daldinia eschscholzii* KT32	alga	[63]
51	Guangxi Zhuang Autonomous Region of China	*Nigrospora* sp. MA75	mangrove	[48]
52	Red Sea at Safaga coasts, Egypt	*Alternaria alternata*D2006	coral	[113]
53	Bertioga	*Diaporthe phaseolorum* 41.1	mangrove	[89]
54	the state of Sao Paulo	*Diaporthe phaseolorum* 41.1	mangrove	[89]
55	Pingtan Island, China	*Aspergillus versicolor*	alga	[96]
56	Wenchang County in Hainan Province (China)	*Scyphiphora hydrophyllacea* A1	mangrove	[44]
57	Weizhou coral reef in the South China Sea	*Aspergillus* sp. ZJ-2008004	sponge	[41]
58	Similan Islands, Phangnga Province, Thailand	*Trichoderma aureoviride* PSU-F95	coral	[53]
59	south Sinai, Egypt	*Aspergillus versicolor*	alga	[54]
60	La Jolla shore, San Diego, USA	*Asteromyces cruciatus* 763	alga	[14]
61	Qingdao	*Eurotium cristatum* EN-220	alga	[19]
62	Chorao Island, along the Mandovi estuary of Goa, India	*Penicillium chrysogenum* MTCC 5108	mangrove	[122]
63	Florida, US	*Leucostoma persoonii*	mangrove	[108]
64	Hainan, China	*Aspergillus niger* MA-132	mangrove	[117]
65	Putian Saltern of Fujian Province of China	*Aspergillus terreus* PT06-2	sediment	[29]
66	Weizhou Island of southern China sea	*Penicillium chrysogenum* QEN-24S	alga	[62]
67	Jiaozhou Bay,China	*Spicaria elegans* KLA-03	sediment	[83]
68	GeoMun Island, Yeosu, Korea	*Aspergillus flavu*	alga	[35]
69	Wenchang, Hainan province of China	*Wallemia sebi* PXP-89	mangrove	[25]
70	Dongzhai mangrove, Hainan, China	*Fusarium* sp. DZ-27	mangrove	[115]
71	Mandovi estuary, Goa, India	*Microdochium nivale*	other plant	[107]
72	Weizhou Island of southern China sea	*Penicillium chrysogenum* QEN-24S	alga	[90]
73	Osaka University	*Trichoderma* sp.	sponge	[37,38]
74	the coast of Zhanjiang, Guangdong province	*Aspergillus* sp. No. FSY-01 and FSW-02	mangrove	[28,118]
75	Yantai, China	*Aspergillus oryzae*	alga	[16]
76	Coast of Jeju Island, Korea	*Aspergillus versicolor*	water	[101]
77	Greifswalder Bodden, Baltic Sea, Germany	*Lophiostoma* sp. 226	driftwood	[120]
78	Sakhalin Bay (Sea of Okhotsk),	*Myceliophthora lutea*	sediment	[84]
79	South China Sea coast	fungus E33 and K38	mangrove	[49,50]
80	South China Sea	*Trichoderma koningii*	sediment	[59]
81	coral reef at Palau	The fungus 98F134	sponge	[94]
82	Pulau Tinggi, Malaysia	*Fusarium proliferatum*	sponge	[98]
83	GeoMun Island, Yeosu, Korea	*Fusarium tricinctum*	alga	[66]
84	Tinggi Island, Malaysia	*Paecilomyces* sp.	sponge	[36]
85	Dalian, China	*Aspergillus unguis* DLEP2008001	other plant	[100]
86	London Island of Kongsfjorden of NyAlesund District (altitude of 100m) of Arctic	*Eutypella* sp. D-1	soil	[46]

Table S1. *Cont.*

87	Zhoushan Island	*Aspergillus ustus* cf-42	alga	[40]
88	Xisha Islands coral reef of the South China Sea	*Aspergillus versicolorstrain* ATCC 9577	coral	[30]
89	Hainan Island in the South China Sea	*Alternaria tenuissima* EN-192	mangrove	[111]
90	the coast of Atami-shi,Shizuoka Prefecture, Japan	*Stagonosporopsis cucurbitacearum*	sponge	[26]
91	Xisha Islands coral reef in the South China Sea	*Aspergillus* sp. XS-20090066	coral	[82]
92	South China Sea, Sanya Hainan Province, China	*Penicillium* sp. SCSGAF 0023	coral	[93]
93	Yokji Island, Gyeongnam Province Korea	*Dothideomycete* sp.	alga	[88]
94	Gagu-do, Korea	*Acremonium strictum*	sponge	[69]
95	the southern China Sea	*Penicillium commune* QSD-17	sediment	[77]
96	Sakhalin Bay (the Sea of Okhotsk	*Aspergillus versicolor* (Vuill.) Tirab.	animals	[110]
97	South China Sea coast	*Aspergillus* sp.	mangrove	[65]
98	Waikiki Beach, Honolulu, Hawaii.	*Aspergillus* sp.	soil	[32]
99	South China Sea	*Penicillium commune* SD-118	sediment	[95]
100	Van Phong Bay (South China Sea)	*Penicillium citrinum*	coral	[121]

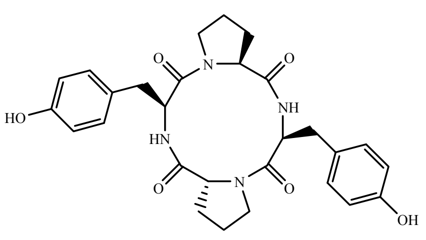

1

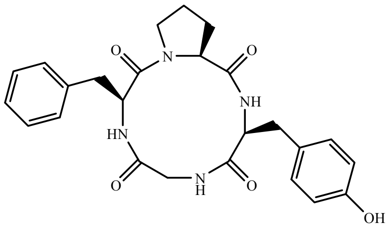

2

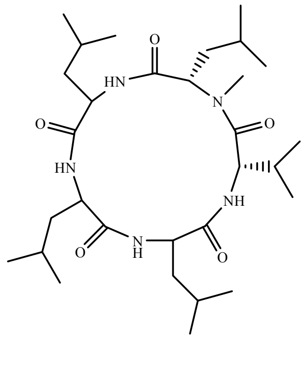

3

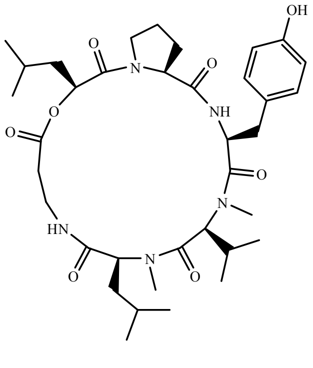

4

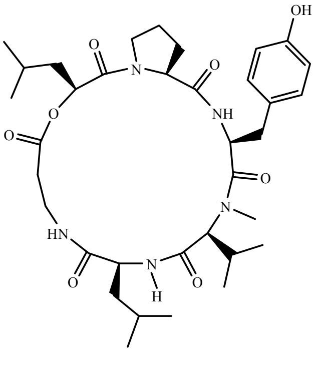

5

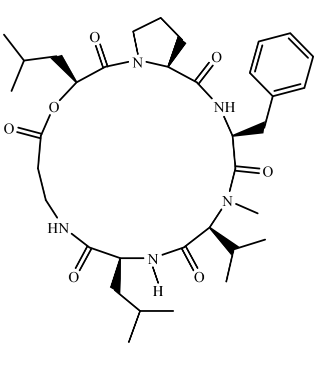

6

Figure S1. *Cont.*

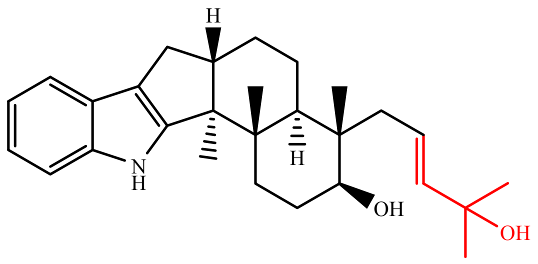

7

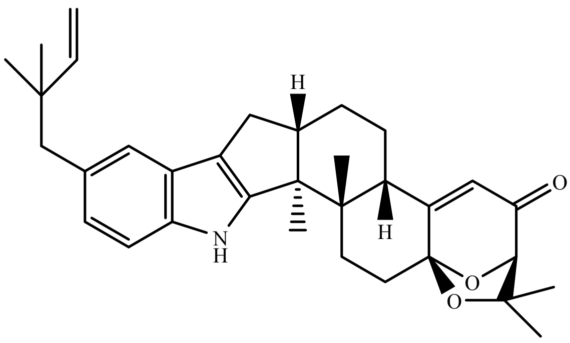

8

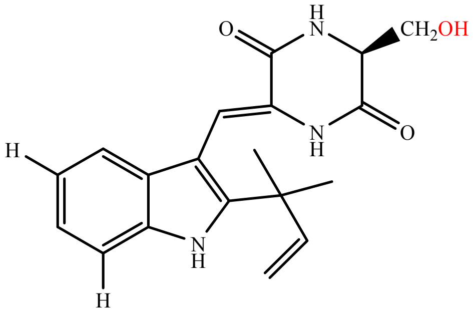

9

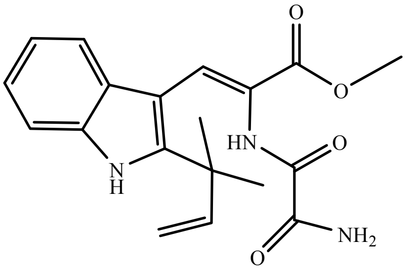

10

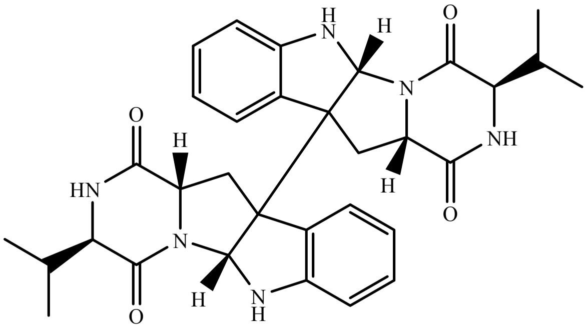

11

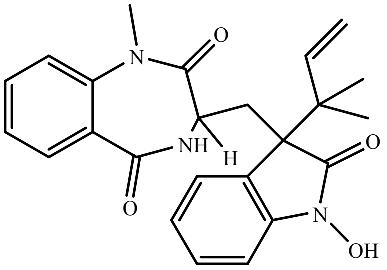

12

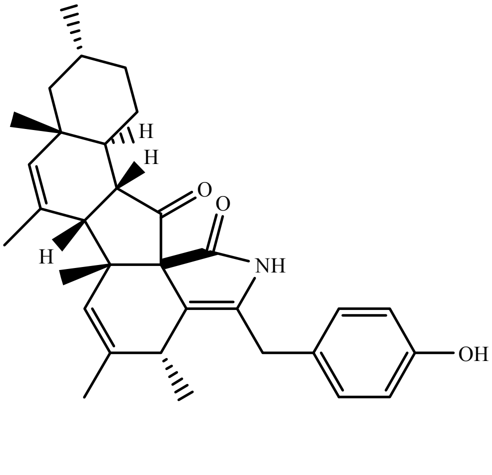

13

Figure S1. *Cont.*

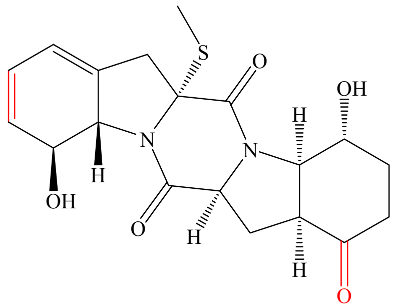

14

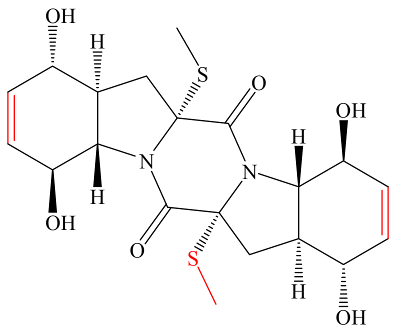

15

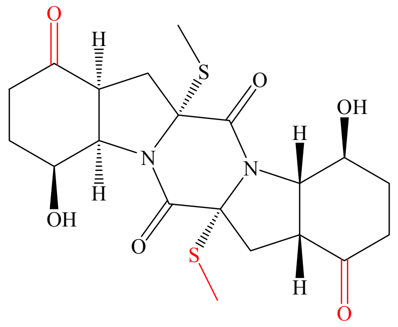

16

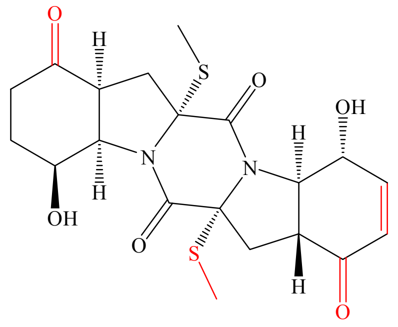

17

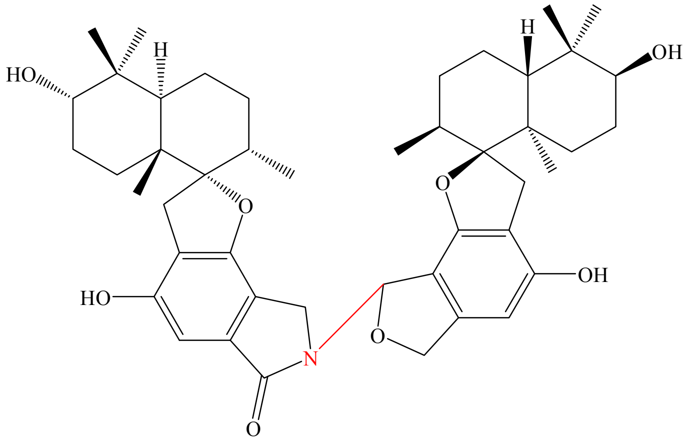

18

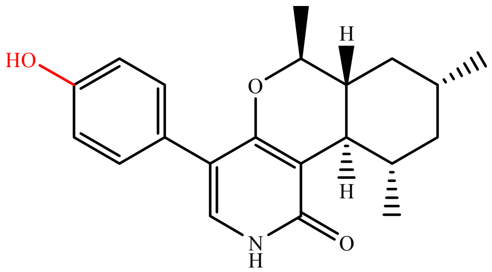

19

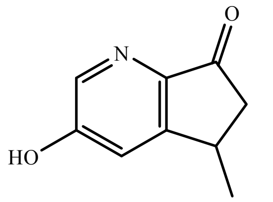

20

Figure S1. *Cont.*

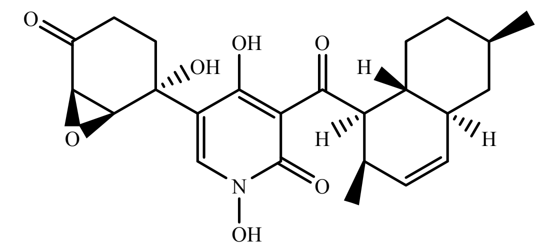

21

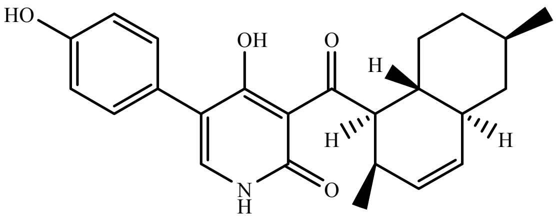

22

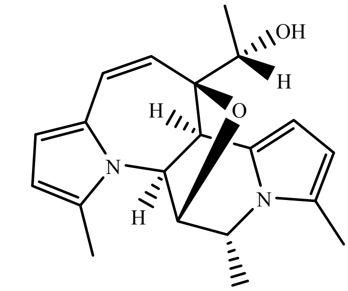

23

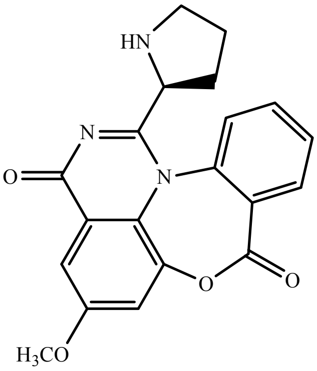

24

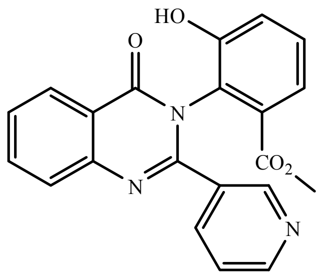

25

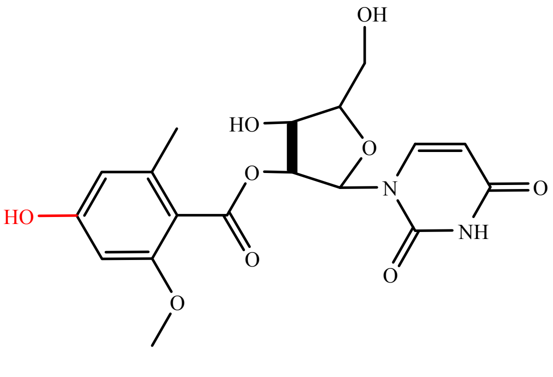

26

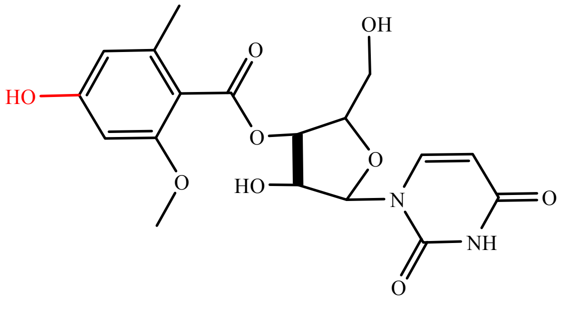

27

Figure S1. *Cont.*

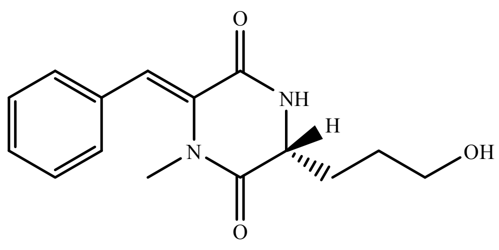

28

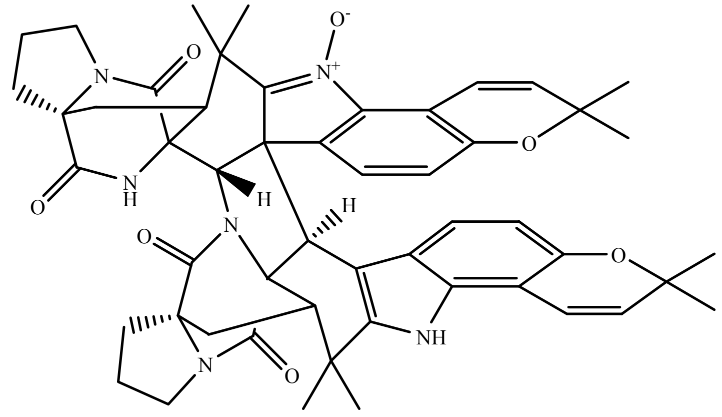

29

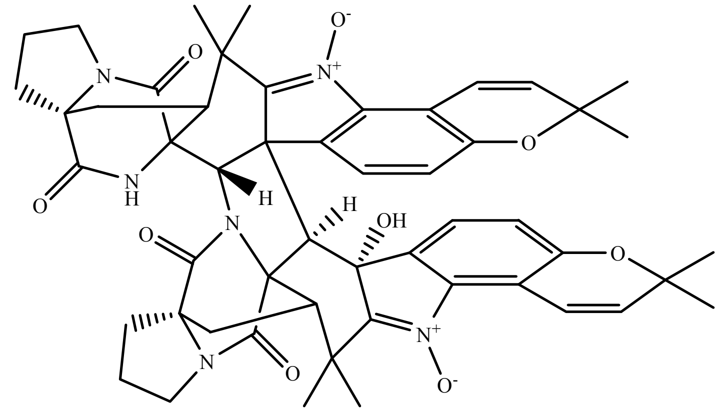

30

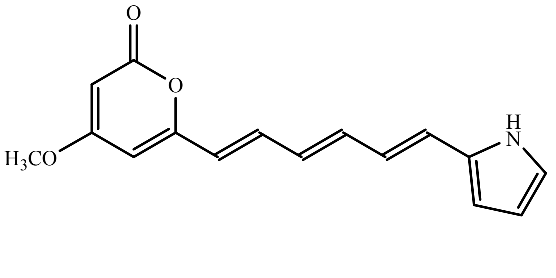

31

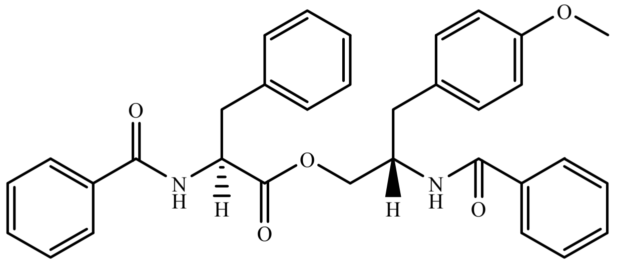

32

Figure S1. *Cont.*

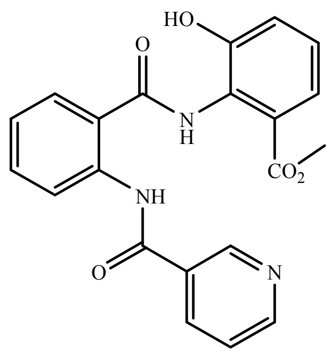

33

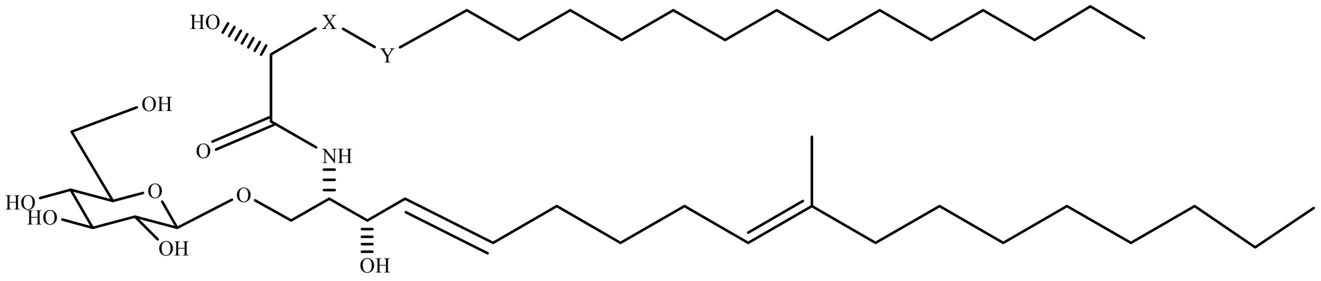

34:X-Y=CH_2CH_2
35:X-Y=CH=CH

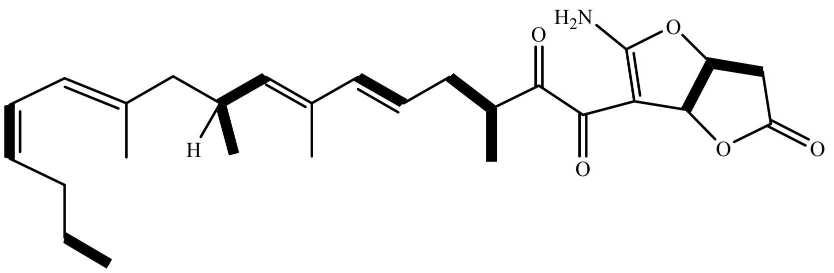

36

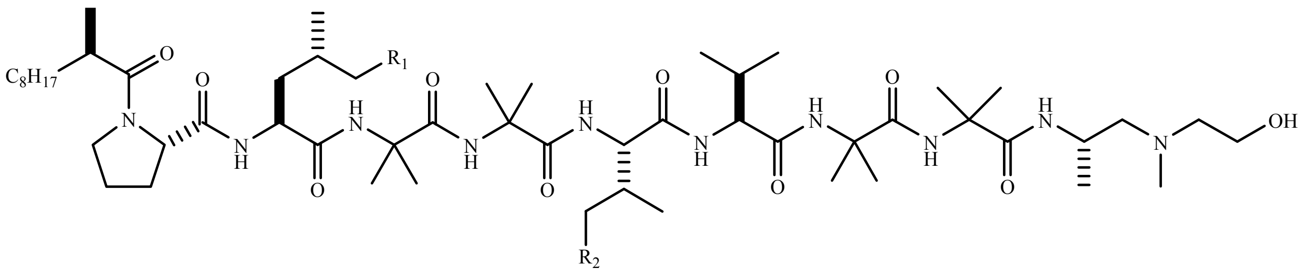

37:R_1= 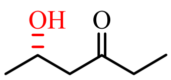 R_2=CH_3 **38**:R_1= 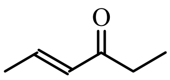 R_2=CH_3 **39**:R_1= 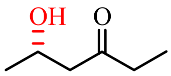 R_2=H

Figure S1. *Cont.*

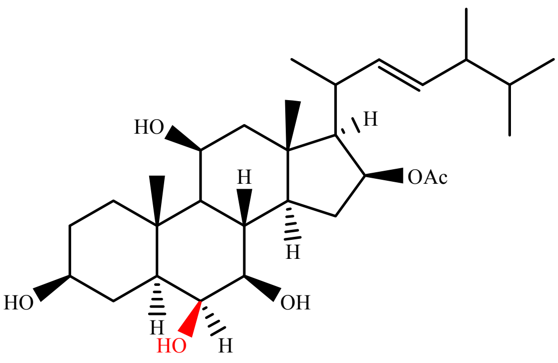

40

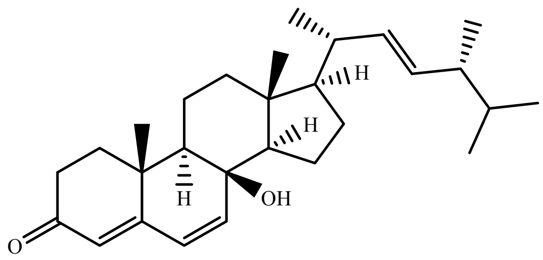

41

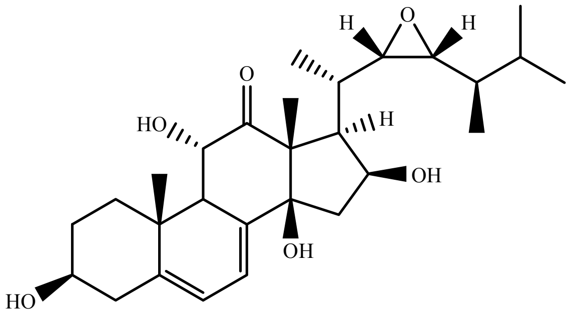

42

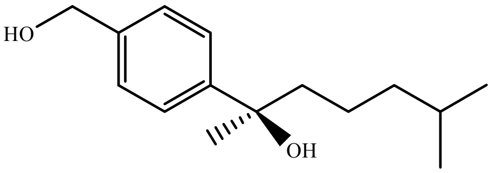

43

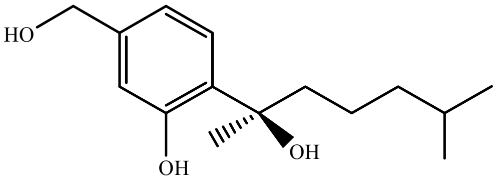

44

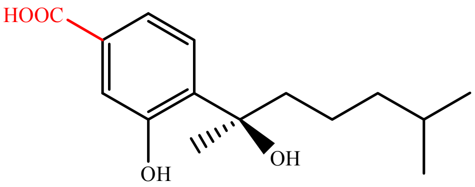

45

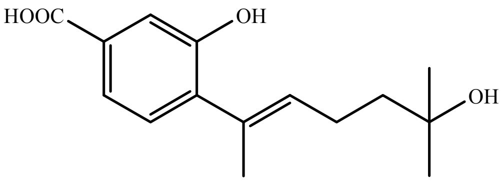

46

Figure S1. *Cont.*

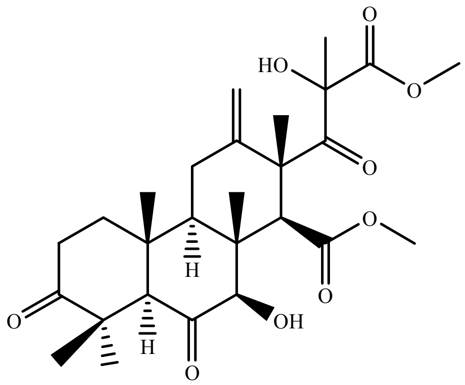

47

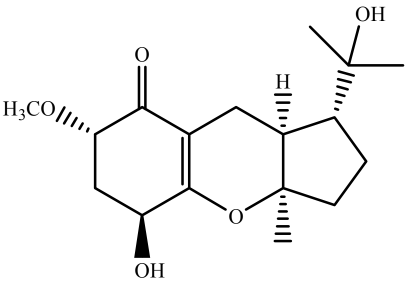

48

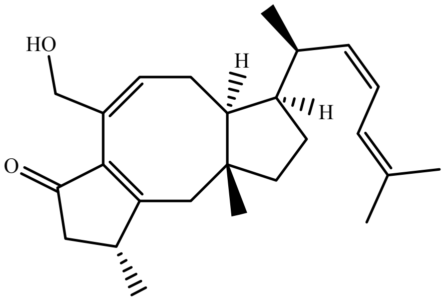

49

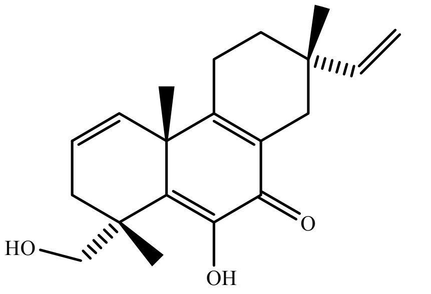

50

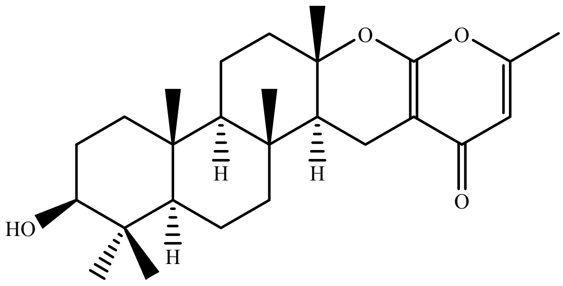

51

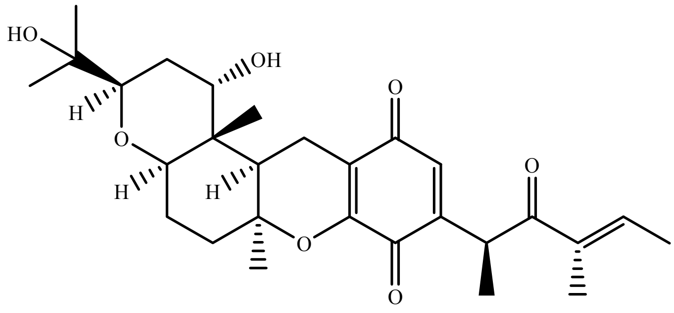

52

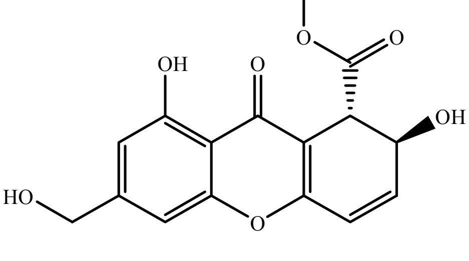

53

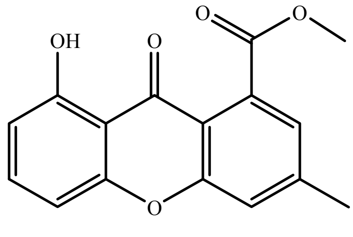

54

Figure S1. *Cont.*

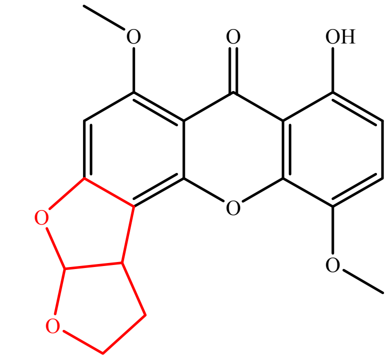

55

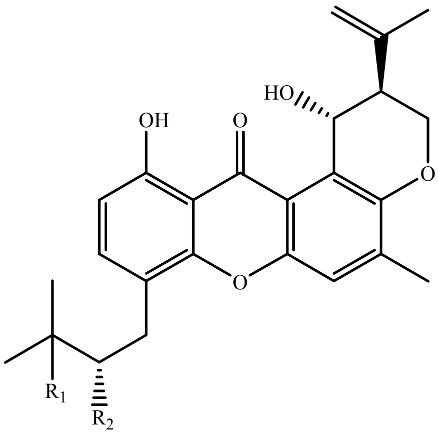

56:R_1=Cl,R_2=OH
57:R_1=OCH_3,R_2=OCH_3
58:R_1=OH,R_2=OAc

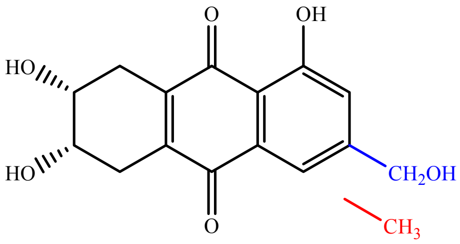

59

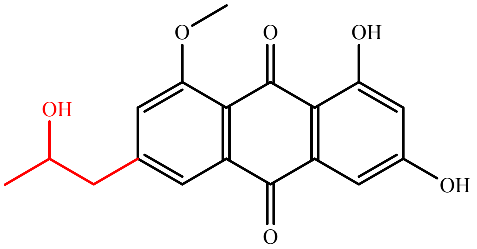

60

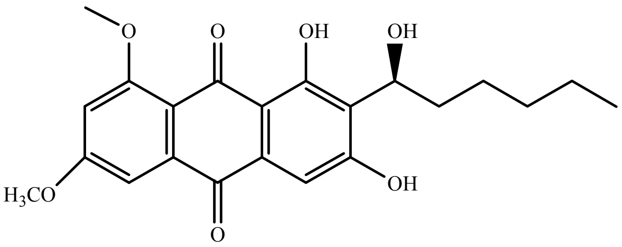

61

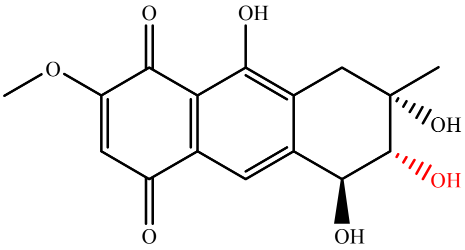

62

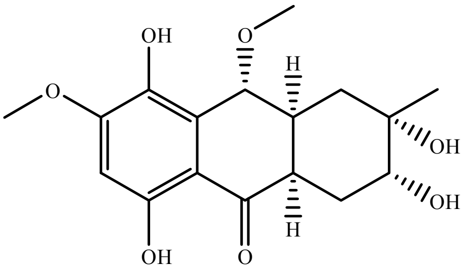

63

Figure S1. *Cont.*

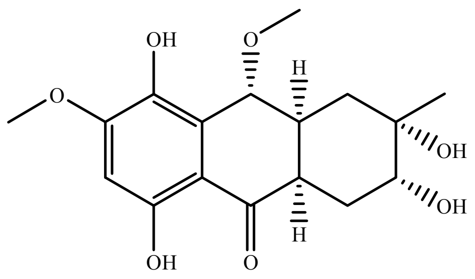

64

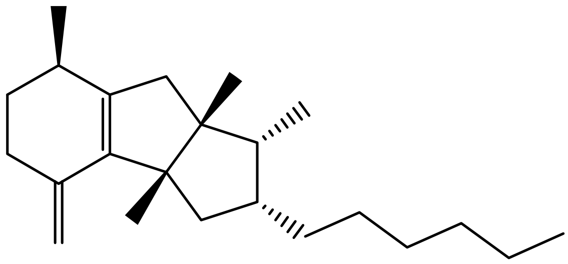

65

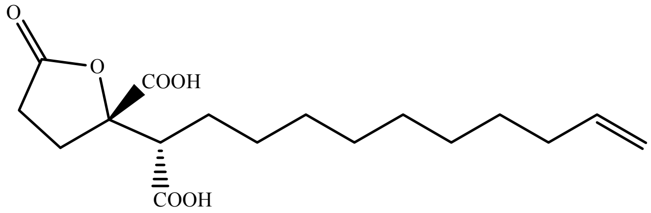

66

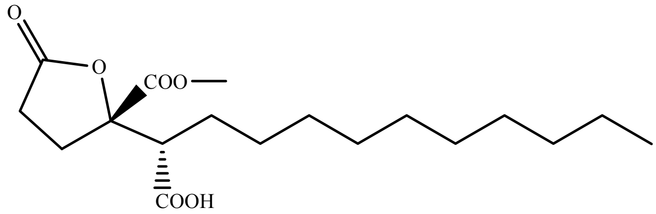

67

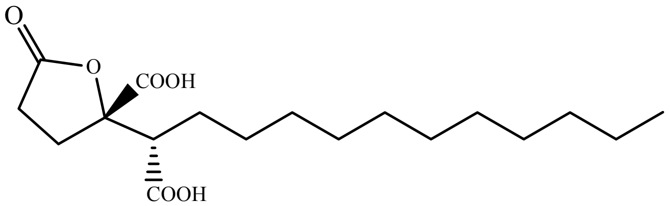

68

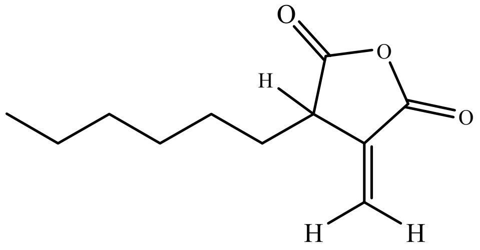

69

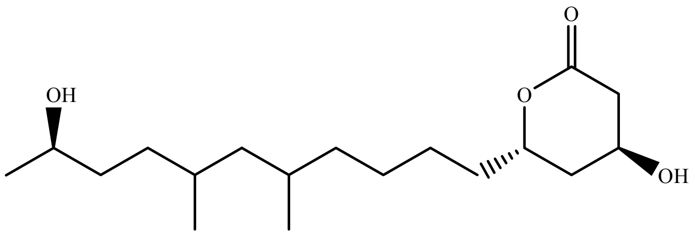

70

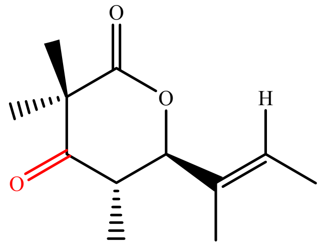

71

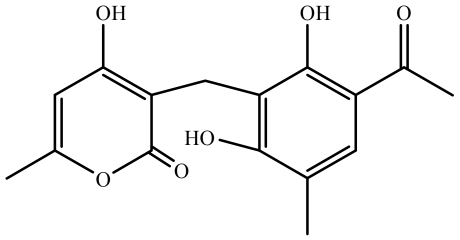

72

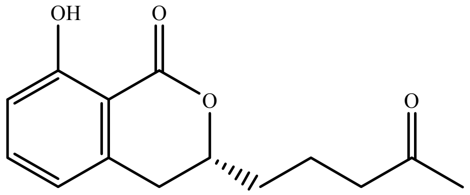

73

Figure S1. *Cont.*

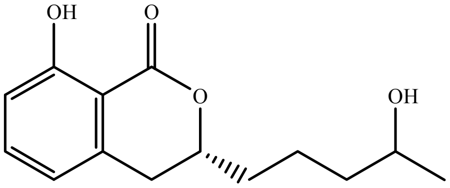

74

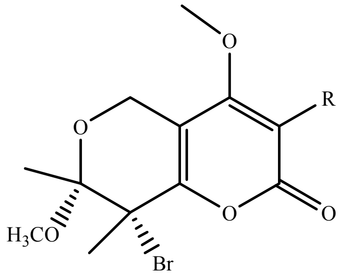

75:R=H **76**:R=Br

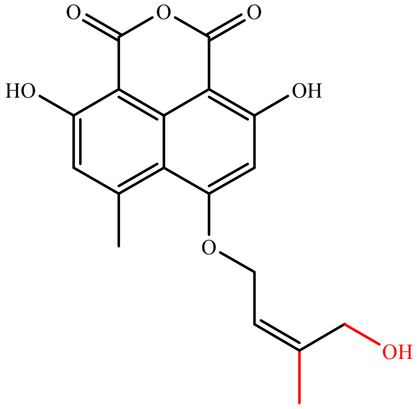

77

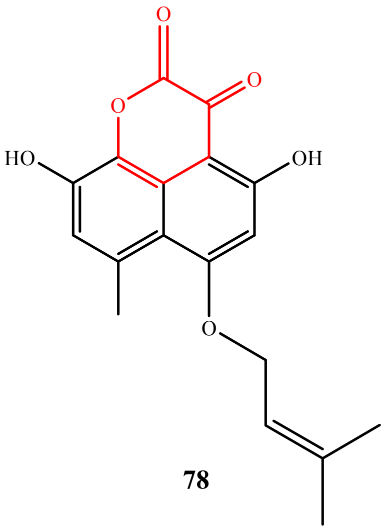

78

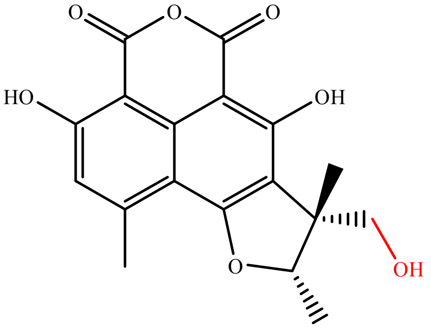

79

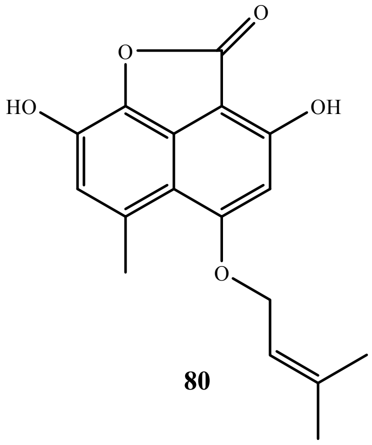

80

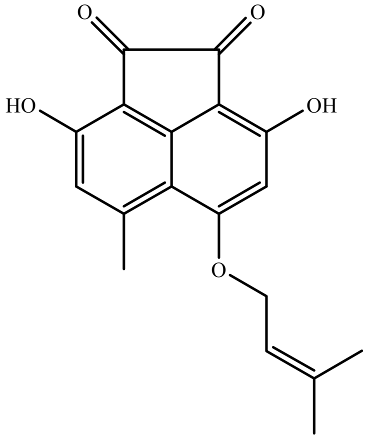

81

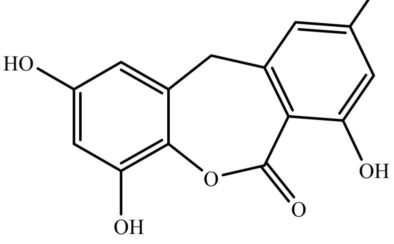

82

Figure S1. *Cont.*

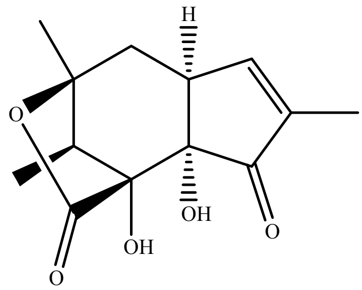

83

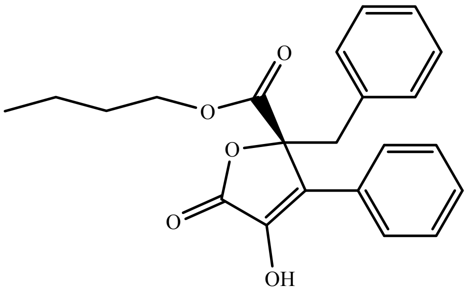

84

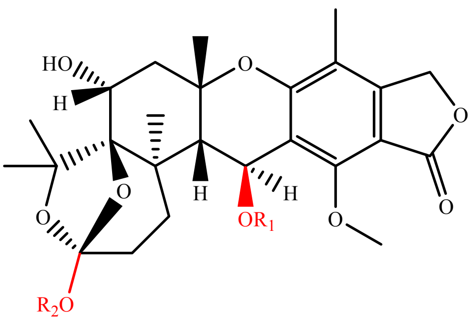

85:R1=H,R2=H
86:R1=R2=Me
87:R1=Ac,R2=Me

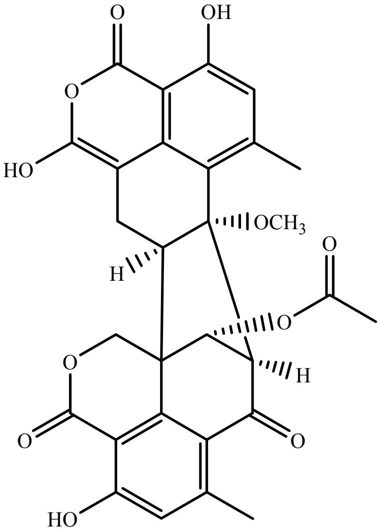

88

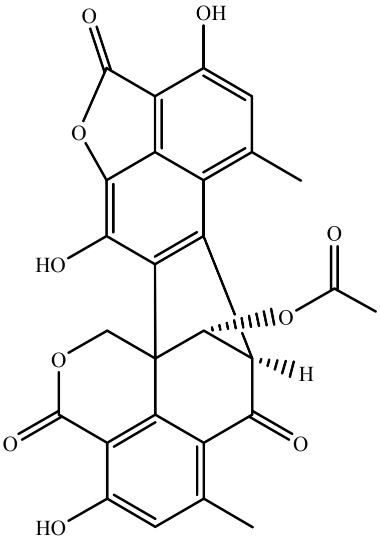

89

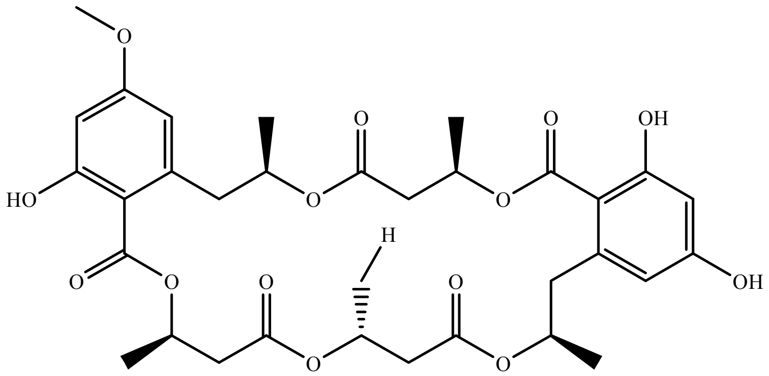

90

Figure S1. *Cont.*

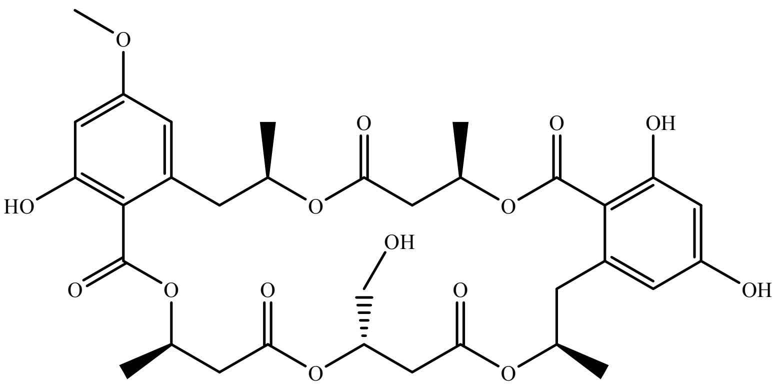

91

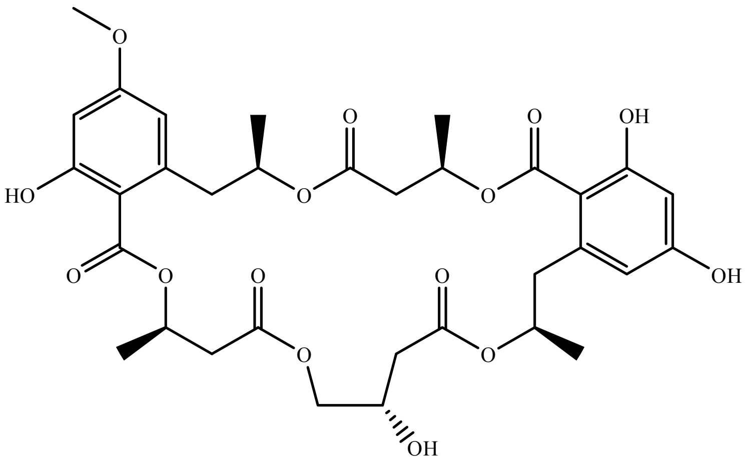

92

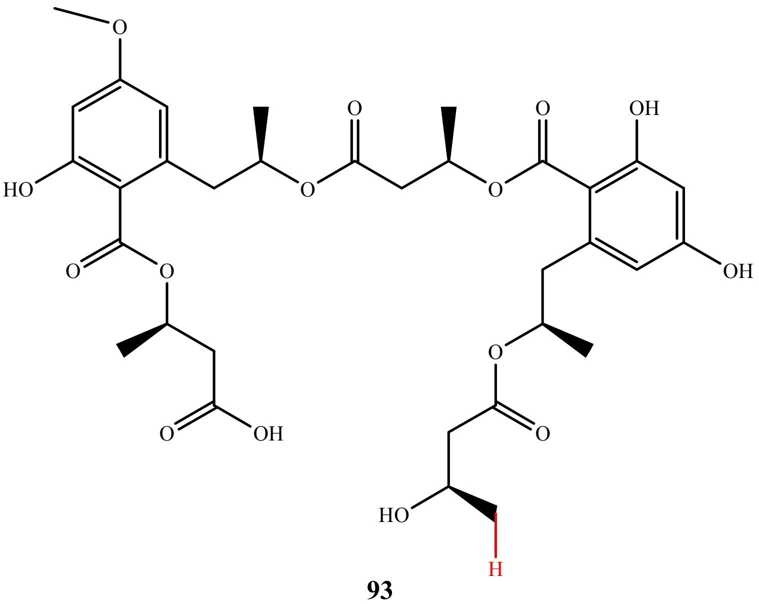

93

Figure S1. *Cont.*

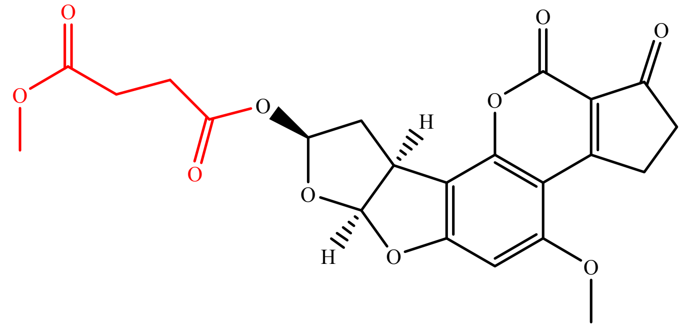

94

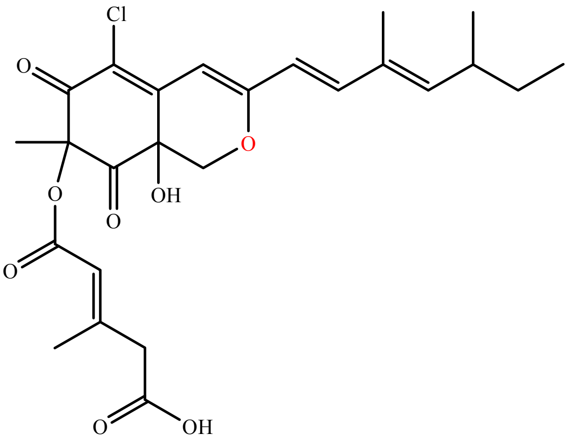

95

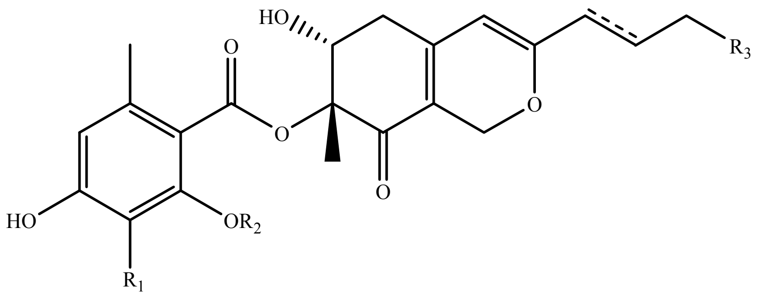

96:R1=R3=H ,R2=Me
97:R1=OH, R2=Me, R3=H
98:R1=OH, R2=Me, R3=H, Δ^{10}

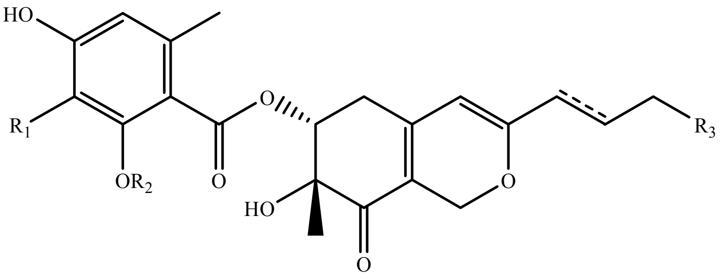

99:R1=R2=R3=H, Δ^{10}
100:R1=OH, R2=Me, R3=H,Δ^{10}
101:R1=OH, R2=Me, R3=H

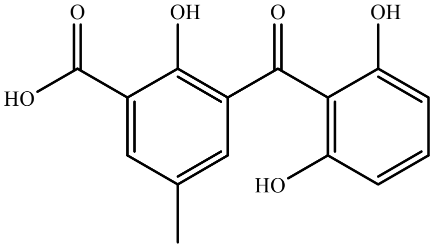

102

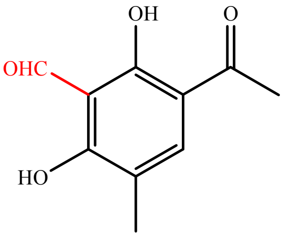

103

Figure S1. *Cont.*

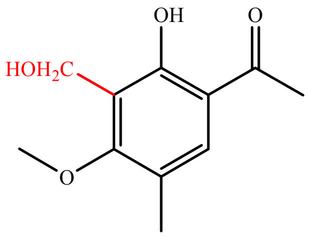

104

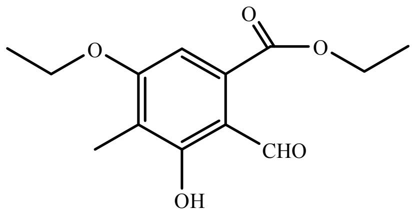

105

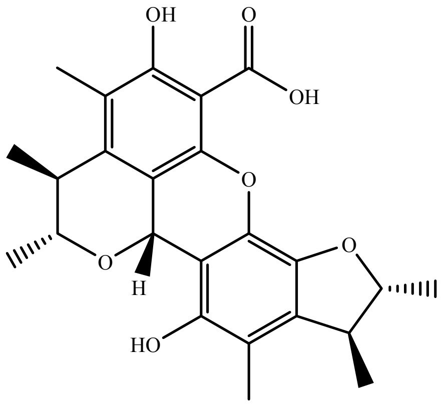

106

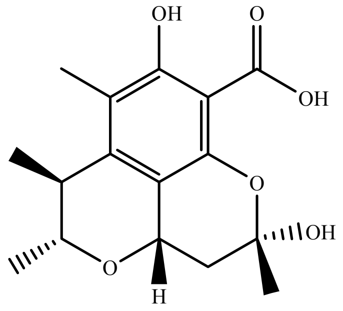

107

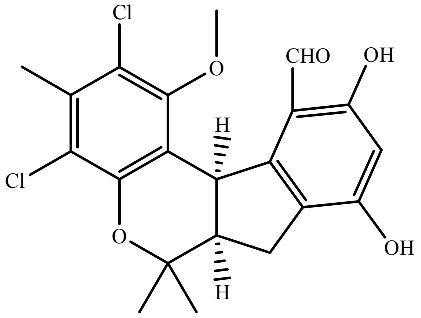

108

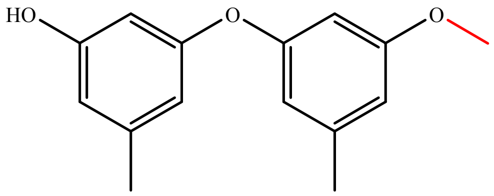

109

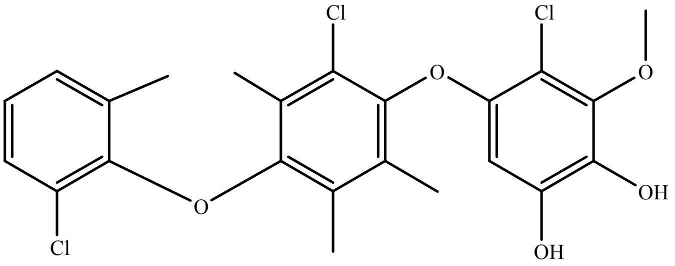

110

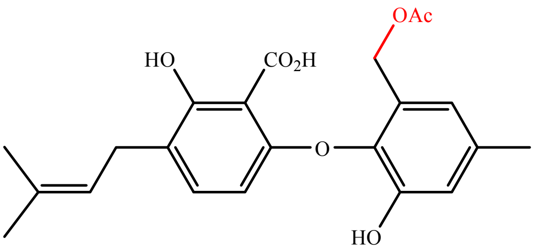

111

Figure S1. *Cont.*

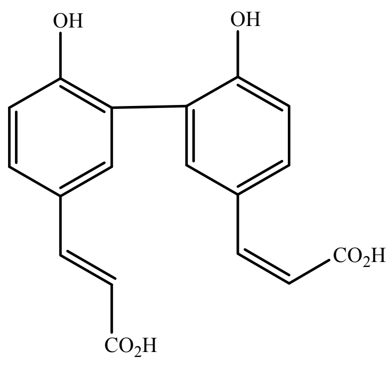

112

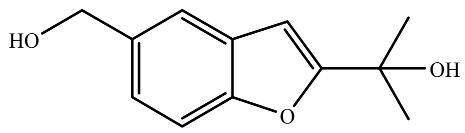

113

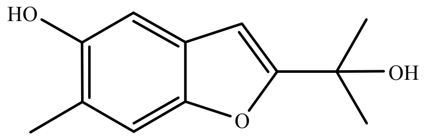

114

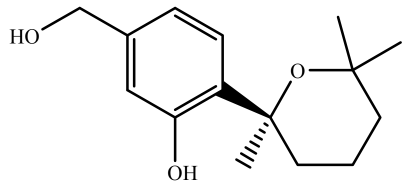

115

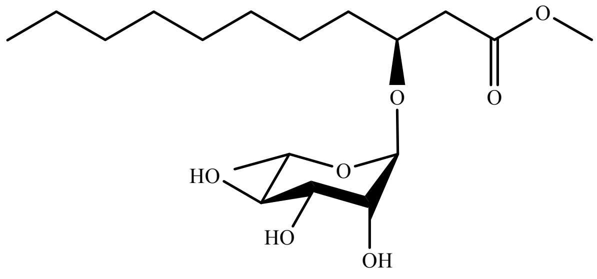

116

Figure S1. Structures of compound **1–116** (the red moiety of compound enhances the antimicrobial activity of the compound).

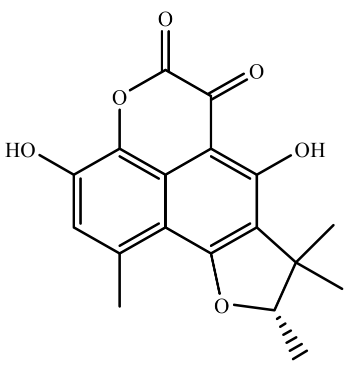

117

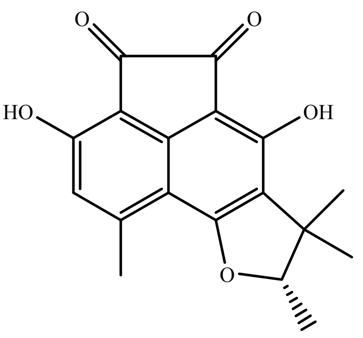

118

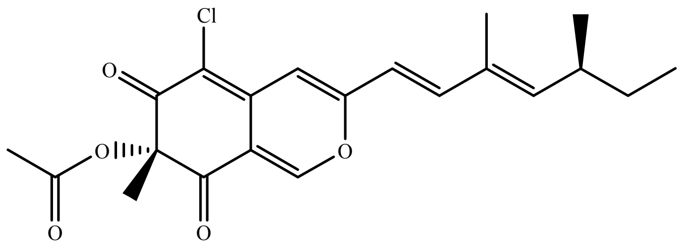

119

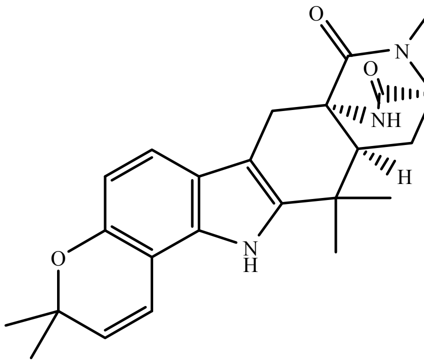

120

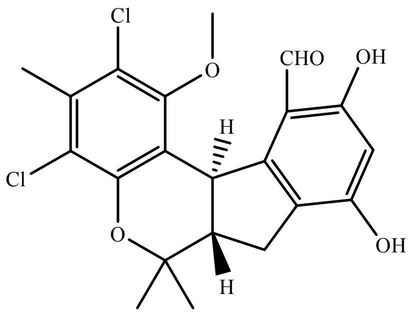

121

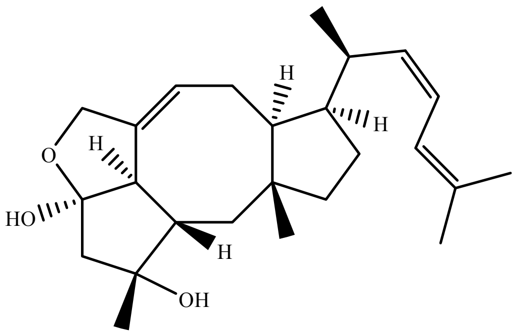

122

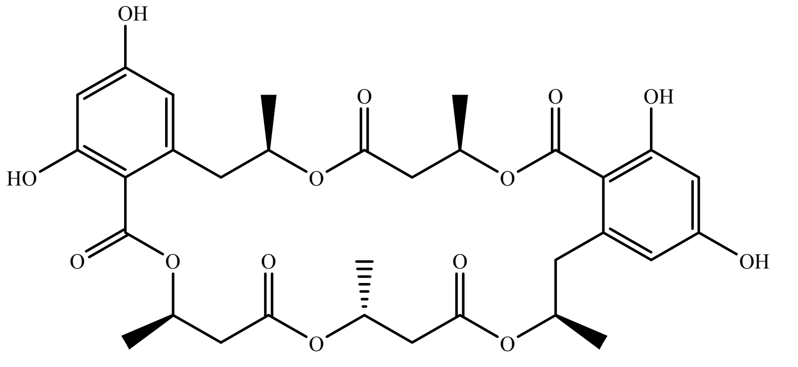

123

Figure S2. *Cont.*

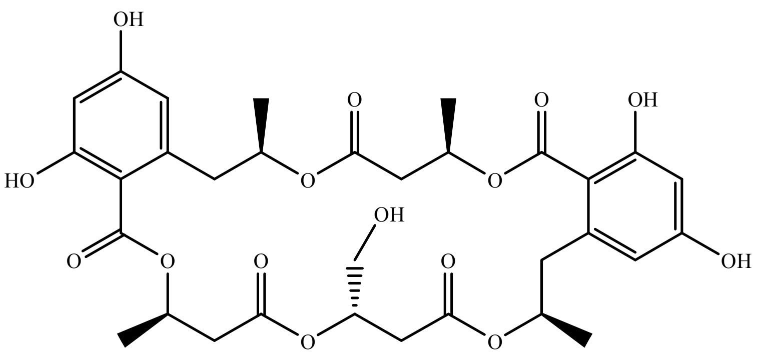

124

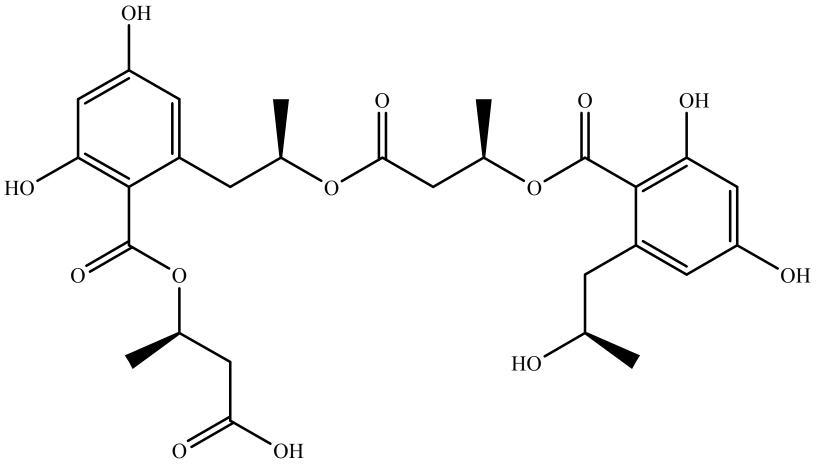

125

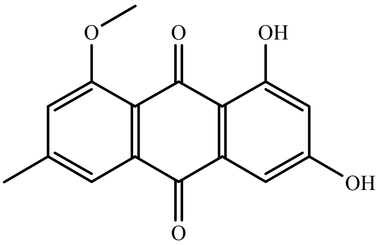

126

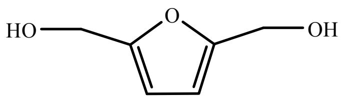

127

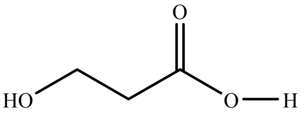

128

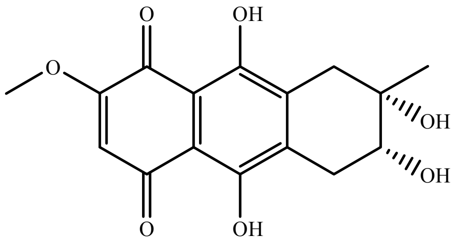

129

Figure S2. *Cont.*

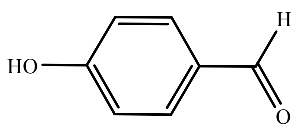

130

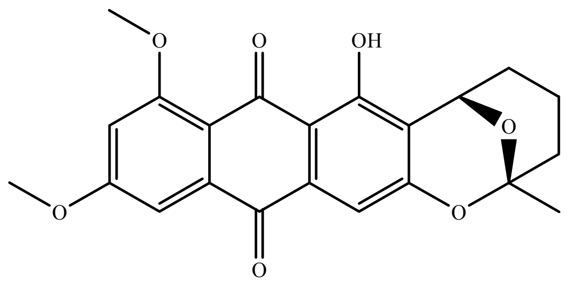

131

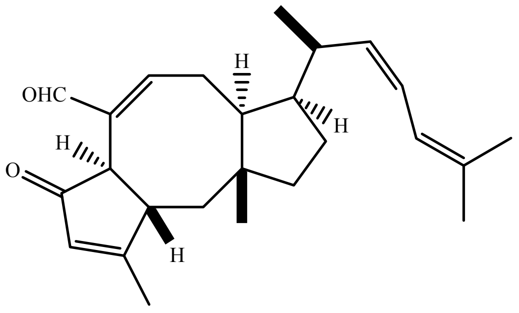

132

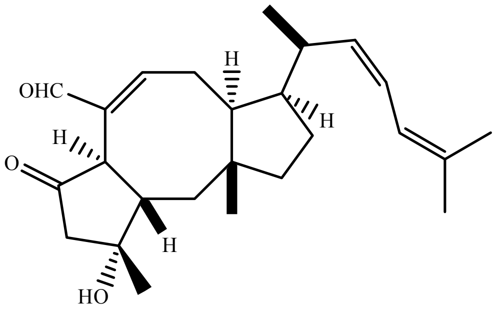

133

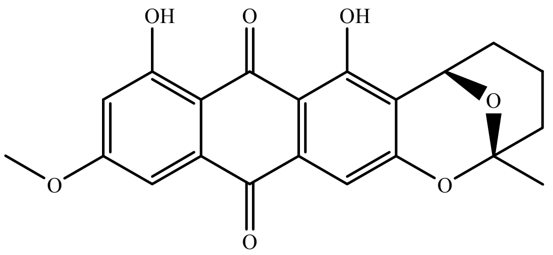

134

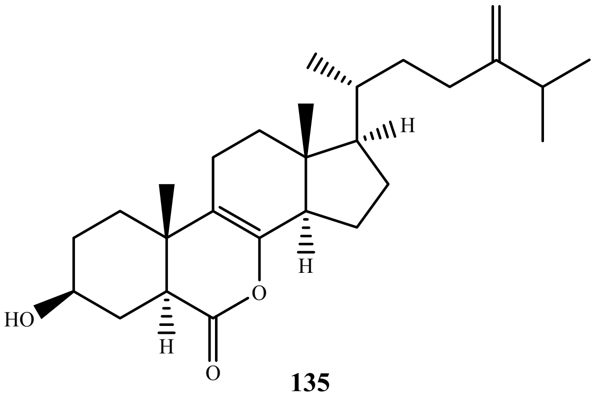

135

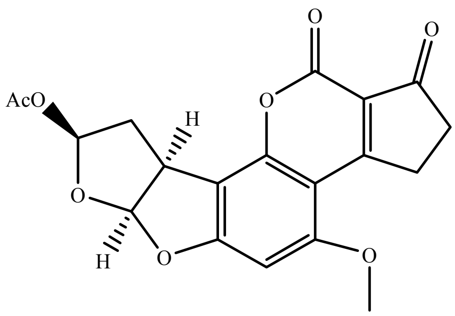

136

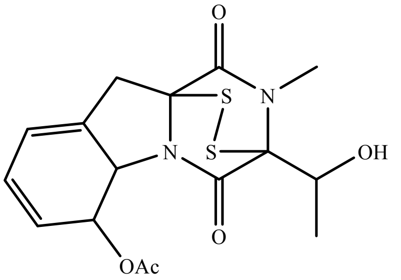

137

Figure S2. *Cont.*

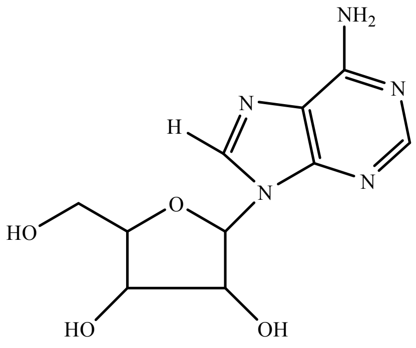

138

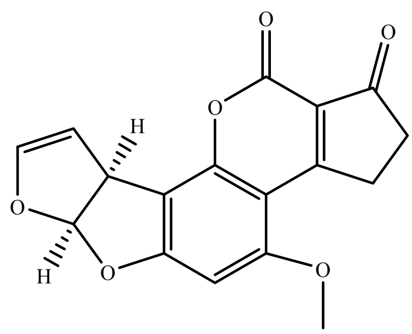

139

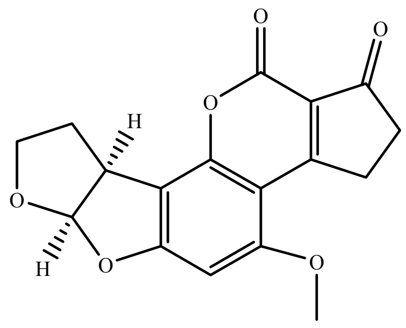

140

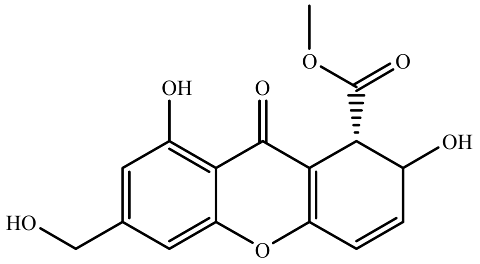

141

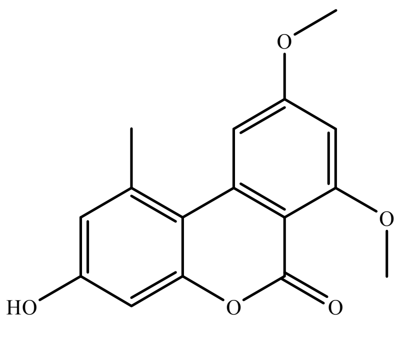

142

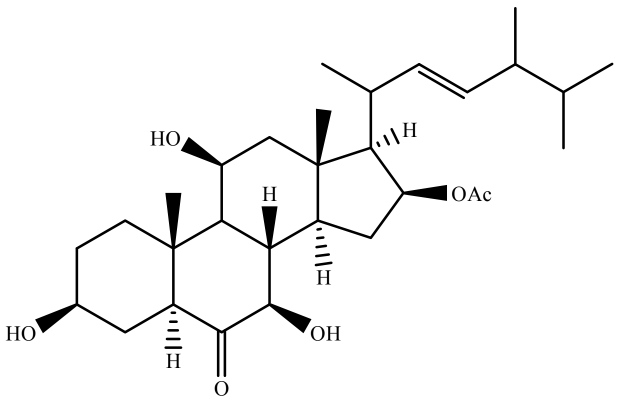

143

Figure S2. *Cont.*

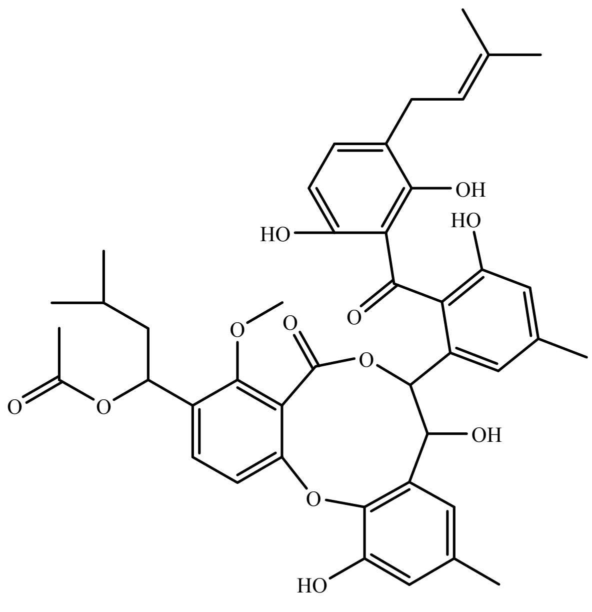

144

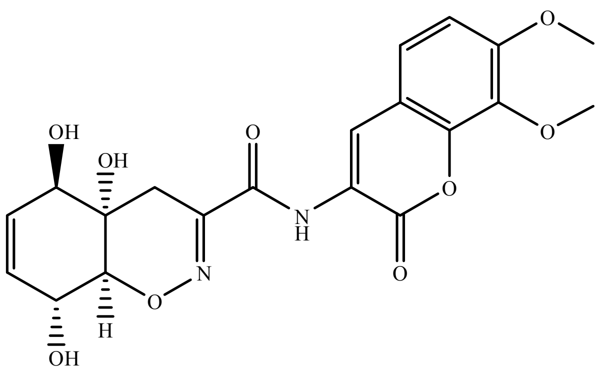

145

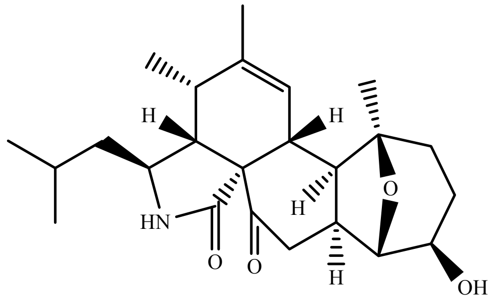

146

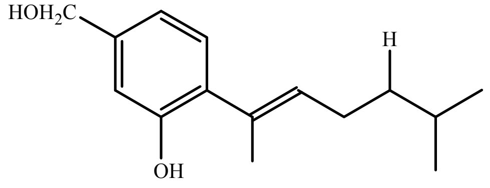

147

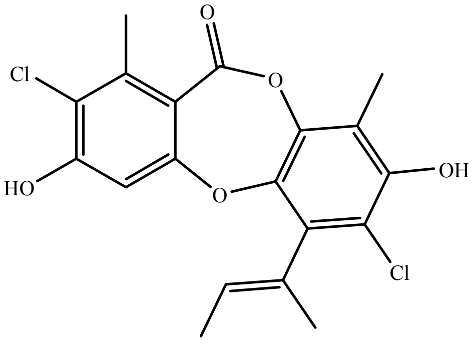

148

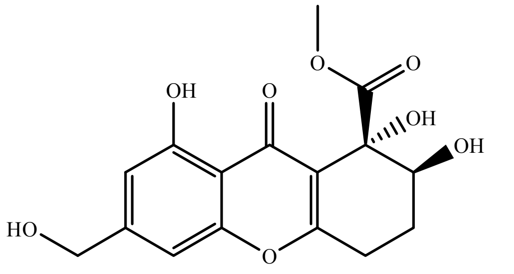

149

Figure S2. *Cont.*

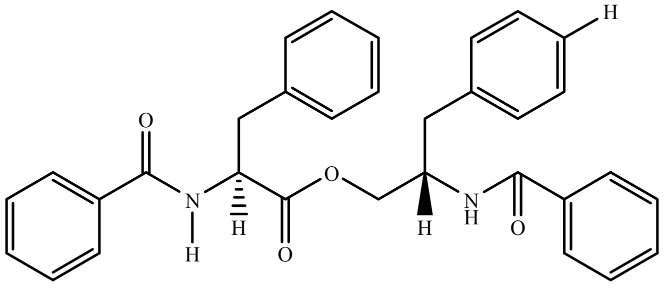

150

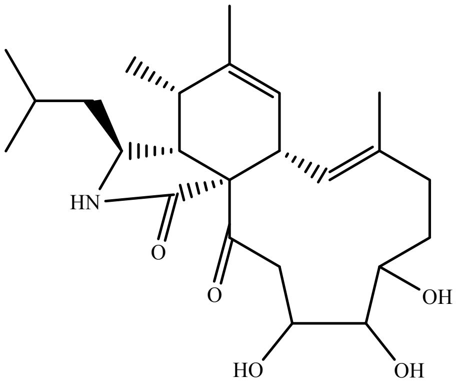

151

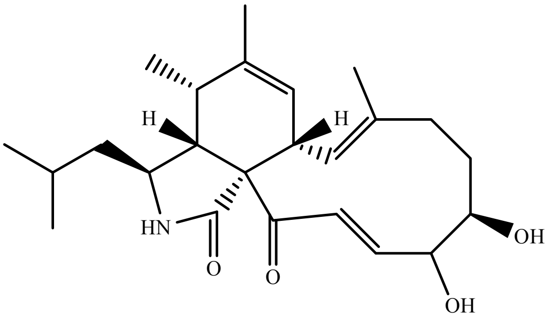

152

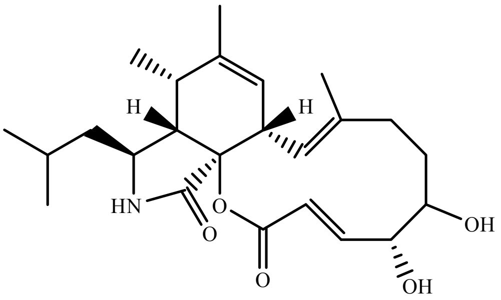

153

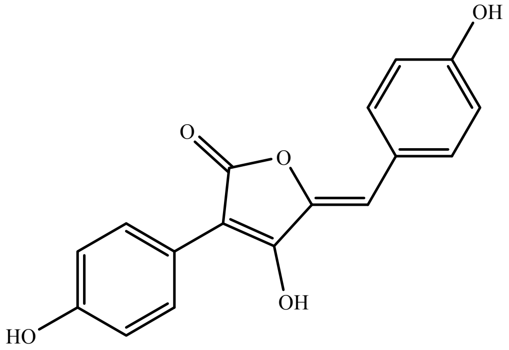

154

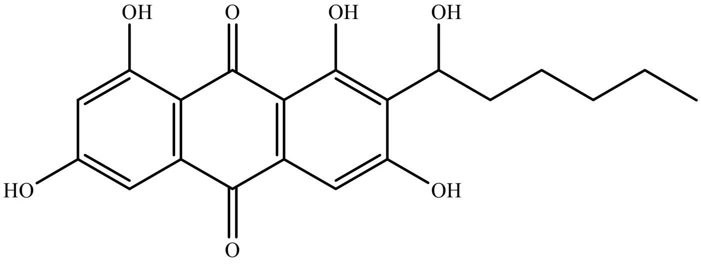

155

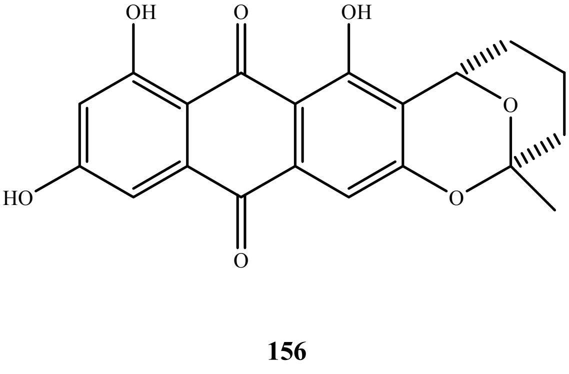

156

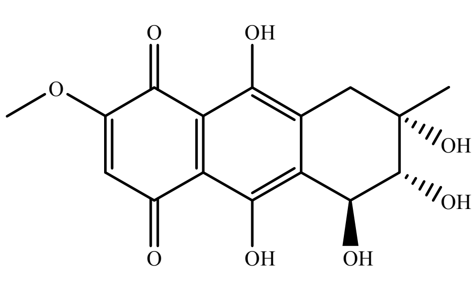

157

Figure S2. *Cont.*

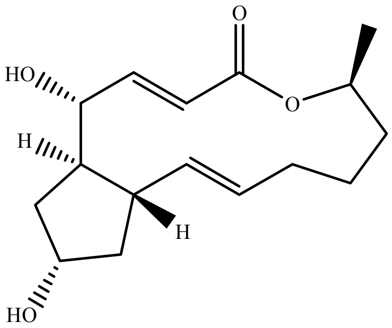

158

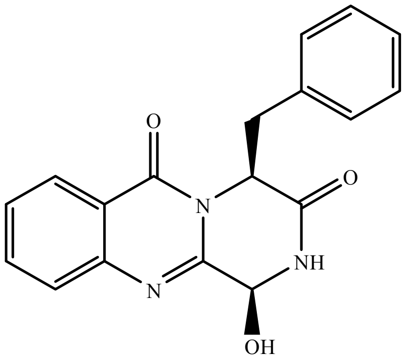

159

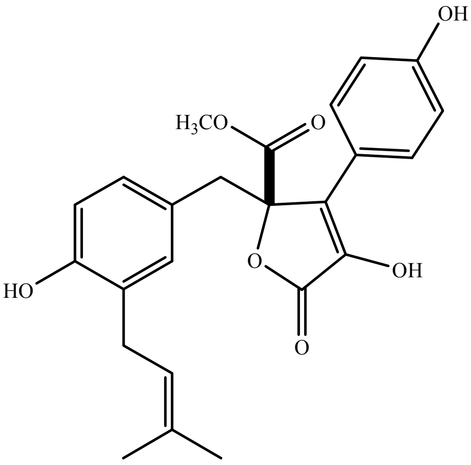

160

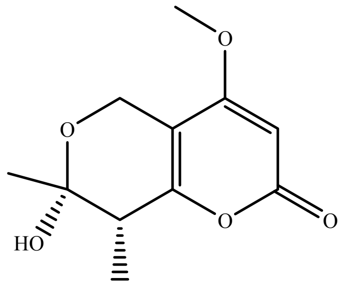

161

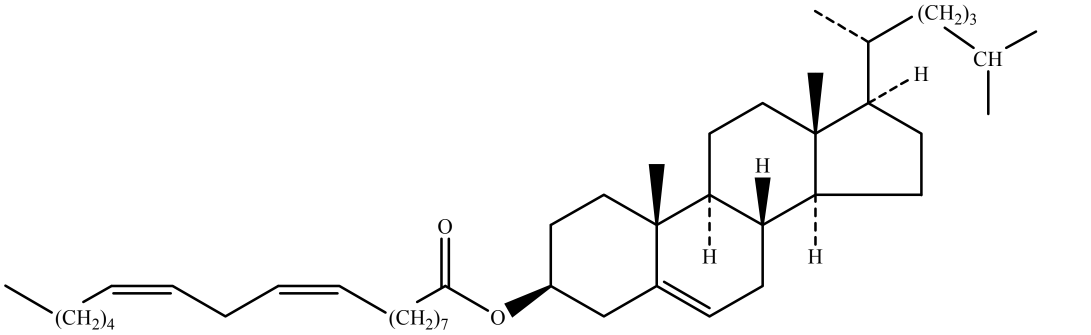

162

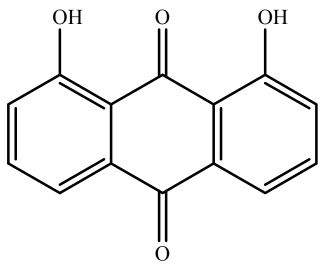

163

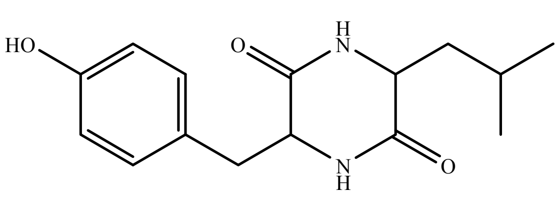

164

Figure S2. *Cont.*

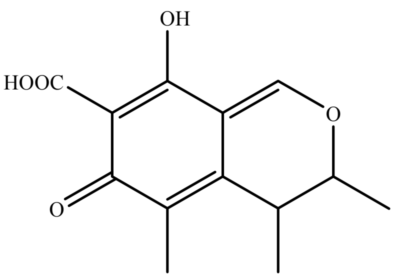

165

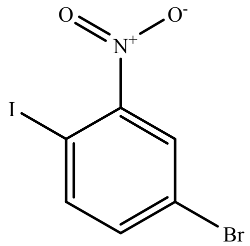

166

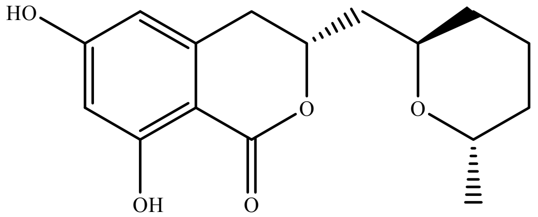

167

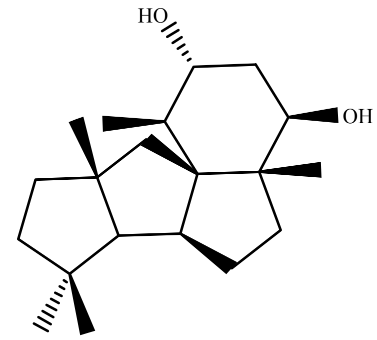

168

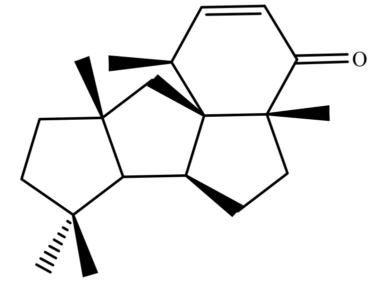

169

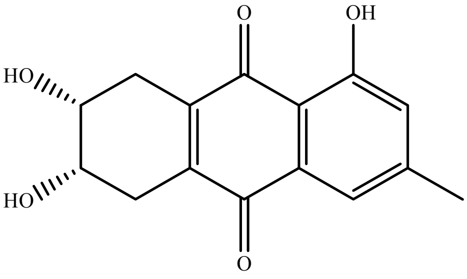

170

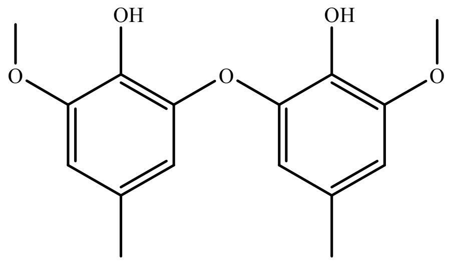

171

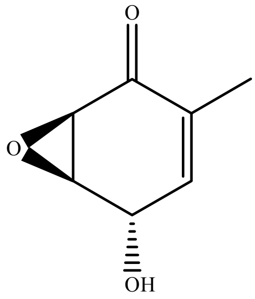

172

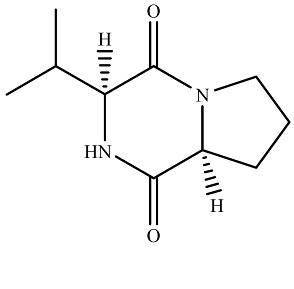

173

Figure S2. *Cont.*

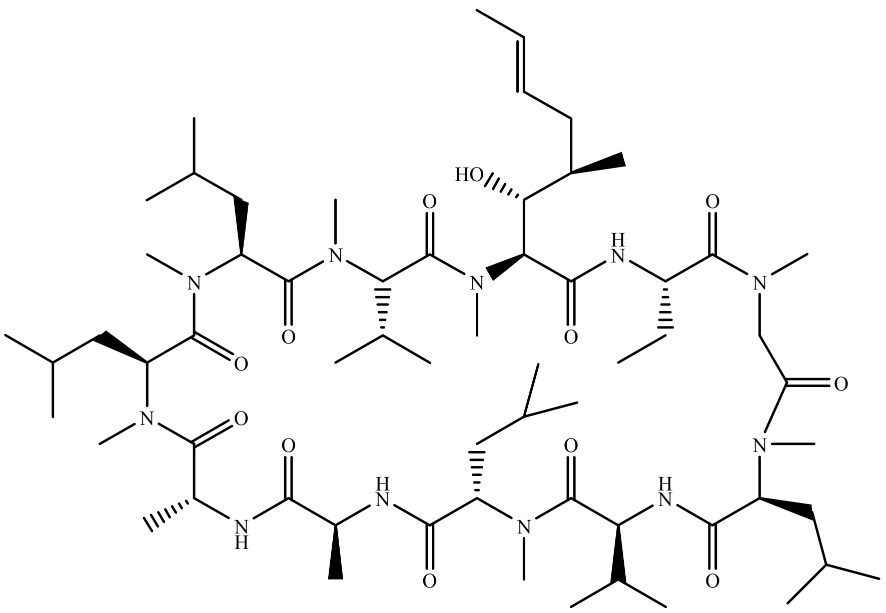

174

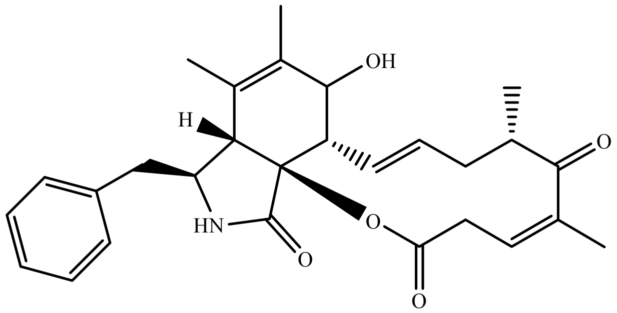

175

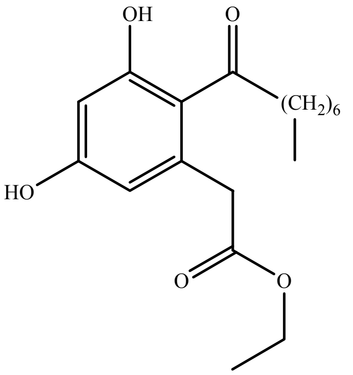

176

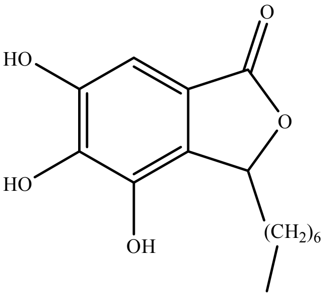

177

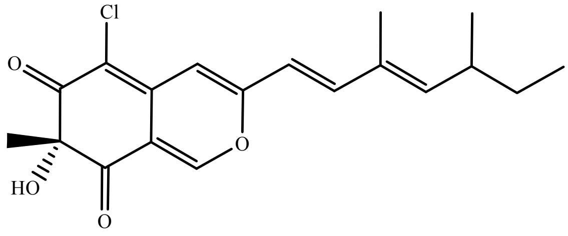

178

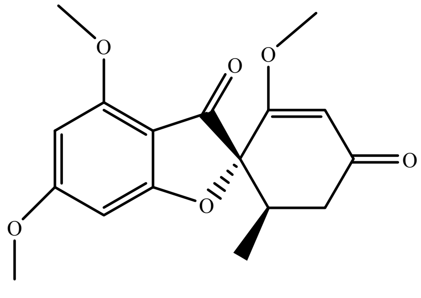

179

Figure S2. *Cont.*

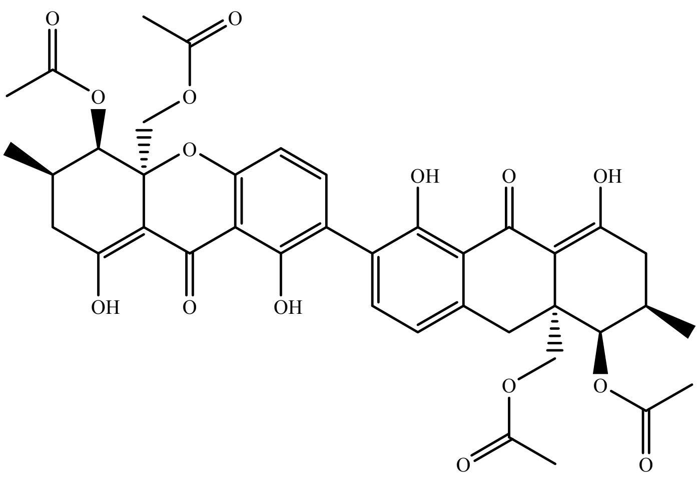

180

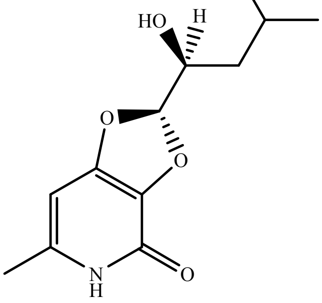

181

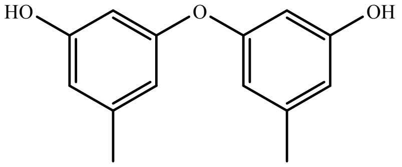

182

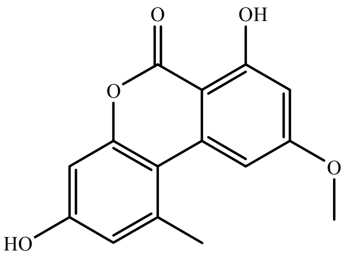

183

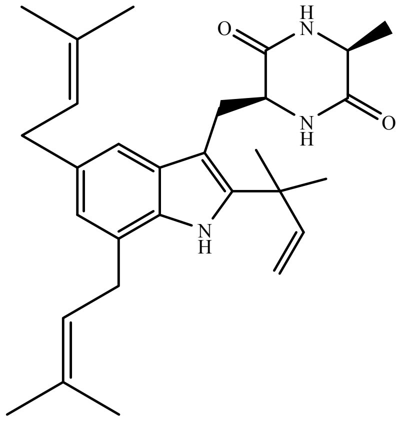

184

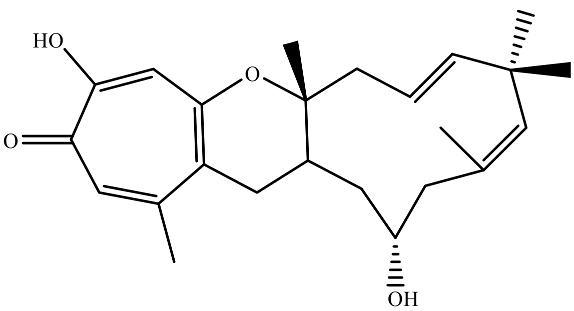

185

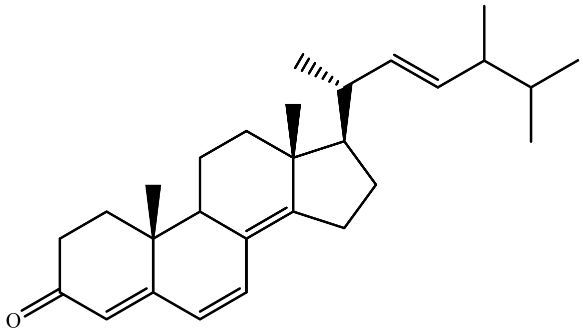

186

Figure S2. *Cont.*

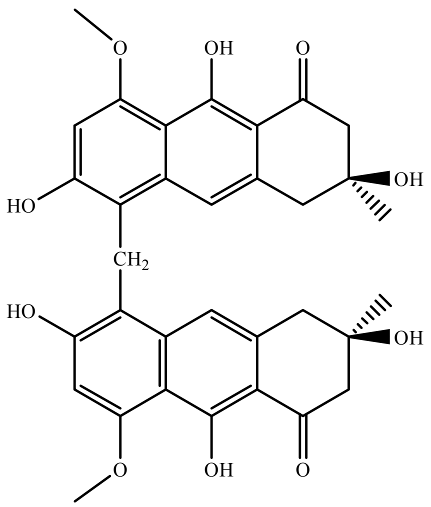

187

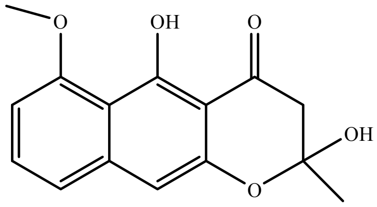

188

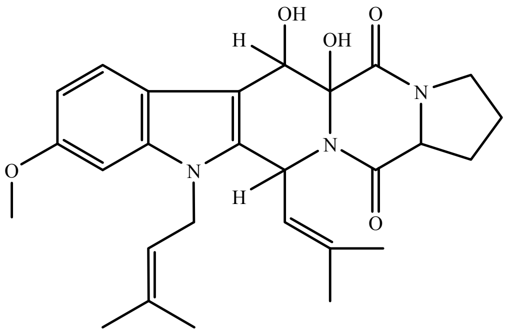

189

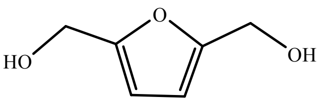

190

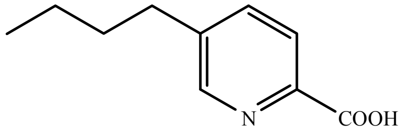

191

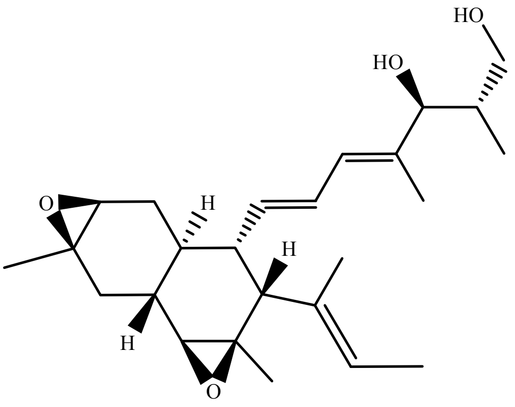

192

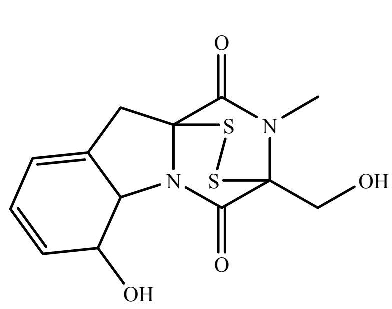

193

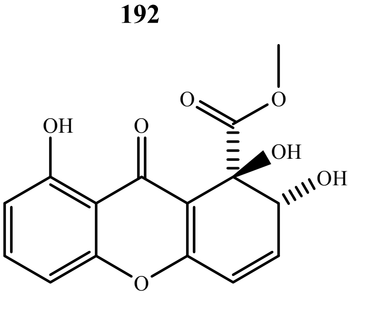

194

Figure S2. *Cont.*

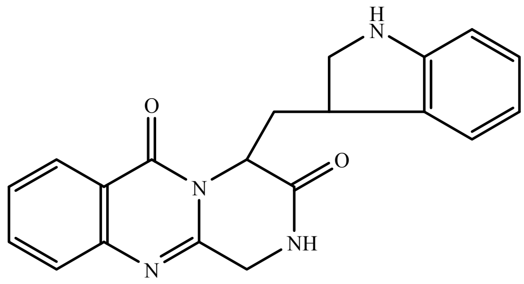

195

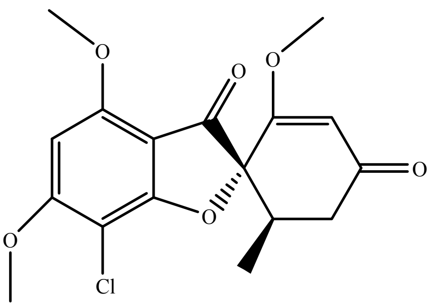

196

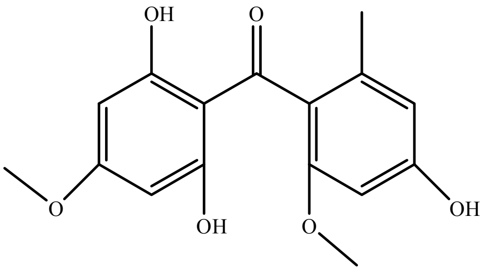

197

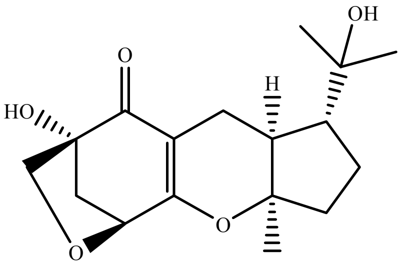

198

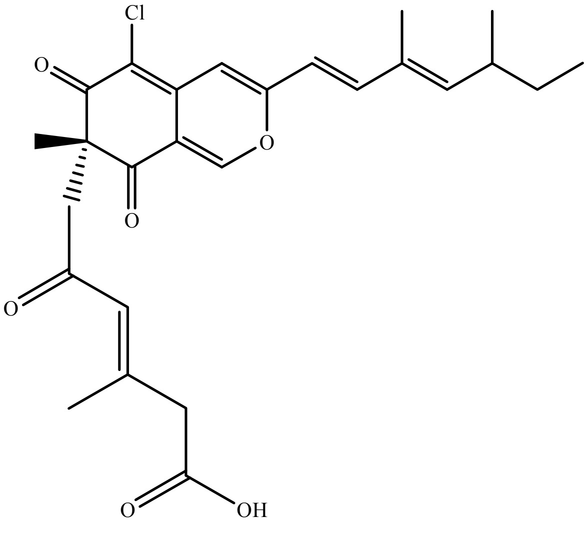

199

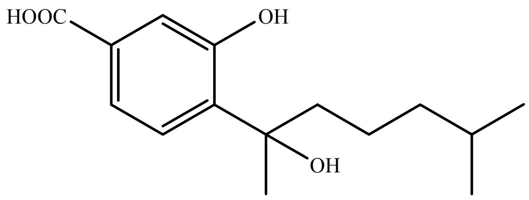

200

Figure S2. *Cont.*

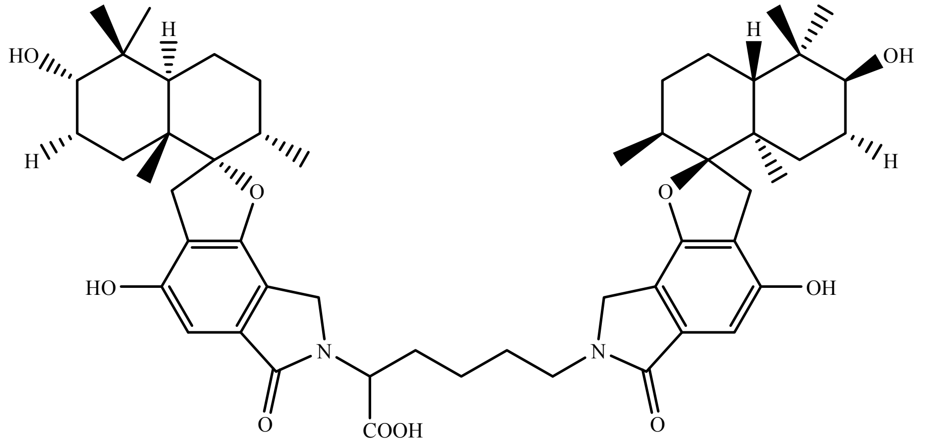

201

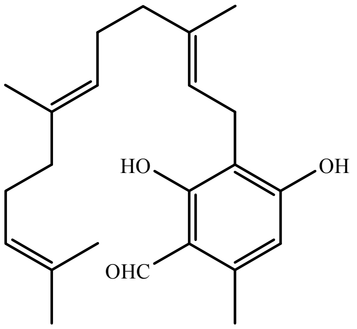

202

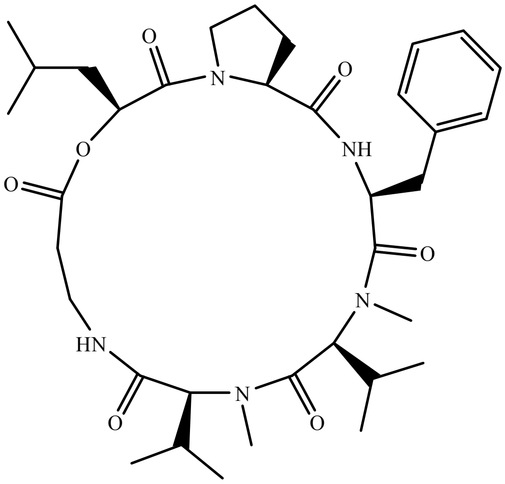

203

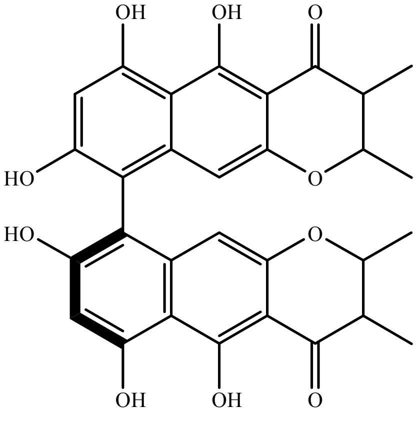

204

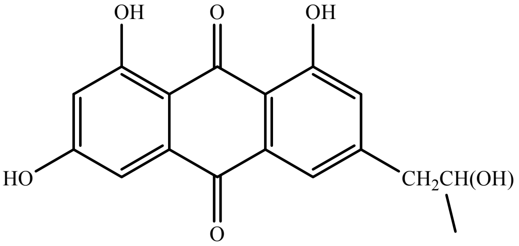

205

Figure S2. *Cont.*

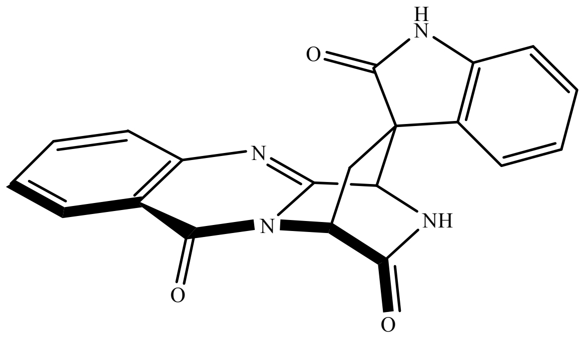

206

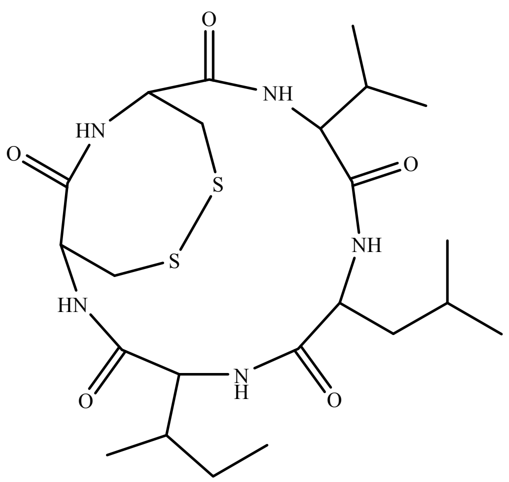

207

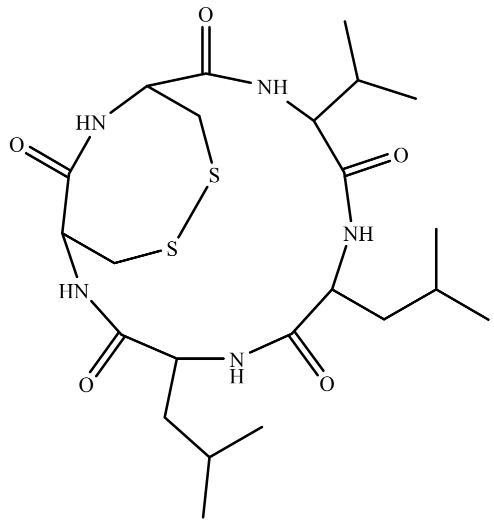

208

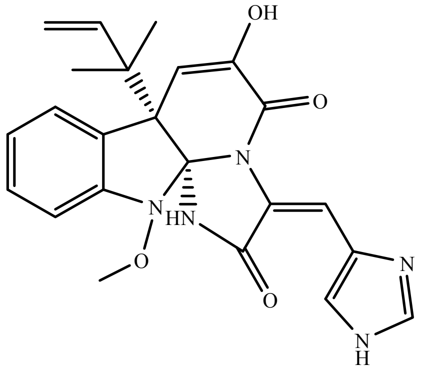

209

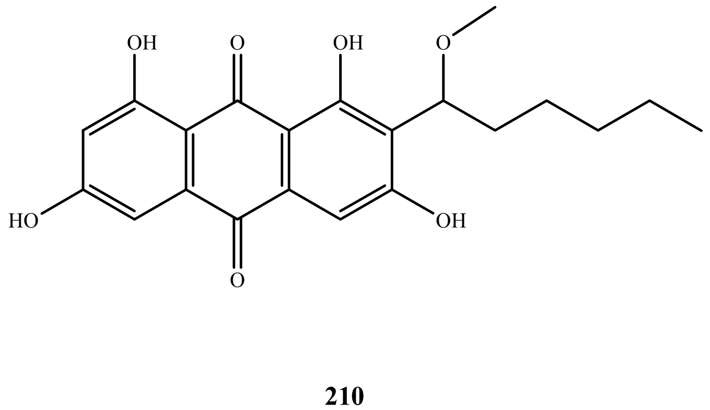

210

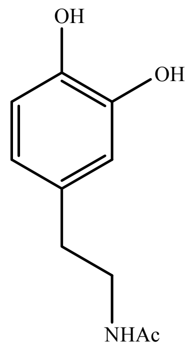

211

Figure S2. *Cont.*

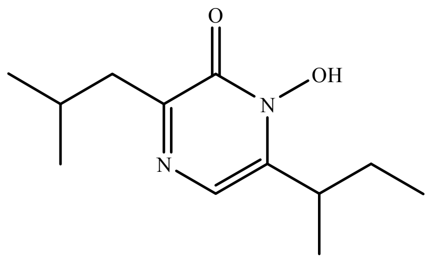

212

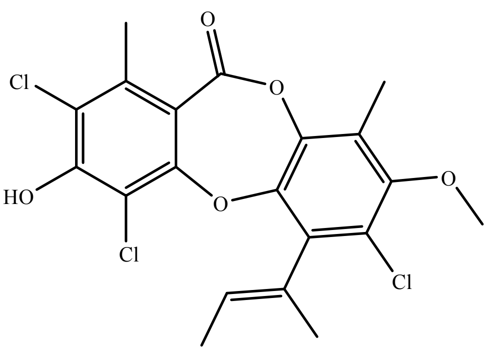

213

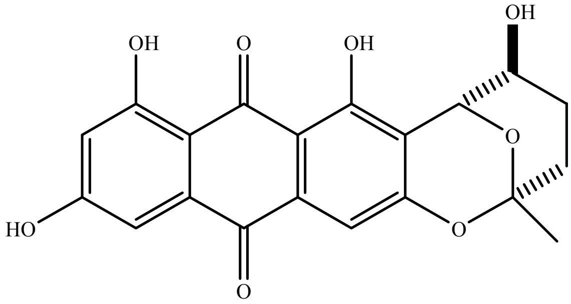

214

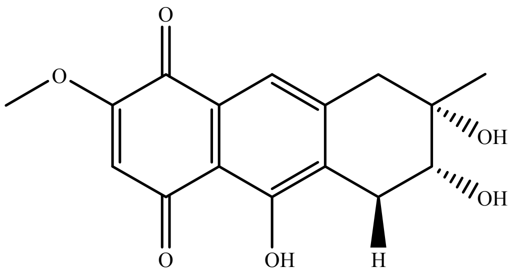

215

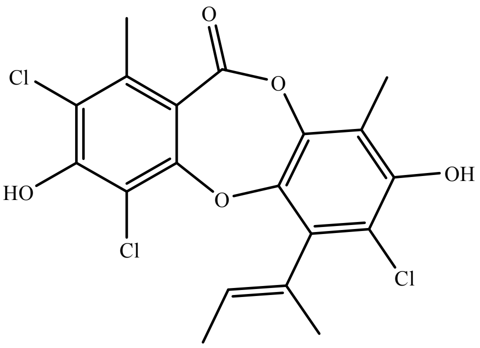

216

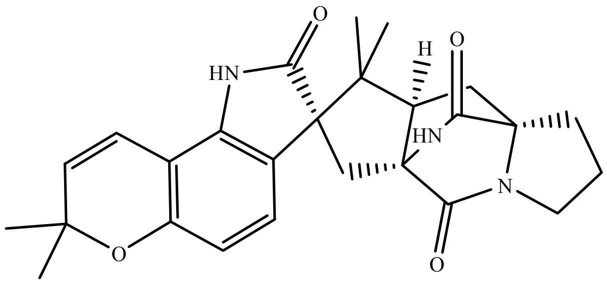

217

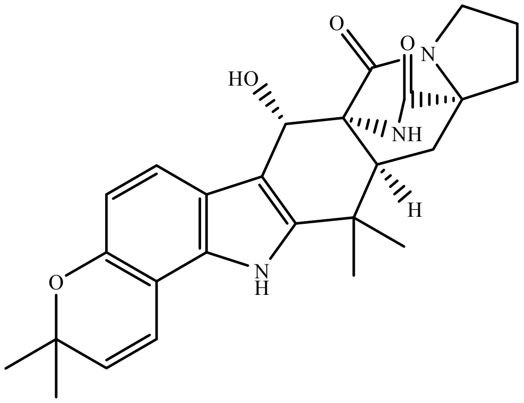

218

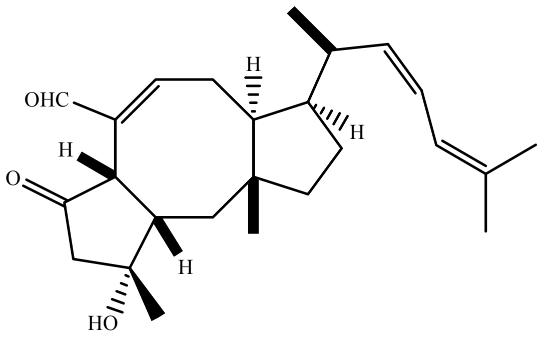

219

Figure S2. *Cont.*

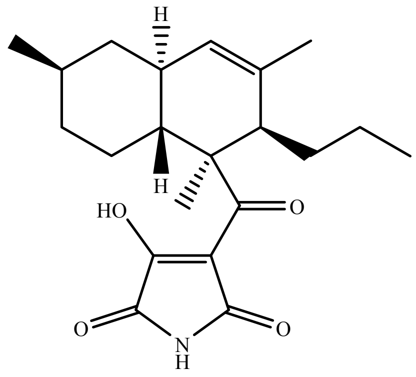

220

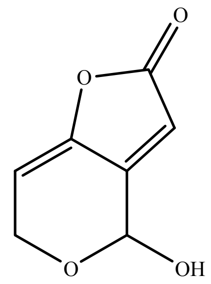

221

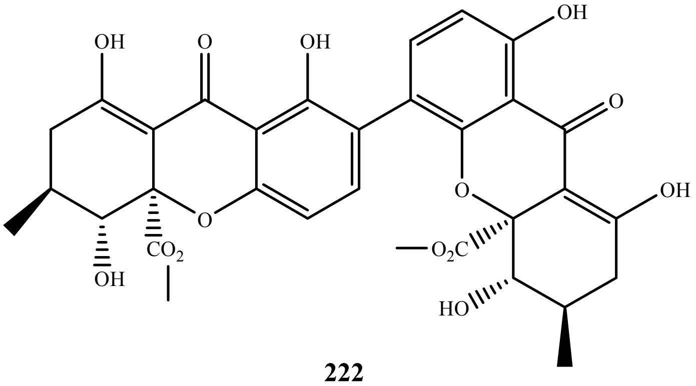

222

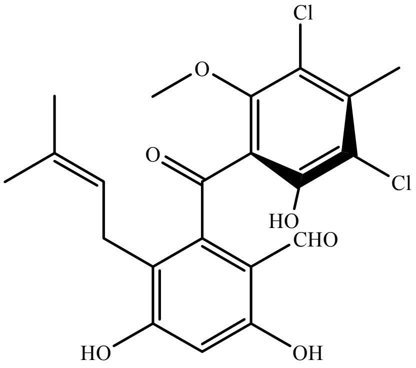

223

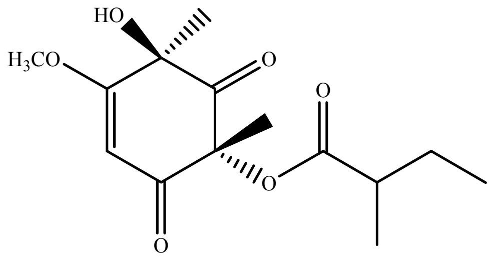

224

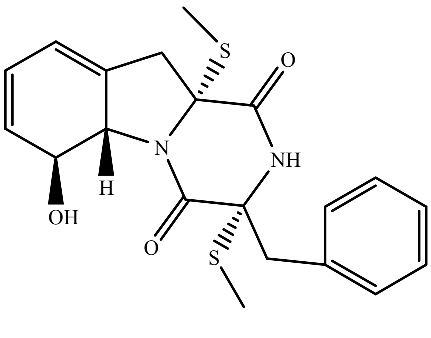

225

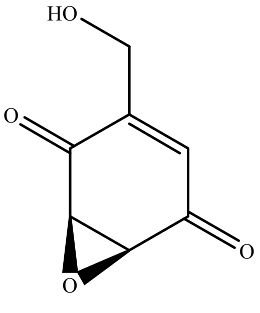

226

Figure S2. *Cont.*

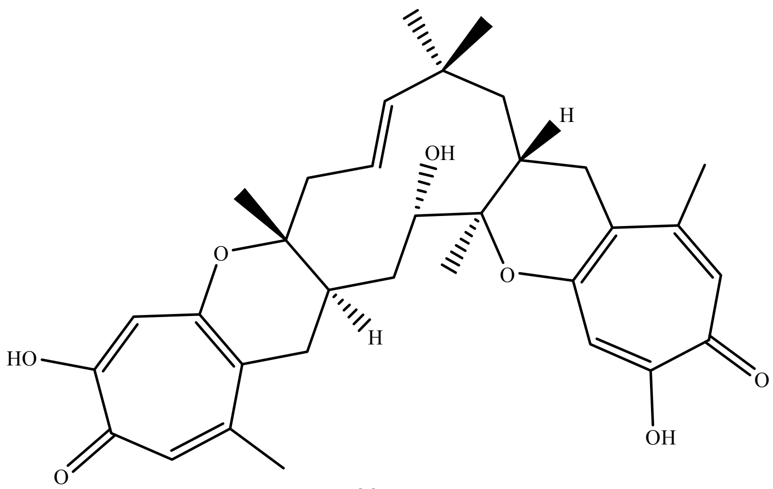

227

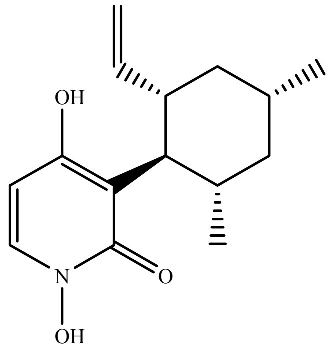

228

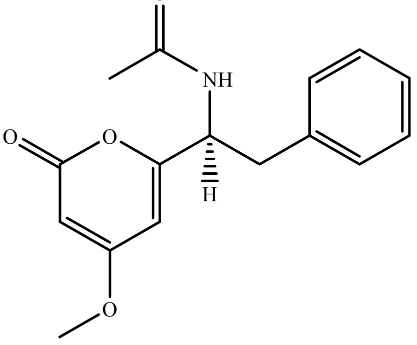

229

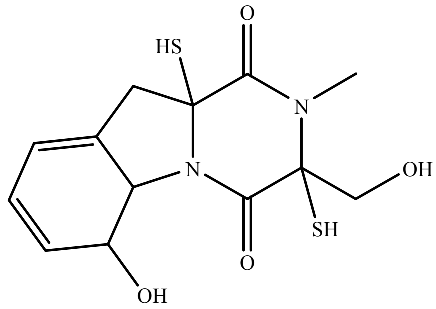

230

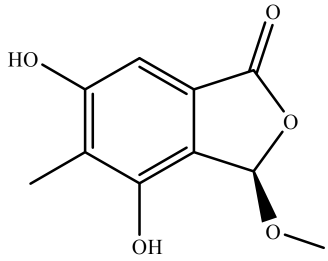

231

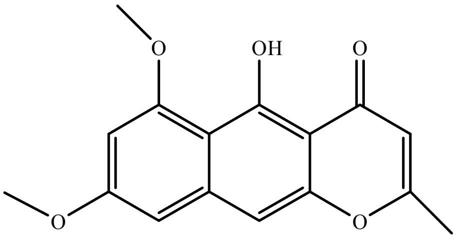

232

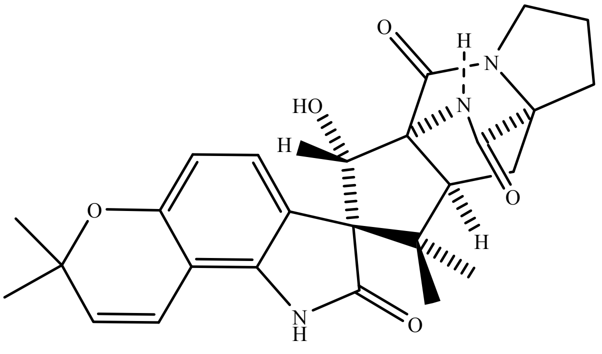

233

Figure S2. *Cont.*

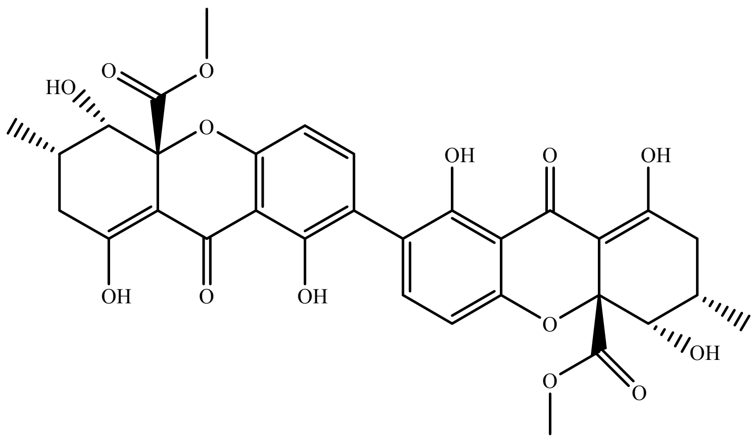

234

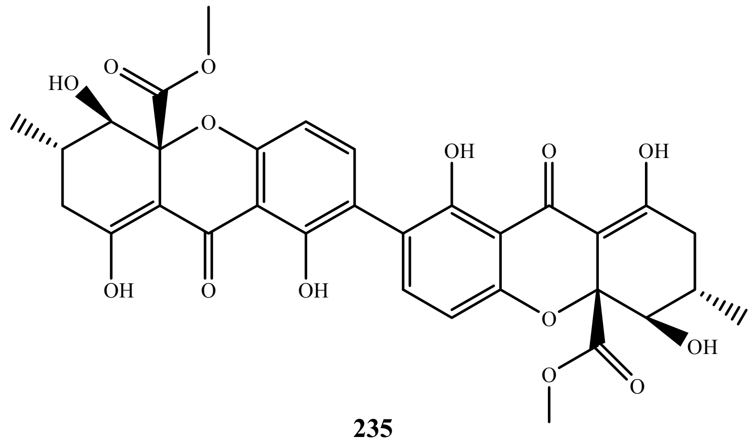

235

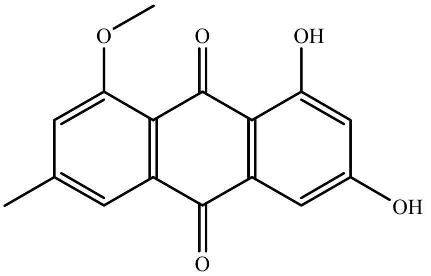

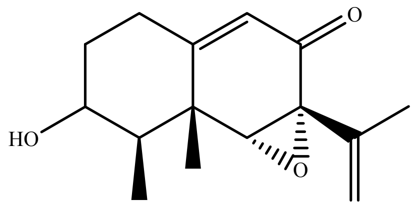

236 **237**

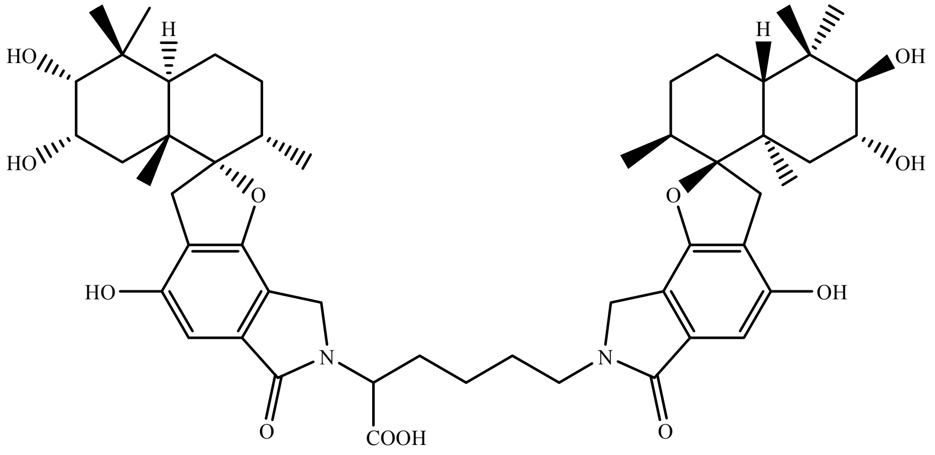

238

Figure S2. *Cont.*

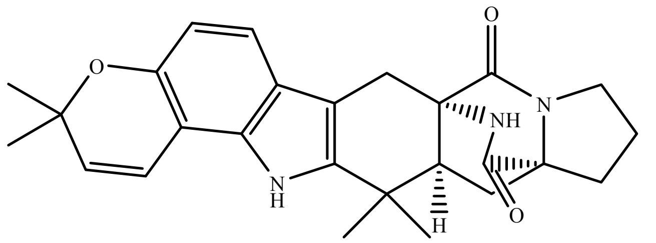

239

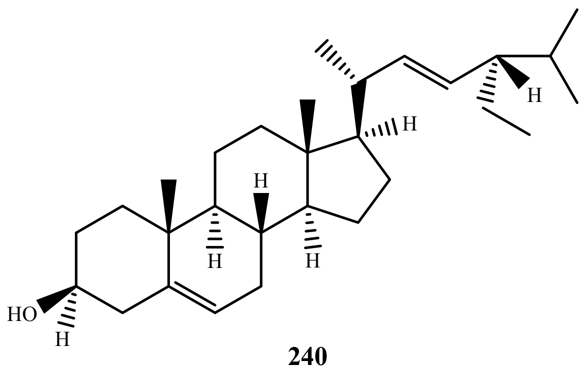

240

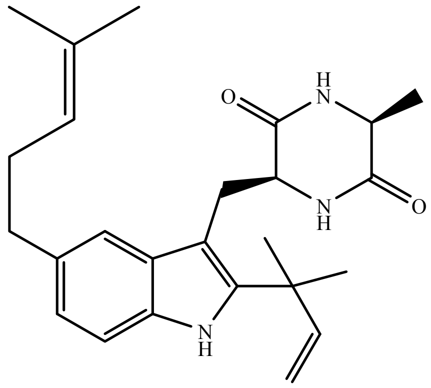

241

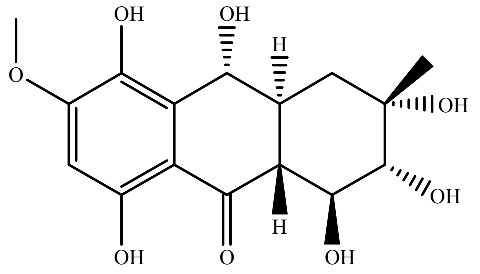

242

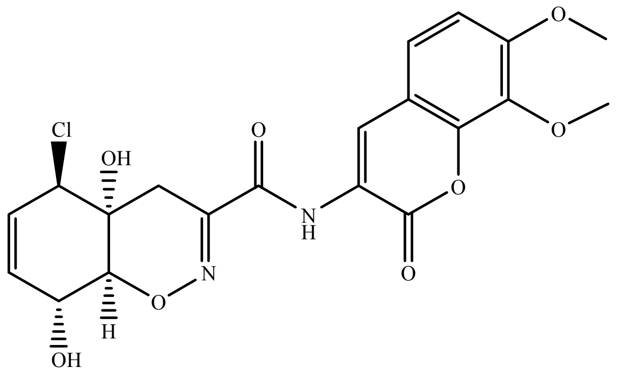

243

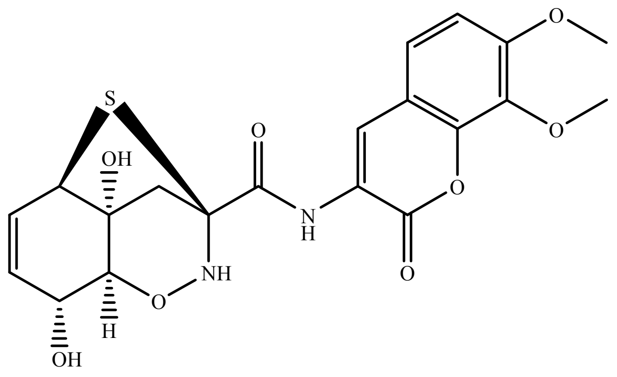

244

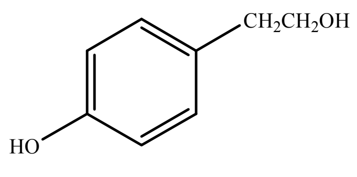

245

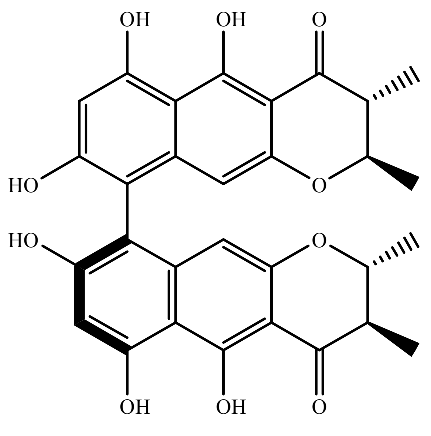

246

Figure S2. *Cont.*

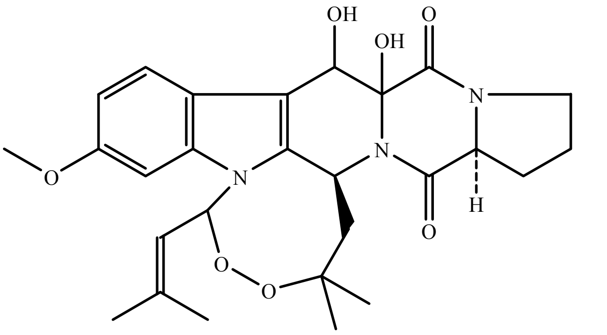

247

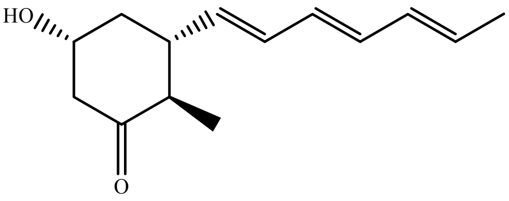

248

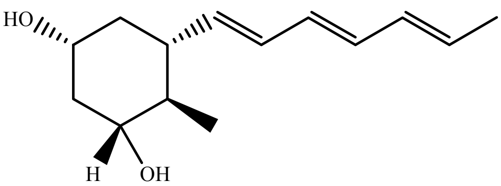

249

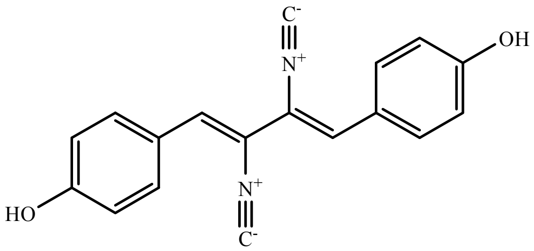

250

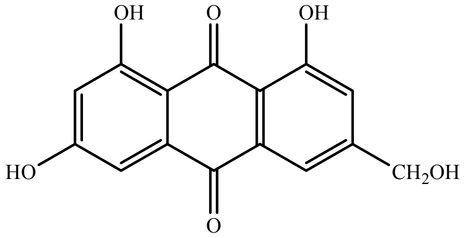

251

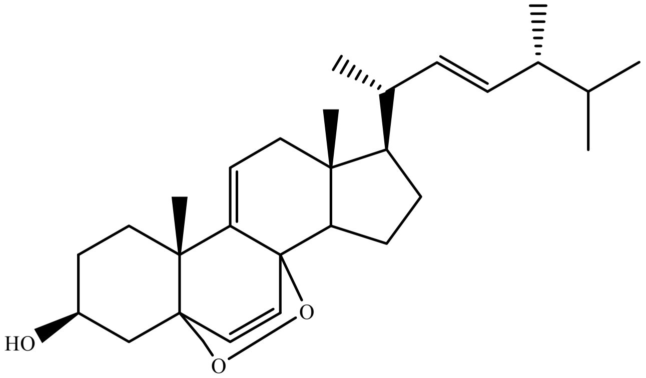

252

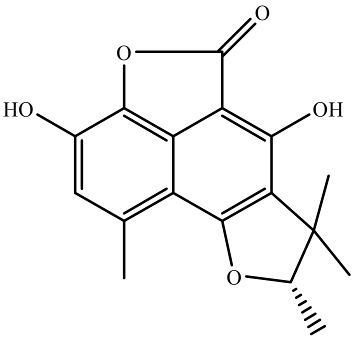

253

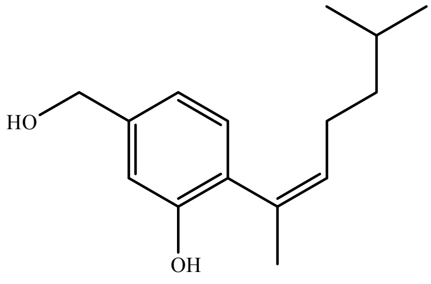

254

Figure S2. *Cont.*

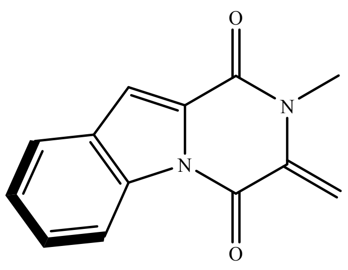

255

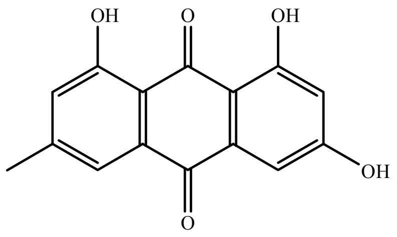

256

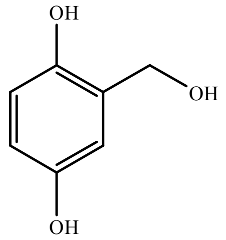

257

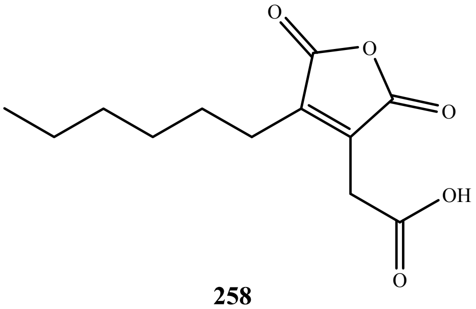

258

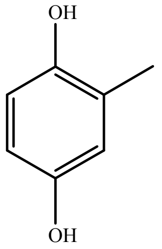

259

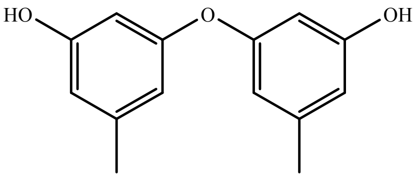

260

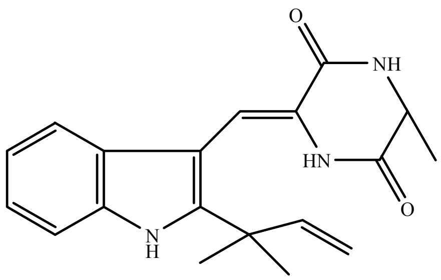

261

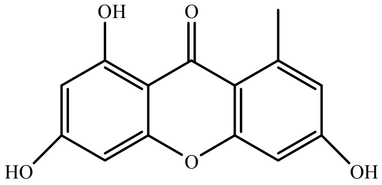

262

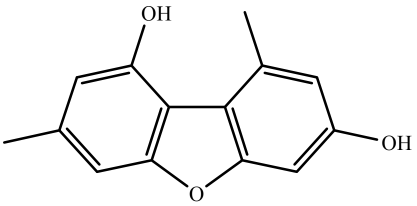

263

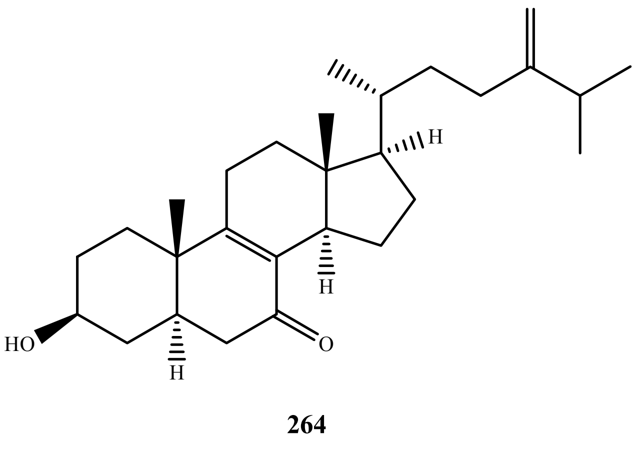

264

Figure S2. *Cont.*

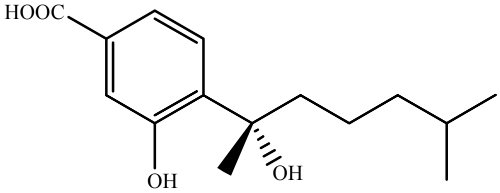

265

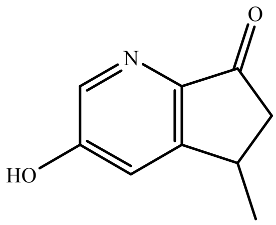

266

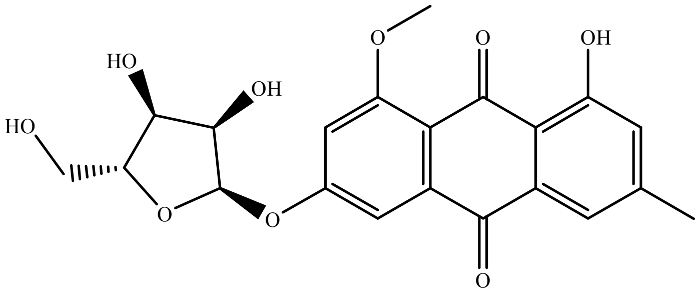

267

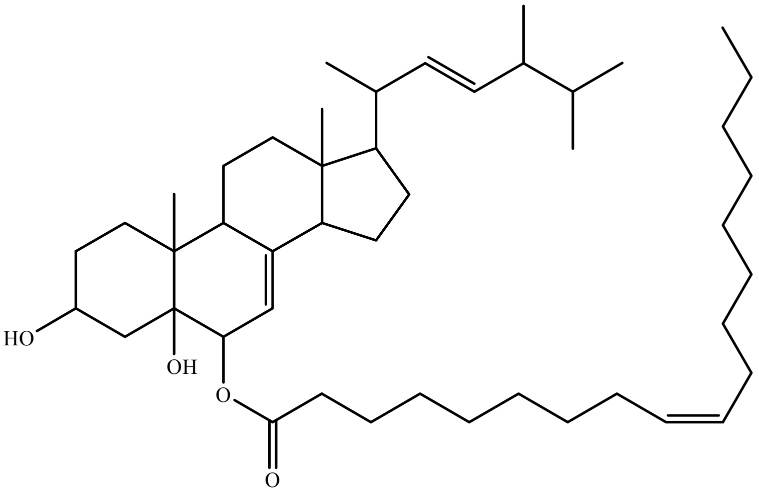

268

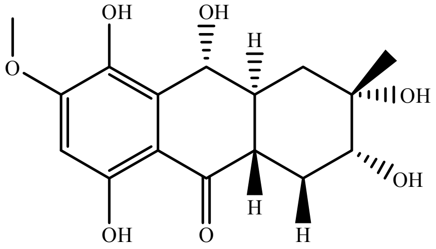

269

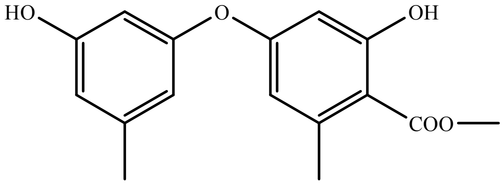

270

Figure S2. *Cont.*

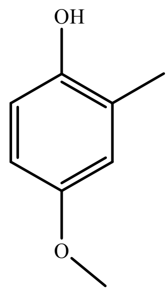

271

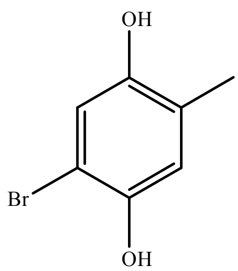

272

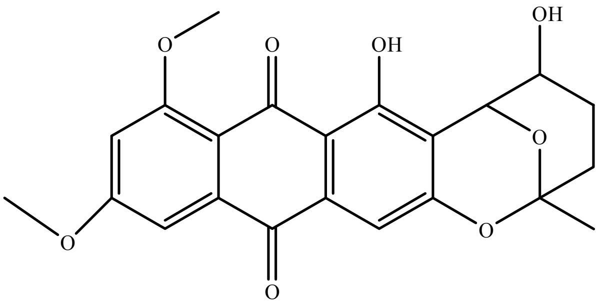

273

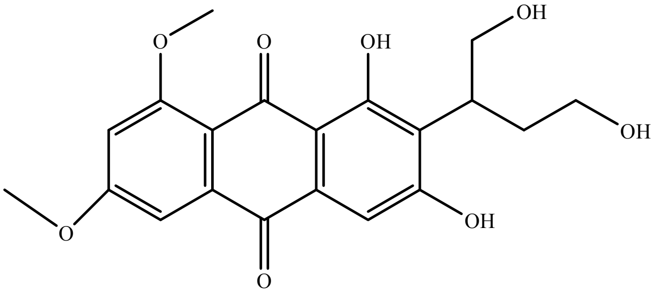

274

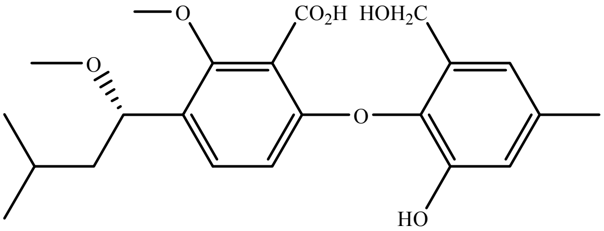

275

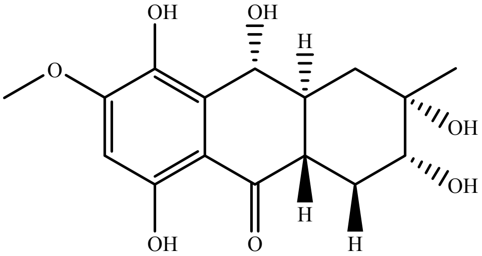

276

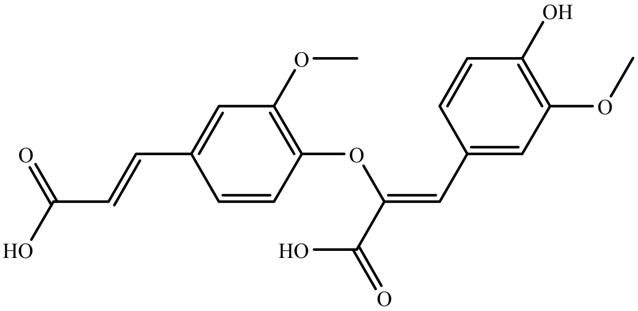

277

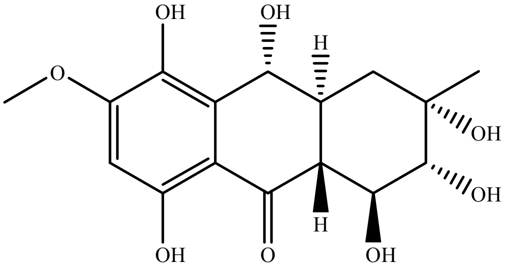

278

Figure S2. *Cont.*

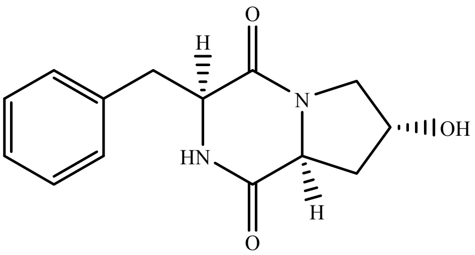

279

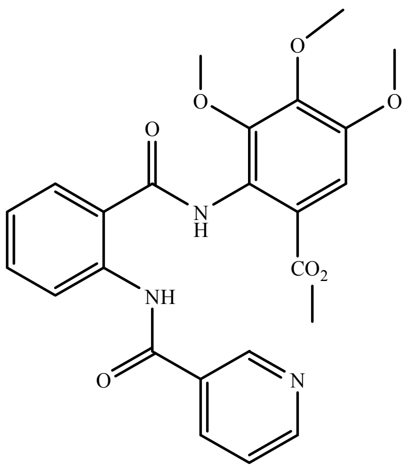

280

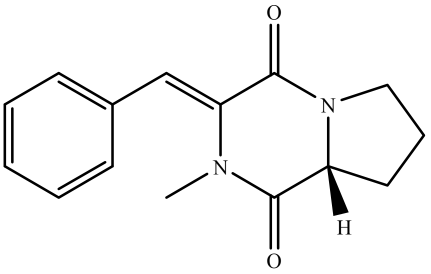

281

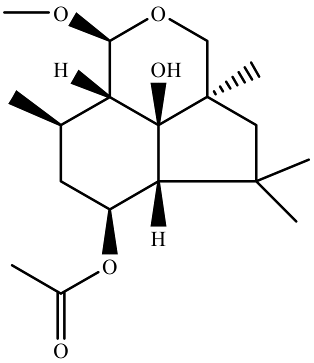

282

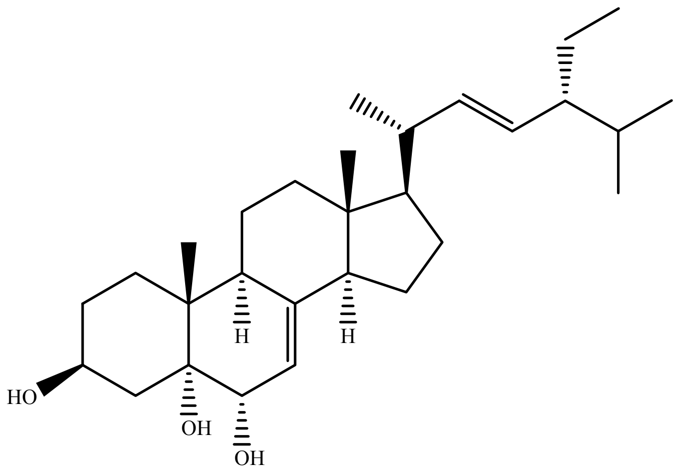

283

Figure S2. *Cont.*

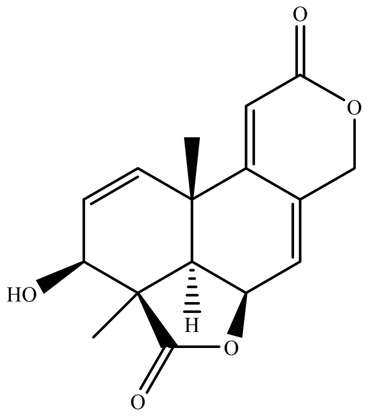

284

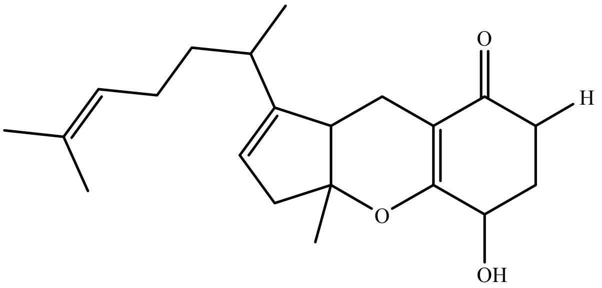

285

Figure S2. Structures of compound **117–285**.

© 2015 by the authors; licensee MDPI, Basel, Switzerland. This article is an open access article distributed under the terms and conditions of the Creative Commons Attribution license (http://creativecommons.org/licenses/by/4.0/).
